# Aluminum-[^18^F]fluoride radiolabeling of triarylphosphines for cell labeling via the perfluoroaryl azide Staudinger ligation

**DOI:** 10.1186/s41181-025-00409-9

**Published:** 2025-12-09

**Authors:** Anisa Biti, Surachet Imlimthan, Heidi Harjunpää, Diana Barakhtii, Topias Pöllänen, Arina Sukhova, Susanne K. Wiedmer, Filip S. Ekholm, Susanna Fagerholm, Mirkka Sarparanta

**Affiliations:** 1 Department of Chemistry, University of Helsinki, A.I. Virtasen aukio 1, 00560 Helsinki, Finland; 2 Molecular and Integrative Biosciences Research Programme, Faculty of Biological and Environmental Sciences, University of Helsinki, Viikinkaari 9, 00790 Helsinki, Finland

**Keywords:** In vivo cell tracking, Positron emission tomography (PET), Fluorine-18, Aluminum-[^18^F]fluoride, Metabolic glycoengineering (MGE), Bioorthogonal chemistry, Perfluoroaryl azide (PFAA), Staudinger ligation

## Abstract

**Background:**

The development of safer and more effective cell-based therapies requires robust methods for tracking cells in vivo. Positron emission tomography (PET) is a highly sensitive nuclear imaging technique capable of quantitatively tracking the in vivo fate of cells after administration. Here, we investigated a cell-labeling strategy based on metabolic glycoengineering (MGE) to introduce azides on the cell surface, followed by radiolabeling via the bioorthogonal perfluoroaryl azide (PFAA)-Staudinger reaction. We studied the metabolic incorporation of a tetraacetylated PFAA-derivatized mannosamine in Jurkat cells and evaluated whether three triarylphosphines bearing the REstrained Complexing Agent (RESCA), could be radiolabeled with aluminum-[F]fluoride (Al[^18^F]F) for use as bioorthogonal reagents in the PFAA-Staudinger ligation.

**Results:**

Three novel triarylphosphines containing different linkers (hydrophilic ester, ethylenediamine, and cyclohexyl) between the phosphine moiety and the (+)-RESCA-chelator were synthesized and characterized. Kinetic assays showed that all compounds reacted with the PFAA-derivatized monosaccharide, exhibiting different reaction kinetics under the tested conditions. They were successfully radiolabeled with fluorine-18 under optimized mild conditions and provided key insights into the radiolabeling of small molecules bearing the RESCA-chelator. The ester derivative underwent rapid chemical decomposition while the ethylenediamine- and cyclohexyl-linked derivatives were more resistant, with the cyclohexyl analogue showing the highest stability against demetallation and/or defluorination. However, radiolabeling with Al[^18^F]F led to the oxidation of the phosphine moiety, with the major radiolabeled product corresponding to the oxidized form. At the same time, flow cytometry showed that the metabolic incorporation of the tetra-acetylated PFAA-derivatized mannosamine into Jurkat cells was substantially less efficient than that of the widely used tetra-acetylated *N*-azidoacetylmannosamine (Ac_4_ManNAz) derivative at the equivalent concentration. Increased concentrations of the PFAA derivative compromised cell viability, which halted subsequent studies.

**Conclusions:**

While the Al[^18^F]F radiolabeling of these (+)-RESCA-bearing small molecules offers high stability against demetallation and/or defluorination, the method cannot currently be applied to triarylphosphines due to oxidation during radiolabeling and requires further development. Among the synthesized compounds, the cyclohexyl-linked derivative exhibited the most favorable stability profile, making it a potential lead structure for future tracer development. Nevertheless, this study advanced our understanding of MGE with PFAA-derivatized monosaccharides and highlighted the need for further investigation before applying PFAA-Staudinger ligation to cell radiolabeling.

**Supplementary Information:**

The online version contains supplementary material available at 10.1186/s41181-025-00409-9.

## Background

The non-invasive tracking of cells in vivo is critical for advancing cell-based therapies. Among various imaging modalities, positron emission tomography (PET) is currently the most suitable non-invasive imaging technique for tracking of radiolabeled cells after administration due to its high sensitivity, appropriate spatial resolution, and the capacity for direct quantification. To fully harness the advantages of PET for in vivo cell tracking, radiolabeling strategies must be carefully designed to ensure sufficient radiolabel stability while maintaining cell viability and function. Metabolic glycoengineering (MGE) is a well-established and robust method used for the functionalization of the cell surface that circumvents the need for genetic modification (Du et al. 2009; Wratil et al. 2016; Laughlin et al. 2006). MGE allows the incorporation of chemically reactive groups on the cell surface by incubating the cells with synthetic monosaccharide derivatives. MGE using azide-functionalized monosaccharides has been widely applied in fluorescent labeling (Hangauer and Bertozzi 2008; Kim et al. 2021; Cole et al. 2013) and nanoparticle-based cell labeling or in vivo delivery of different agents (Li et al. 2024; Koo et al. 2012; Wang et al. 2017). However, their application with radiolabeled probes, particularly small molecule bioorthogonal tracers remains scarce (Kim et al. 2021; Long et al. 2023; Lu et al. 2021). In the context of cell-based therapies, the application of MGE has been limited, with examples including targeted cell transplant for cancer therapy (Sutherland et al. 2024; Li et al. 2019), dendritic cell (DC) vaccine development (Han et al. 2023), and preliminary studies in mesenchymal stromal cell models (Altmann et al. 2021).

Azides are attractive chemical tags because of their small size, metabolic stability, and inertness towards native biomolecules. They function as labeling handles via biorthogonal reactions, transformations that proceed in biological systems with minimal perturbation of the endogenous chemistry (Debets et al. 2010; Zhang and Zhang 2013), and are therefore well-suited for cell labeling (Bird et al. 2021; Mitry et al. 2023; Prescher and Bertozzi 2005; Saxon and Bertozzi 2000). Azide-based bioorthogonal reactions include the copper-catalyzed azide-alkyne cycloaddition (CuAAC), the strain-promoted alkyne-azide cycloaddition (SPAAC), and the Staudinger ligation. In this work, we leverage the Staudinger ligation as our azide-reactive chemistry of choice.

**Fig. 1:**
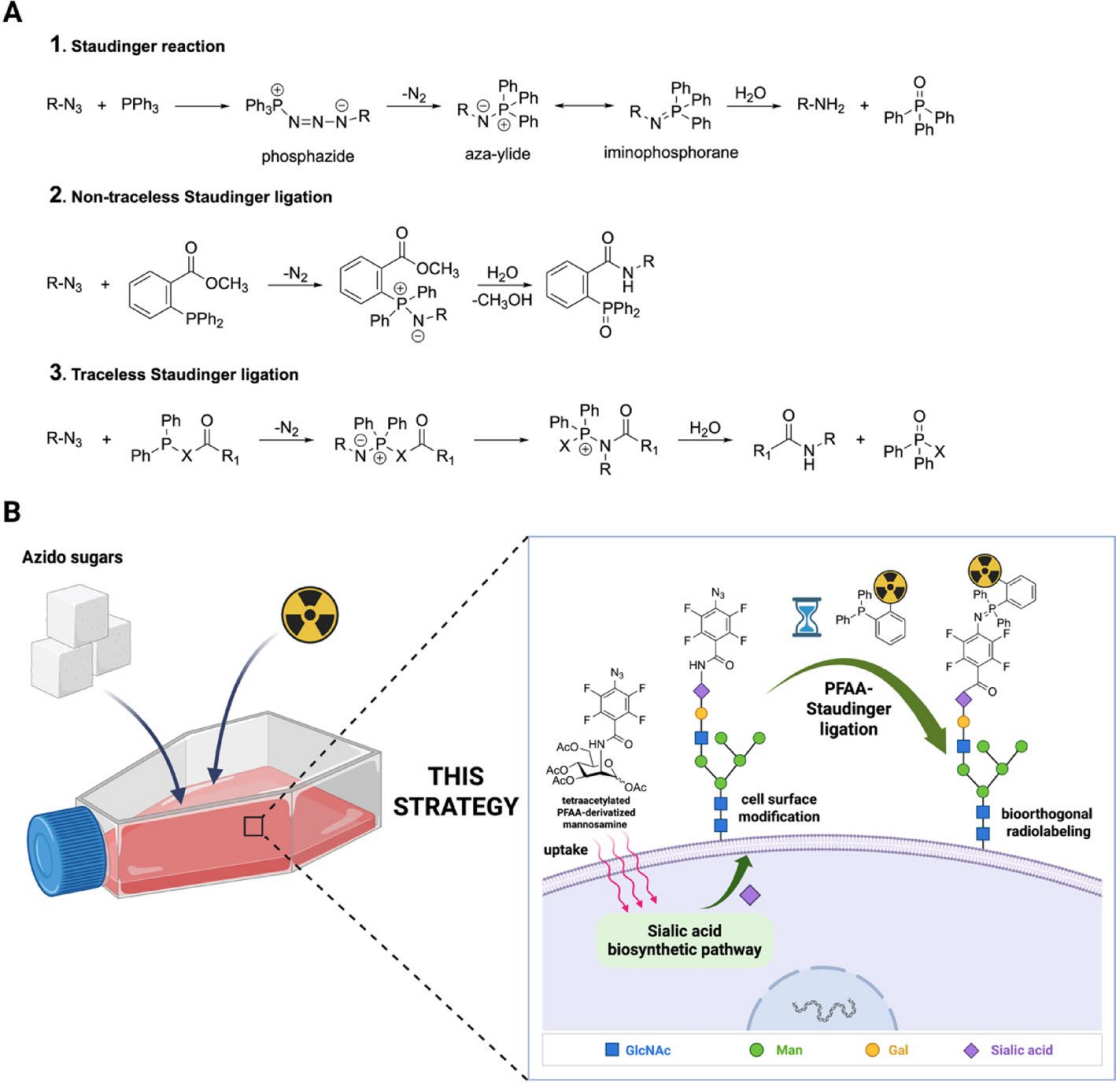
**A** The reaction mechanisms of classical Staudinger reaction (**1**), the non-traceless Staudinger ligation (**2**), and the traceless Staudinger ligation (**3**). **B** Cell radiolabeling via metabolic glycoengineering of the cell surface glycans with tetra-acetylated PFAA-derivatized mannosamine and the PFAA-Staudinger ligation with a radiolabeled triarylphosphine. The ligation yields a hydrolytically stable iminophosphorane. Created in BioRender. Biti, A. (2025) https://BioRender.com/e2ggx47

The Staudinger ligation, reported in 2000 by Saxon and Bertozzi, was the first reaction formally classified as bioorthogonal (Saxon and Bertozzi 2000). It derives from the classical Staudinger reaction shown in Fig. 1A1, originally described by Staudinger and Meyer in 1919, which is a reduction of azides to primary amines (Staudinger and Meyer 1919). The nucleophilic attack of the trivalent phosphorous to the terminal nitrogen of the azide generates a phosphazide, which decomposes by losing nitrogen (N_2_), forming an aza-ylide or iminophosphorane intermediate that rapidly hydrolyzes in aqueous conditions and yields a primary amine and a stable phosphine oxide. Since its first report, three versions of the Staudinger ligation have been developed, with differences in reaction mechanism, kinetics, and the structure of the final product (Fig. 1). The first reported variant uses phosphines bearing an electrophilic trap that captures the nucleophilic aza-ylide via intramolecular cyclization, yielding a stable amide bond instead of the hydrolysis products (Fig. 1A2). Because the phosphine oxide moiety remains covalently bound to the product, this variant is known as the non-traceless Staudinger ligation. In 2006, Bertozzi and co-workers reported a thorough investigation on the mechanism and kinetics of the reaction; when mediated by benzyl azide, the reaction proceeds with a second-order rate constant of 2.5 × 10^–3^ M^−1^ s^−1^ in ACN:H_2_O (19:1) (Lin et al. 2005). The second variant was reported soon after, simultaneously by Bertozzi (Saxon et al. 2000) and Raines (Nilsson et al. 2000). Here, the electrophilic trap is attached to the phosphine moiety through a cleavable bond. After the formation of the aza-ylide, the nucleophilic nitrogen of the aza-ylide attacks the carbonyl group to cleave the linkage and create the phosphonium species. The rearranged intermediate then undergoes hydrolysis, generating an amide and the phosphine oxide as a separate species (Fig. 1A3). Accordingly, this variant is known as the traceless Staudinger ligation. In this case, the reaction mediated by (diphenylphosphino)methanethiol proceeds with a second-order rate constant of 7.7 × 10^–3^ M^−1^ s^−1^ in DMF:D_2_O (6:1) (Soellner et al. 2006).

The kinetics of the Staudinger ligation can be improved by introducing electron-donating groups on the phosphine or electron-withdrawing groups on the azide (Lin et al. 2005). However, electron-rich phosphines are more susceptible to oxidation in the biological environment (Sletten and Bertozzi 2011), which substantially limits the biological applicability of Staudinger ligations. Efforts to accelerate reaction kinetics led to the development of perfluoroarylated azides (PFAAs). In 2017, Sundhoro and co-workers reported the PFAA-Staudinger ligation. The nucleophilic attack on the terminal nitrogen of the electrophilically-activated azide followed by N_2_ loss yields a hydrolytically stable iminophosphorane as the final product (Fig. 1B). The observed rate constant for a PFAA-mediated reaction reached up to 18.3 M^−1^ s^−1^ in CD_3_CN:D_2_O (1:1) (Sundhoro et al. 2017). In the same work, they demonstrated the potential of this reaction for cell labeling; MGE was used to install three tetra-acetylated PFAA-derivatized monosaccharides in A549 lung cancer cells, followed by the reaction with a biotin-conjugated triarylphosphine and detected labeling by fluorescent avidin by flow cytometry.

Since 2006, the combination of radionuclides and bioorthogonal chemistry has expanded the radiolabeling toolkit (Mindt et al. 2006). For the Staudinger ligation specifically, both radiolabeled organic azides and phosphines have been developed as radiolabeling precursors (Carroll et al. 2011; Mamat et al. 2011). Notable, in 2010 Pretze and co-workers attempted a palladium (Pd)-catalyzed synthesis of [^18^F]F-triarylphosphines from an [^18^F]F-iodophenyl ester; however, no desired product was obtained (Pretze et al. 2010a). Next, the use of [^18^F]fluoroethyl tosylate as a radiolabeling precursor enabled the first synthesis of ^18^F-triarylphosphines via alkylation of a triarylphosphine hydroxy derivative (Mamat et al. 2011). The first direct radiofluorination of a triarylphosphine using a tosylate precursor required harsh conditions (100 °C, 10 min) (Pretze et al. 2010b). In 2011, Vugts et al. attempted in vivo pretargeting of an azide-modified antibody via the non-traceless Staudinger ligation using four different triaylphosphines radiolabeled with gallium-67/68, zirconium-89, lutetium-177 and iodine-123. It was concluded that in vivo pretargeting using the non-traceless Staudinger ligation was not feasible (Vugts et al. 2011). The traceless Staudinger ligation was later used in a “click-to-chelate” approach to coordinate technetium-99 m with a tricarbonyl complex, but phosphine oxidation and nonspecific chelation led to multiple side products and required major optimization (Mamat et al. 2021). The latest application of the Staudinger ligation in radiolabeling is a “click-to-clear” strategy. An iodine-131-labeled, phosphine-modified antibody was administered to healthy and tumor-bearing mice and allowed to circulate, followed by an azide-bearing small molecule. Upon the in vivo Staudinger ligation, the radiolabeled residue of the ester electrophilic trap of the triarylphosphine, is cleaved and released into the bloodstream, promoting rapid systemic clearance of radioactivity and reducing radiation burden (Soni et al. 2023). To date, Staudinger ligations have not been applied to radiolabel cells after MGE, despite numerous examples in fluorescent labeling (Hangauer and Bertozzi 2008; Saxon and Bertozzi 2000; Chang et al. 2007; Prescher et al. 2004). We hypothesized that the superior kinetics of the PFAA-Staudinger ligation combined with MGE would be suitable for in vitro radiolabeling and PET tracking of radiolabeled cells with fluorine-18. A schematic overview of the metabolic glycoengineering and radiolabeling workflow is shown in Fig. 1B. To validate this strategy, we concurrently investigated, (1) the metabolic incorporation of a tetra-acetylated PFAA-derivatized mannosamine in our model cell system (Jurkat cells) and (2) the feasibility of radiolabeling three triarylphosphine derivatives with aluminum [^18^F]fluoride. The triarylphosphine precursors present the REstrained Complexing Agent (RESCA), a chelator specifically developed for Al[^18^F]F labeling of biomolecules under mild conditions (Cleeren et al. 2016, 2018), making it suitable for sensitive targets such as the oxidation-prone phosphines.

## Materials and methods

Detailed information on the materials, instrumentation, and parameters used to carry out the experiments and the software used to analyze and represent the data can be found in the Supplementary Information (SI).

### Chemistry

#### Synthesis and characterization of the chemical compounds

**Fig. 2:**
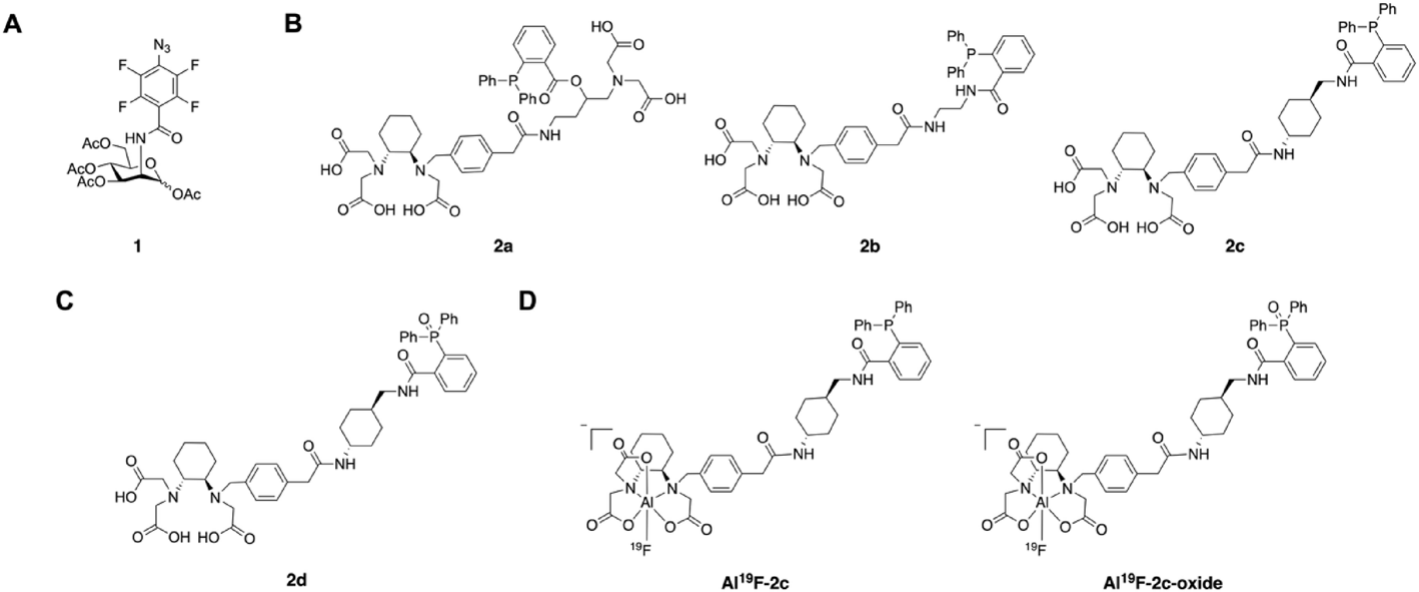
Chemical structures of **1** (**A**), **2a-2c (B)**, **2c-oxide** (**C**), **Al**^**19**^**F-2c,** and **Al**^**19**^**F**-**2c-oxide** (**D**)

The tetra-acetylated PFAA-derivatized mannosamine **1** (Fig. 2A) has been previously reported (Sundhoro et al. 2017) and was synthesized here using a modified procedure (Scheme S1) (Cole et al. 2013). The radiolabeling precursors ( +)-RESCA-triarylphosphines **2a**–**2c** (Fig. 2B) and reference compounds, **2c-oxide** (Fig. 2C), **Al**^**19**^**F-2c,** and **Al**^**19**^**F-2c-oxide** (Fig. 2D) were obtained via multi-step syntheses (Schemes S2–S1) and characterized using nuclear magnetic resonance (NMR) spectroscopy and/or high-resolution mass spectrometry (HRMS). The detailed procedures and characterization methods are provided in the SI.

#### Kinetic studies on the PFAA-Staudinger ligations of 2a-2c with 1 by ^1^H NMR spectroscopy

**Scheme 1:**
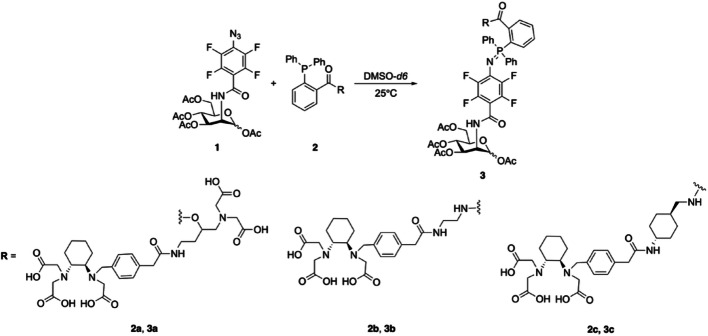
Reaction scheme for the Staudinger ligation between** 1** and **2a-2c** monitored by ^1^H NMR spectroscopy

The kinetics of the Staudinger ligation between **1** and **2a**–**2c** (Scheme 1) were monitored by ^1^H NMR spectroscopy, following spectral changes over time.

The kinetic experiments were carried out following a published protocol (Sundhoro et al. 2017). The compounds were weighed and dissolved in deuterated dimethyl sulfoxide (DMSO-*d*_*6*_) in an NMR tube (final concentration 2.5 mM; 0.4 mL reaction volume). ^1^H NMR spectra were recorded at 25 °C at intervals of 0.5–2 min, adjusted based on solubility and expected reaction times from pilot experiments. Kinetic models were evaluated by non-linear fitting corresponding to first- and second-order rate laws in Prism 10 (GraphPad, San Diego, CA, USA) as expected for the Staudinger ligation. Fitting was carried out using kinetic models based on reagent decomposition (A) or product formation (P) as shown in the equations below, depending on signal clarity and the magnitude of ^1^H NMR spectral changes over time:

$$ 1^{st} \;order: \left\{ {\begin{array}{*{20}c} {\left[ A \right] = \left[ A \right]_{0} \times e^{ - kt} } \\ {\left[ P \right] = \left[ P \right]_{0} \times \left( {1 - e^{ - kt} } \right)} \\ \end{array} } \right. $$

$$ 2^{nd} \;order: \left\{ {\begin{array}{*{20}l} {\left[ A \right] = \frac{{\left[ A \right]_{0} }}{{1 + k\left[ A \right]_{0} t}}} \hfill \\ {\left[ P \right] = \frac{{k\left[ A \right]_{0}^{2} t}}{{\left[ A \right]_{0} + k\left[ A \right]_{0} t}}} \hfill \\ \end{array} } \right. $$

where [A] = concentration of reactant A at time t, [A]₀ = initial concentration of reactant A (at t = 0), [P] = concentration of product P at time t, [P]₀ = maximum possible product concentration, k = rate constant and t = time elapsed.

#### Radiochemistry

**Scheme 2:**
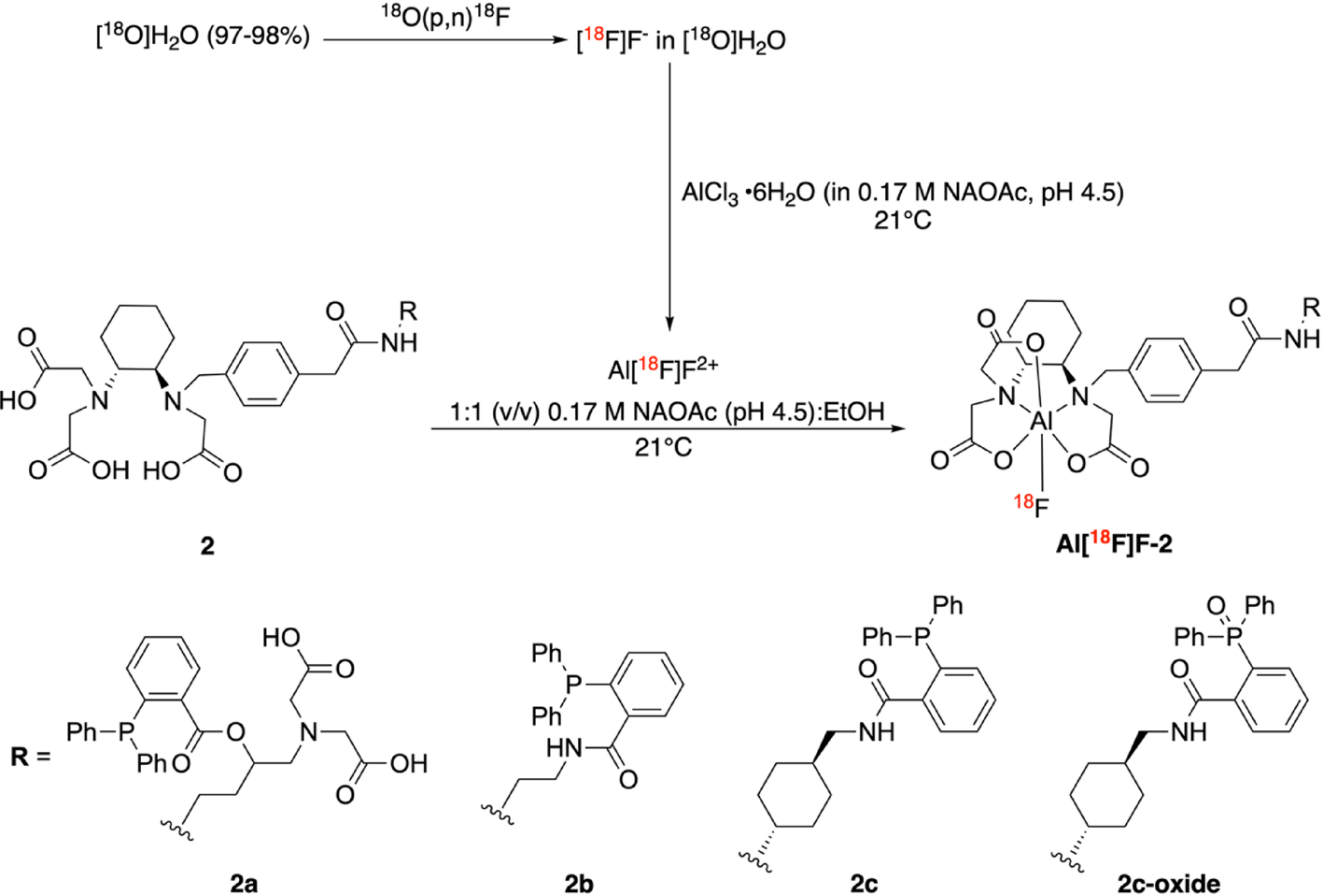
General scheme for the radiolabeling of precursors **2a–2c** and **2c-oxide** using the aluminum-[^18^F]fluoride (Al[^18^F]F) method

No-carrier-added [^18^F]fluoride was produced via the ^18^O(p,n)^18^F reaction by irradiating oxygen-18 enriched water ([^18^O]H_2_O) with 10 MeV protons on an IBA Cyclone 10/5 medical cyclotron (Louvain-la-Neuve, Belgium) in-house or with 18 MeV protons on an IBA Cyclone Kiube at the Cyclotron Unit, HUS Helsinki University Hospital. [^18^O]H_2_O was purchased from various vendors, including Rotem Industries (Arava, Israel; 98% isotopic enrichment), Campro Scientific (Berlin, Germany; 97% enrichment), and from Taiyo Nippon Sanso (Tokyo, Japan; 98% enrichment). The Al[^18^F]F-complexes were synthesized using a two-step procedure (Scheme 2).

First, 60 nmol of aluminum chloride (AlCl_3_, 30 μL of a 2 mM solution of aluminum chloride hexahydrate AlCl_3_·6H_2_O, 99.99%) in 0.17 M sodium acetate (NaOAc, pH 4.5) and 0.5 μL of glacial acetic acid were incubated with [^18^F]F^−^ (50–350 μL of [^18^O]H_2_O containing ca. 109–605 MBq) at ambient temperature (21 °C) for 10 min. Then, 90–180 nmol of precursor **2** (in 10 μL DMSO) dissolved in of 0.17 M NaOAc (pH 4.5):ethanol (EtOH) was added to achieve a final 1:1 (v/v) aqueous to organic solvent ratio (final volume 570–1000 μL). The reactions were incubated either at 21 °C or at 25–40 °C on a heating block for 15–45 min. During the optimization of the radiolabeling conditions, the reaction mixtures were analyzed using both radio-high performance liquid chromatography (radio-HPLC) and radio-instant thin layer chromatography (radio-iTLC), but the radiochemical conversion (RCC) was determined using only radio-iTLC as described by Herth and co-workers (Herth et al. 2021). Note that the radio-iTLC method used cannot distinguish free [^18^F]F^−^ from free Al[^18^F]F^2+^. All chromatograms shown in the main text were plotted from exported ASCII data files. Detailed chromatographic methods and examples of radio-TLC are given in the SI (Fig. S10–S20). **Al[**^**18**^**F]F**-**2a** was purified on an Oasis WAX solid-phase extraction (SPE) cartridge (Waters, Milford, MA, USA) preconditioned with EtOH, washed with 2% (v/v) formic acid (HCOOH), and eluted with 5% (v/v) ammonium hydroxide (NH_4_OH) in EtOH. **Al[**^**18**^**F]F-2b**, **Al[**^**18**^**F]F-2c**, and **Al[**^**18**^**F]F-2c-oxide** were purified on a Chromafix Alox N (Macherey–Nagel, Düren, Germany) or Sep-Pak Alumina N Plus Light Cartridge (Waters) without preconditioning and eluted with 1:1 (v/v) 1 × PBS (pH 7.4):EtOH. The isolated fractions were analyzed by radio-iTLC and radio-HPLC.

#### Radiolabel stability studies

The radiolabel stability of Al[^18^F]F-complexes was tested by diluting a sample of purified fractions (10–34 MBq) in different media, such as 1 × Hanks' balanced salt solution (HBSS, pH 7.2, n = 1–3), 2% fetal bovine serum (FBS) in 1 × HBSS (**Al[**^**18**^**F]F-2a**, n = 1), complete medium (RPMI 1640 supplemented with 10% FBS, 1% penicillin–streptomycin and 1% glutamine, **Al[**^**18**^**F]F-2c**, n = 1) followed by incubation at 37 °C. Samples were taken at 30, 60, 90, 120 min for **Al[**^**18**^**F]F-2b**, **Al[**^**18**^**F]F-2c**, and **Al[**^**18**^**F]F-2c-oxide** and up to 240 min for **Al[**^**18**^**F]F-2a**. Subsequently, samples were analyzed with radio-HPLC to detect the to detect the potential formation of other ^18^F-labeled chemical species and radio-iTLC to determine the degree of demetallation and/or defluorination.

#### LogD determination

The lipophilicity of Al[^18^F]F-complexes was determined with the shake-flask method as the distribution coefficient (logD) between aqueous phase represented by 0.01 M phosphate-buffered saline (PBS, pH 7.4) and 1-octanol (LogD_pH7.4_). A sample of the purified Al[^18^F]F-complexes (0.5–4 MBq) was first diluted in 1500–2500 μL 0.01 M PBS (pH 7.4) and aliquoted (500 μL, n = 2–4) into 1.5-mL microcentrifuge tubes containing 500 μL of 1-octanol to obtain a 1:1 (v/v) PBS pH 7.4:octanol mixture. The samples were incubated at 21 °C and vortexed every 10 min for 1 h and centrifuged (14 000 g, 10 min). Aliquots of 400 μL were taken from each phase and measured with a γ-counter. LogD_pH7.4_ was calculated as:

$${\text{LogD}}_{\text{pH}7.4}= \text{log}\left[\left(\frac{counts\, in\, octanol\, phase}{counts\, in\, aqueous\, phase}\right)\right]$$

#### The bioorthogonal reactions

**Scheme 3:**
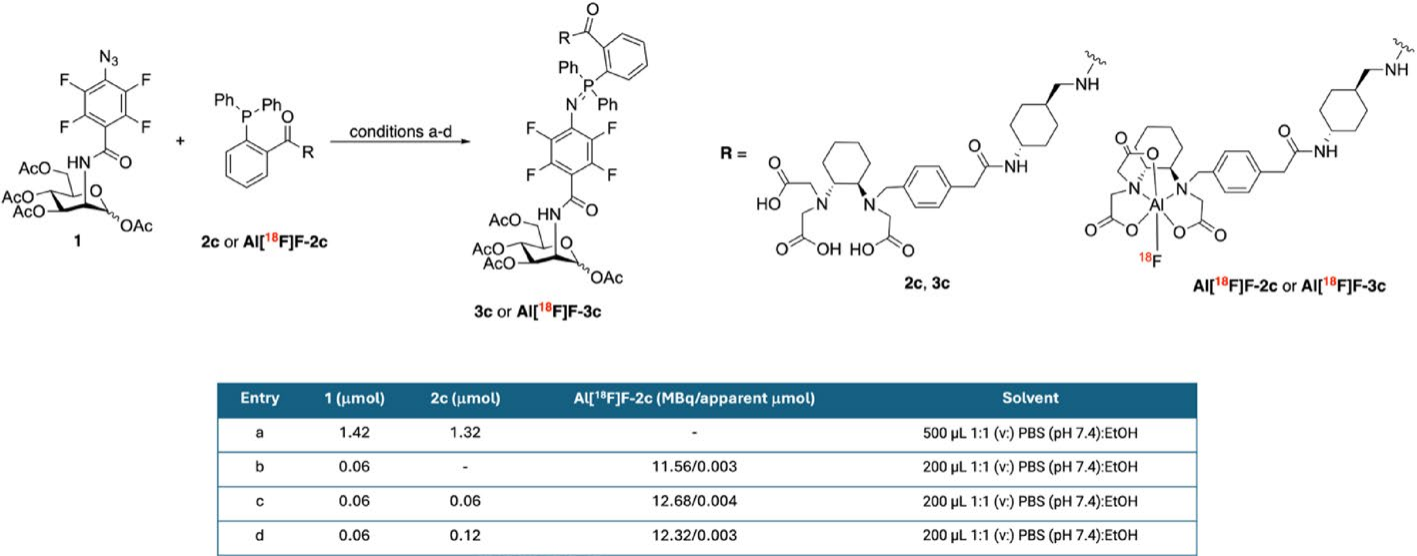
Reaction scheme and conditions **a–d** for the Staudinger ligations between** 1** and ( +)-RESCA-triarylphosphines **2c** and **Al[**^**18**^**F]F-2c**

The Staudinger ligation between **1** and either **2c** or **Al[**^**1**^**⁸F]F-2c** (Scheme 3) was investigated using different amounts of the bioorthogonal partners as summarized in the table in Scheme 3.

All reactions were carried out at 37 °C and analyzed after 30 min by HPLC. For entry **a**, the reaction mixture was additionally incubated overnight at 37 °C and reanalyzed using both HPLC and liquid chromatography-mass spectrometry (LC–MS). Detailed information on the HPLC and LC methods can be found in the SI. The maximum apparent amount (μmol) of **Al[**^**18**^**F]F-2c** corresponding to the activity in MBq was calculated using the activity of the reaction mixture and the RCC at 30 min reaction time.

### Comparative HPLC analysis of 2c, 2c-oxide, and their Al^19^F- and Al[^18^F]F-complexes

The analysis was carried out using a pentafluorophenyl (PFP) column (Kinetex 2.6 µm PFP 100 Å, LC Column 150 × 4.6 mm from Phenomenex, Torrance, CA, USA) and HPLC methods A and B (see SI). The Al^19^F-complexes were isolated and analyzed using NMR and/or HRMS. The stability of **2c** was also tested in the presence of 60 nmol AlCl_3_ in 1:1 (v/v) 0.17 M NaOAC (pH 4.5):EtOH, simulating the radiolabeling conditions.

### Cytotoxicity studies

The in vitro cytotoxicity of **1** was assessed using the CyQUANT™ LDH Cytotoxicity Assay (Invitrogen, Waltham, MA, USA), which quantifies membrane damage via extracellular lactate dehydrogenase (LDH) release. Assay reagents (Lysis Buffer, Reaction Mixture, and Stop Solution) were prepared according to the manufacturer’s protocol. Human Jurkat T lymphoblasts (Clone E6-1, ATCC TIB-152™, Manassas, VA, USA) were suspended in complete medium and mixed (1:1) with **1** in complete medium to obtain final concentrations of 10, 20, 50, 125, and 250 μM containing 0.5% v/v DMSO vehicle. Positive control for cytotoxicity (maximum LDH; cells lysed with 10 × Lysis Buffer at 1:10 v/v, prepared before each time point), negative control for cytotoxicity (spontaneous LDH; 0.5% v/v DMSO in complete medium), and complete-medium and serum-free medium blanks (background) were included. Sample-containing and control cell suspensions were plates at a density of 14,000 cells per well in 280 μL on an opaque-walled, clear-bottom 96-well plate. Plates were incubated at 37 °C, 5% CO_2_ in a humidified incubator for 24, 48, and 72 h. All conditions were run in quadruplicate (n = 4). Prior to each time point, the positive control was prepared by withdrawing 80 μL of cell suspension from designated well into a clean LoBind tube, adding 8 μL of 10 × Lysis Buffer, vortexing briefly, and returning the tube to the incubator for 45 min. At the time point, 80 μL of cell suspension from each experimental and control well (excluding the positive control) was transferred to 1.5-mL LoBind tubes. All samples were centrifuged at 4 °C, 425 × g for 10 min. The supernatant (50 μL) was dispensed into a transparent, flat-bottom 96-well plate. Reaction Mixture (50 μL) was added to each well, gently mixed, and incubated for 30 min at ambient temperature protected from light (aluminum foil), followed by 50 μL of Stop Solution before measurement. Absorbance was measured at 490 and 680 nm on a Synergy H1 multimode microplate reader (BioTek, Winooski, VT, USA). LDH signal was calculated as Abs_490_ – Abs_680_ (background-corrected).

% Cytotoxicity was determined as:

$${\% Cytotoxicity}= \frac{\left({\text{LDH}}_{\text{compound}}\right)-({\text{LDH}}_{\text{spontaneous}})}{\left({\text{LDH}}_{\text{maximum}}\right)-({\text{LDH}}_{\text{spontaneous}})}\times 100$$

% Cell viability was reported as:

$${\% Cell\;viability}=100-\% \text{Cytotoxicity}$$

### Flow cytometry

Jurkat cells (5 × 10^5^ cells in 1.5-mL complete medium per well) were seeded in a non-treated flat-bottom polystyrene 6-well plate (Avantor, Radnor, PA, USA) and incubated in triplicates (n = 3) with 50 μM of **1**, 50 μM of Ac_4_ManNAz (Lumiprobe, Westminster, MD, USA) or 0.5% (v/v) DMSO in complete medium for 72 h in a cell incubator set at 37 ºC with 5% CO_2_ and 95% relative humidity. After incubation, the cells for each condition were combined and washed twice with 5 mL of 1 × Dulbecco's phosphate-buffered saline (DPBS). The cell number and viability were determined using EVE™ automatic cell counter (NanoEnTek, Seoul, Korea). 1 × 10^6^ cells were resuspended in 500 µL of a freshly prepared 1 × DPBS (pH 7.4) or 1 µM of phosphine-PEG3-biotin (Thermo Fisher Scientific, Waltham, MA, USA) in 1 × DPBS (n = 3), then transferred to 1.5-mL microtubes, and incubated at ambient temperature for 60 min on a rotating platform. Then, cells are washed twice with 1 × DPBS, resuspended in 100 µL of a freshly prepared solution of 1:200 streptavidin-fluorescein isothiocyanate (FITC) (0.1 μg, Thermo Fisher Scientific, Waltham, MA, USA) in 1 × DPBS, and incubated for 15 min in the dark on ice with mixing once manually in between. After the incubation, 400 µL of FACS (fluorescence-activated cell sorting) buffer (2% FBS in DPBS) was added followed by two washings with 500 µL of FACS buffer. During the washing step, the samples were centrifuged at 4 °C, 10 × g for 5 min, the supernatant was removed, and the cells were resuspended in fresh buffer, followed by centrifugation. Lastly, cells were resuspended in 500 µL of FACS buffer and transferred to FACS tubes equipped with a 35 µm cell strainer cap. Dead and apoptotic cells were stained by adding 5 µL of 7-aminoactinomycin D (7-AAD) staining solution and incubating for 10 min at ambient temperature in the dark, followed by flow cytometry analysis using LSR Fortessa (BD Biosciences, Franklin Lake, NJ, USA). Unstained samples, samples stained only with streptavidin-FITC, and only with 7-AAD were used as controls. Data were analyzed using FlowJo software (FlowJo LLC, Ashland, OR, United States).

## Results

### Synthesis and characterization of the chemical compounds

The tetra-acetylated PFAA-derivatized mannosamine **1**, the radiolabeling precursors **2a-2c** and **2c-oxide**, the complexes **Al**^**19**^**F-2c** and **Al**^**19**^**F-2c-oxide** were successfully obtained with an acceptable yield and an appropriate chemical purity (≥ 95%). Compound **1** was analyzed by ^1^H, ^13^C ^19^F NMR spectroscopy and HRMS, whereas compounds **2a–2c**, **2c-oxide**, and **Al**^**1**^**⁹F-2c** were additionally characterized by ^31^P NMR. **Al**^**1**^**⁹F-2c-oxide** was analyzed only by HRMS. **3c** was analyzed only by LC–MS. The characterization data can be found in the SI (NMR spectra in Figures S22–45 and HRMS data in Table S1).

### Kinetic studies on the PFAA-Staudinger ligations of 2a-2c with 1 by NMR spectroscopy

Under the tested conditions, the PFAA-Staudinger ligations of **2a** and **2c** followed second-order kinetics with the determined constant values of 4.246 × 10^–4^ M⁻1 s⁻1 and 4.071 × 10^–4^ M⁻1 s⁻1, respectively. In contrast, **2b** demonstrated first-order kinetics with a constant value of 5.434 × 10^–3^ s⁻1, an order of magnitude higher. The correlation coefficients (R^2^) for **2a** and **2b** were 0.93 and 0.99, respectively, whereas the best R^2^ obtained for **2c** was only 0.74, regardless of the model applied. The second-order fitting provided a slightly better correlation with the data and was therefore used to represent the reaction kinetics and calculate the apparent rate constant. The curve fittings are provided in the SI (Fig. S1) together with the ^1^H NMR spectra showing the progression of the reactions (Fig. S2–S4).

### Radiochemistry

#### Radiolabeling of 2a

The highest RCC was achieved with 2 equivalents of precursor **2a** (120 nmol) relative to AlCl_3_ (60 nmol) at 21 °C for 30 min (entry **d**, Table 1). Mild heating (25–30 °C) had minimal impact on the RCC (entries **f–h**, Table 1), whereas 35–40 °C yielded variable RCCs (entries **i** and **j**, Table 2). Further incubation at 21 °C for 75 min increased the RCC to 82.8% for entry **i** and 86.9% for entry **j.** The RCC was calculated as mean ± s.d. for replicate experiments (n > 1). The isolated product after Oasis WAX SPE cartridge purification showed radiochemical purity (RCP) > 98% by radio-iTLC in all cases.

**Table 1:** Radiolabeling conditions and RCCs for **Al[**^**18**^**F]F-2a**. RCC values represent mean ± s.d. for experiments carried out in replicates (n > 1)

Precursor	Entry	n	Activity at SOS (MBq)	n_precursor_ (nmol)	Temperature (°C)	Volume (µL)	Time (min)	RCC (%)
**2a**	**a**	1	234	90	21	770	25	70.3
	**b**	1	212	180	21	970	30	52.5
							40	67.3
							45	74.9
	**c**	5	82–256	120	21	770	25	88.1 ± 2.3
	**d**	2	182–216	120	21	970	30	93.2 ± 2.7
	**e**	1	149	120	21	870	30	87.2
							40	85.5
							45	84.1
	**f**	1	145	120	25	770	30	92
	**g**	1	150	120	25	770	45	85.9
	**h**	1	199	120	30	820	25	92
	**i**	1	233	120	35	970	25	3.5
							30	10.5
	**j**	1	145	120	40	870	25	7.2
							30	90.2

**Table 2:** Radiolabeling conditions and RCCs for **Al[**^**18**^**F]F-2b**, **Al[**^**18**^**F]F-2c** and **Al[**^**18**^**F]F-2c-oxide**. RCC values represent mean ± s.d. for experiments performed in replicate (n > 1)

Precursor	Entry	n	Activity at SOS (MBq)	n_precursor_ (nmol)	Temperature (°C)	Volume (µL)	Time (min)	RCC (%)
**2b**	**a**	1	327	120	21	570	30	97.1
	**b**	1	221	120	21	1000	30	94.9
**2c**	**c**	1	137	120	30	970	30	3.0
							40	5.6
	**d**	1	109	120	21	970	30	43.6
							45	44.5
	**e**	22	109–605	120	21	640–670	30	93.2 ± 8.3
**2c-oxide**	**f**	10	132–576	120	21	670–790	30	97.7 ± 1.5

#### Radiolabeling of 2b, 2c and 2c-oxide

Only two radiolabeling trials were conducted with **2b** (entries **a** and **b**, Table 2), differing primarily in the total volume of the reaction mixture. For **2c**, decreasing the reaction volume from 980 μL to 620–670 μL, while maintaining reaction time, temperature, and precursor amount constant, increased RCC from 44.5% to > 95% (entry **e**, Table 2). Varying the starting activity did not influence the RCC. The optimized radiolabeling conditions were then applied to the reference compound **2c-oxide**, with 10 radiolabeling trials carried out (entry **f**, Table 2). The RCC was calculated as mean ± s.d. for experiments performed in replicate (n > 1). The Al[^18^F]F-complexes were obtained with a high RCP (95–99%) after purification on Chromafix Alox N or Sep-Pak Alumina N Plus Light cartridges.

#### Radiolabel stability studies and LogD determination

**Fig. 3:**
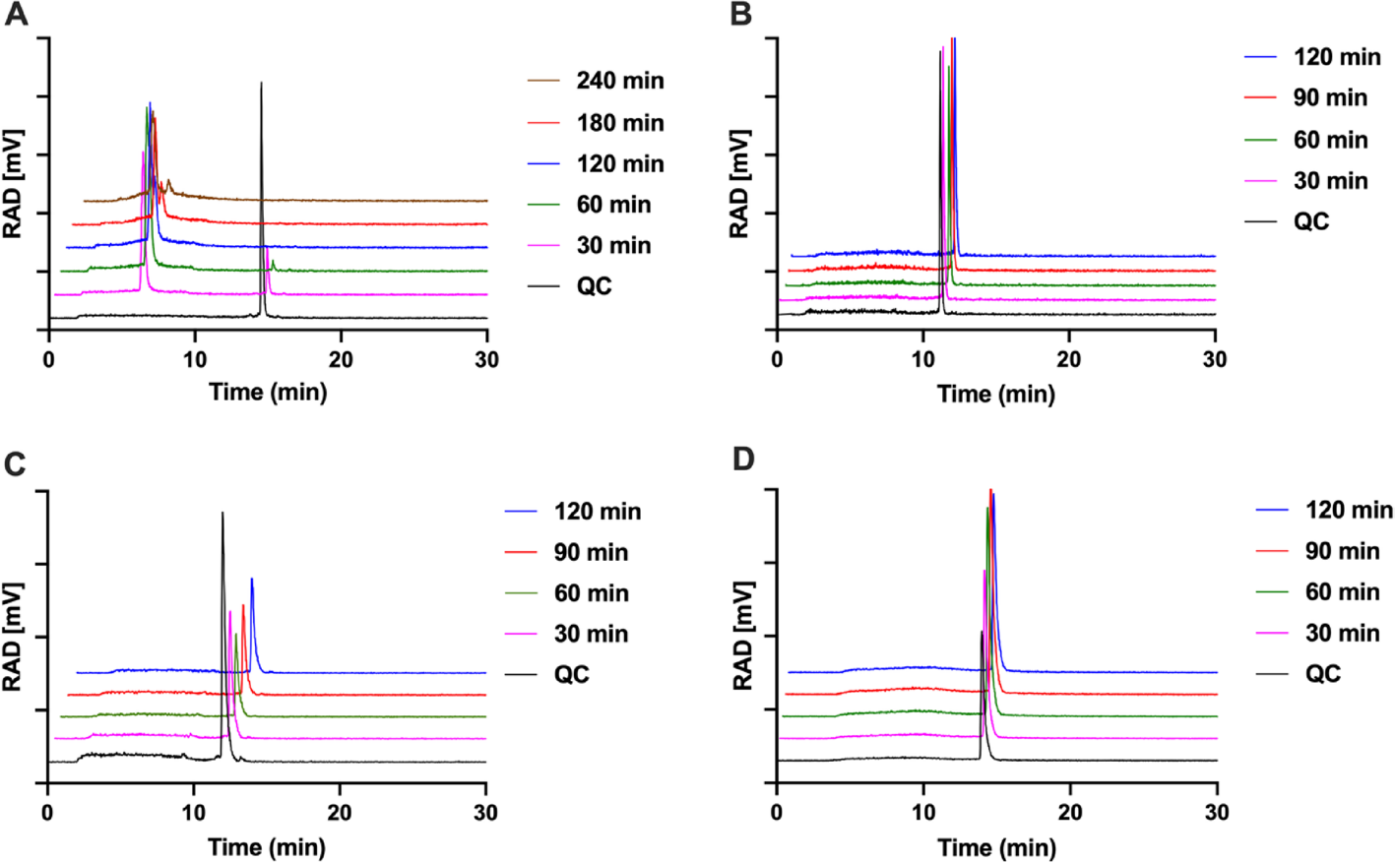
Radio-HPLC radiolabel stability assays in 1 × HBSS. In radio-HPLC, the chromatogram in black shows the purified product in the quality control (QC) sample, and the colored chromatograms represent the radio-HPLC analysis at predetermined time points. **A**. **Al[**^**18**^**F]F-2a** in 1 × HBSS at 37 °C: intact product retention time 14.58 min and decomposition products 5–7 min. **B**. **Al[**^**18**^**F]F-2b** in 1 × HBSS at 37 °C; retention time 11.68 min. **Al[**^**18**^**F]F-2c** in 1 × HBSS at 37 °C; HPLC retention time 11.47 min. **D**. **Al[**^**18**^**F]F-2c-oxide** in 1 × HBSS at 37 °C; retention time 11.47 min. Chromatograms were plotted from exported ASCII data files using Prism 10

The radio-HPLC chromatograms for the radiolabel stability in 1 × HBSS are shown in Fig. 3. **Al[**^**18**^**F]F-2a (**retention time 14.58 min) proved to be highly unstable; after 60 min, only trace amount remained, and two additional radiolabeled species were observed (retention time 5–7 min, Fig. 3A). In contrast, the radio-HPLC analysis for **Al[**^**18**^**F]F-2b** (retention time 11.68 min, Fig. 3B), **Al[**^**18**^**F]F-2c** (retention time 11.47 min, Fig. 3C) and **Al[**^**18**^**F]F-2c-oxide** (retention time 11.47 min, Fig. 3D) showed no evidence of new radiolabeled species under these conditions. Additional radio-HPLC chromatograms for the radiolabel stability of **Al[**^**18**^**F]F-2a** in 2% FBS in 1 × HBSS and **Al[**^**18**^**F]F-2c** in complete medium can be found in the SI (Fig. S5).

The degree of demetallation and/or defluorination for **Al[**^**18**^**F]F-2b**, **Al[**^**18**^**F]F-2c,** and **Al[**^**18**^**F]F-2c-oxide** at 120 min was determined via radio-TLC and is summarized in Table 3 together with the corresponding LogD_pH7.4_ values for all Al[^18^F]F-complexes. Degradation after 120 min amounted to 25 ± 19.3% for **Al[**^**1**^**⁸F]F-2b** (n = 2), ≤ 6% for **Al[**^**1**^**⁸F]F-2c** (n = 3), and 15.2% for **Al[**^**1**^**⁸F]F-2c-oxide** (n = 1). The LogD_pH7.4_ values ranged from -3.95 ± 0.07 to -1.86 ± 0.07, indicating increasing lipophilicity from **Al[**^**1**^**⁸F]F-2a** to **Al[**^**1**^**⁸F]F-2c**. **Al[**^**1**^**⁸F]F-2c-oxide** displayed moderate lipophilicity (LogD_pH7.4_ = -2.03 ± 0.009), comparable to **Al[**^**1**^**⁸F]F-2c**.

**Table 3:** Degree of demetallation and/or defluorination (in %) after 120 min incubation based on radio-TLC analysis and corresponding LogD_pH7.4_ values of the Al[^18^F]F-complexes calculated with the shake-flask method. Values represent mean ± s.d. for experiments performed in replicate (n > 1)

Compound	% of demetallation and/or defluorination	LogD_pH7.4_
Al[^18^F]F-2a	–	− 3.95 ± 0.07 (n = 3)
Al[^18^F]F-2b	25 ± 19.3% (n = 2)	− 2.59 ± 0.04 (n = 2)
Al[^18^F]F-2c	4.2 ± 2.4% (n = 3)	− 1.86 ± 0.07 (n = 4)
Al[^18^F]F-2c-oxide	15.2% (n = 1)	− 2.03 ± 0.009 (n = 3)

Graphs showing the percentage of the intact Al[^18^F]F-complexes over 120 min can be found in the SI (Fig. S6).

#### The bioorthogonal reactions

**Fig. 4:**
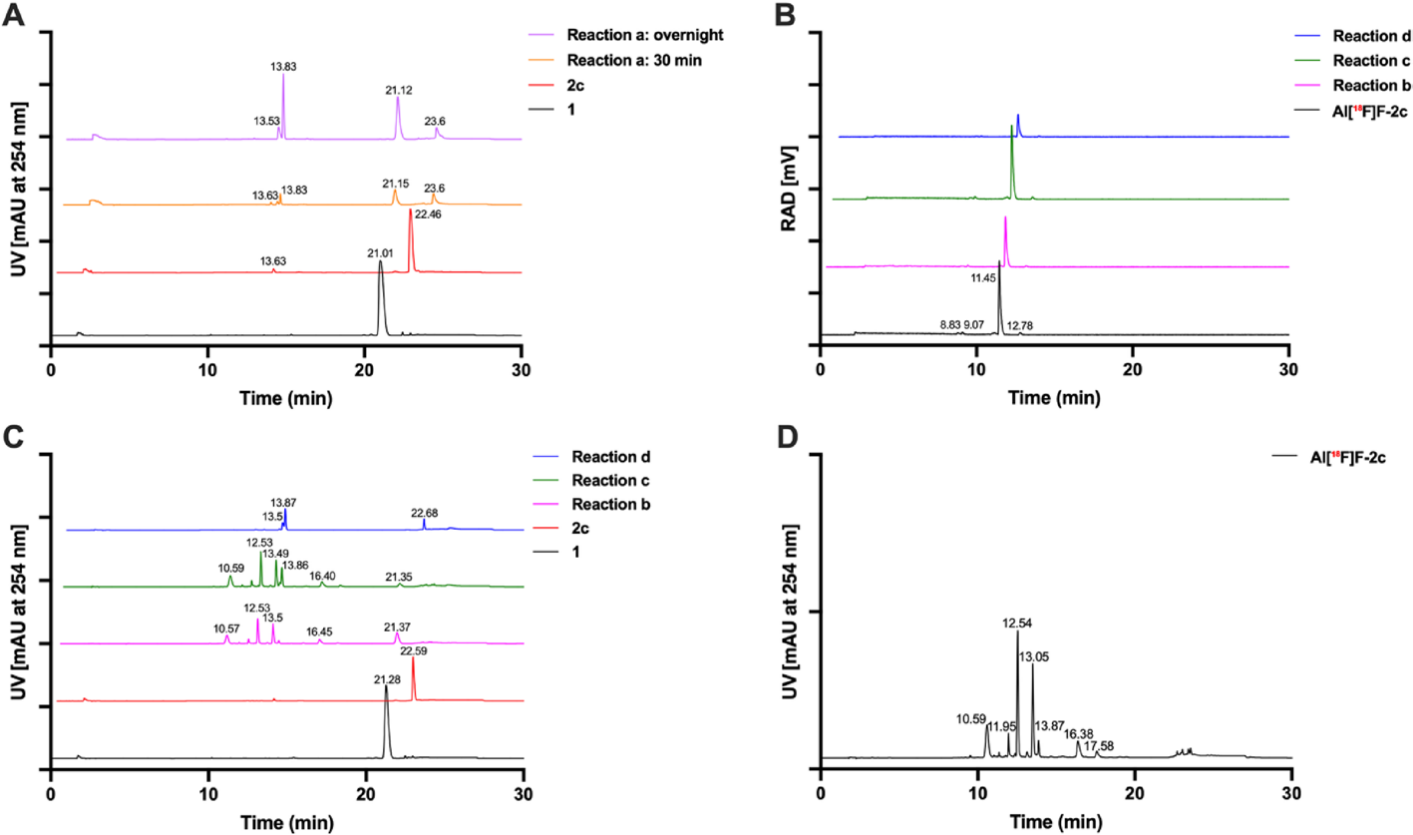
HPLC analyses of the Staudinger ligations (entries **a–d**, described in Scheme 3) between bioorthogonal partners **1** and **2c** or **Al[**^**18**^**F]F-2c**.** A**. UV chromatograms of reference **1** at 21.28 min (black), reference **2c** at 22.59 min (red), the Staudinger ligation of entry **a** after 30 min (orange) and overnight incubation (violet), with Staudinger product at 13.83 min. **B**. Radio-HPLC analysis of the purified **Al[**^**18**^**F]F-2c** (black) and radio signal for the Staudinger ligations **b**, **c**, and **d** after 30 min. **C**. UV chromatograms of reference **1** (black) at 21.28 min and Staudinger ligation **b**, **c**, and **d** at 30 min: multiple species observed. **D.** UV chromatogram of the purified **Al[**^**18**^**F]F-2c** with multiple species formed. Chromatograms were plotted starting from exported ASCII files with Prism 10

HPLC analysis was carried out to monitor the PFAA-Staudinger ligation between **1** and triarylphosphines **2c** or **Al[**^**18**^**F]F-2c**. Figure 4A shows chromatograms of reference compounds **1** and **2c**, alongside reaction mixtures analyzed after 30 min and after overnight incubation (entry **a**, Scheme 3). The **2c** reference presents a minor impurity at 13.63 min (< 5%). Furthermore, the peak at 13.83 min was identified as the Staudinger product using LC–MS (Fig. S7) No new radioactive peak was observed during the reactions between **1** and **Al[**^**18**^**F]F-2c** either without precursor **2c** (entry **b**, Scheme 3) or with added precursor **2c** (entries **c** and **d**, Scheme 3), as shown in Fig. 4B. However, the corresponding UV chromatograms showed multiple species, including peaks at 13.86–13.87 min in reactions **c** and **d**, corresponding to the Staudinger product between **1** and **2c** (Fig. 4C). A more detailed analysis of the UV chromatogram of the **Al[**^**18**^**F]F-2c** showed the presence of additional species, which are annotated with their retention times (Fig. 4D).

#### Comparative HPLC analysis of 2c, 2c-oxide, and their Al^19^F- and Al[^18^F]F-complexes

**Fig. 5:**
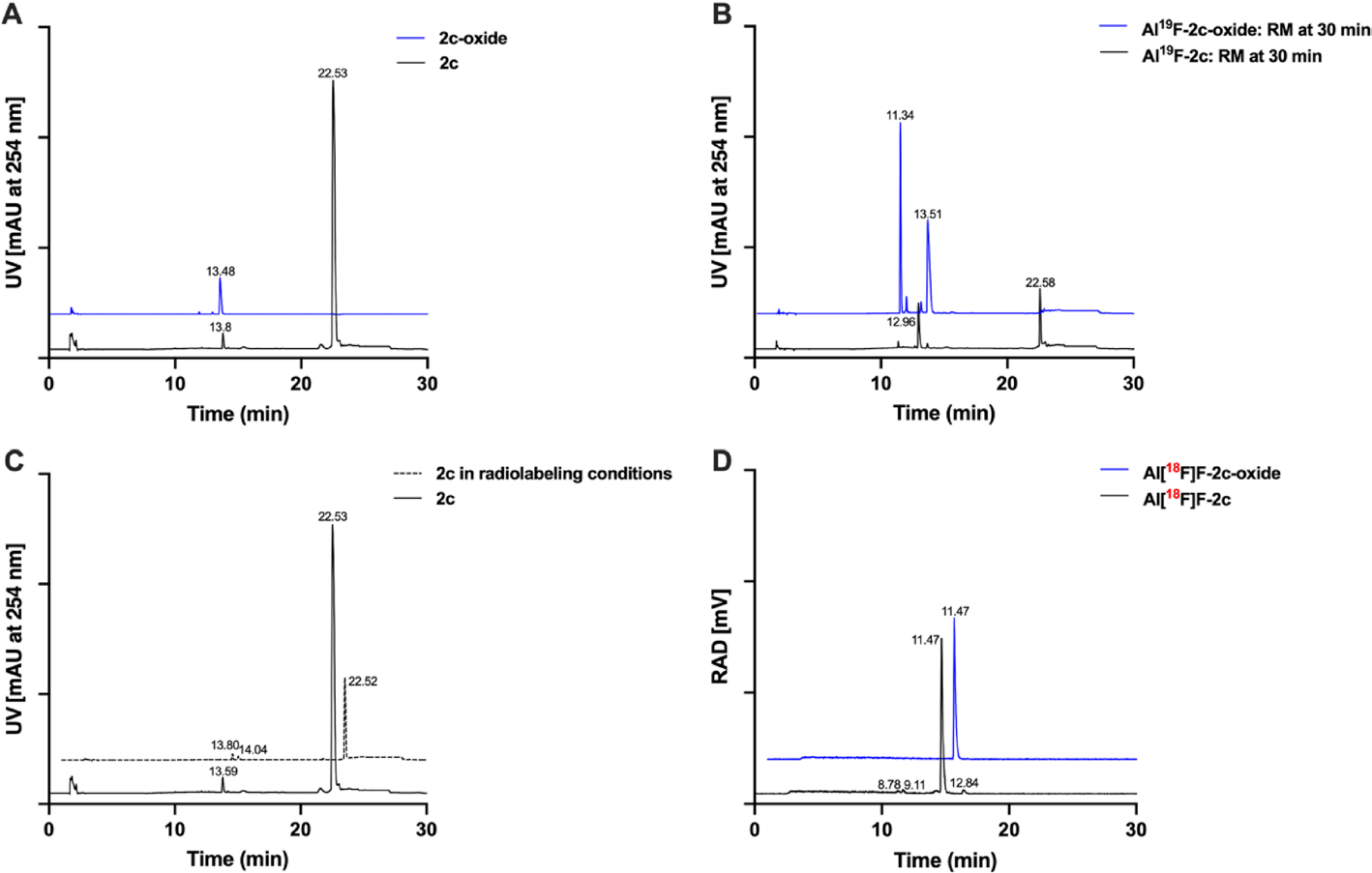
HPLC analyses of **2c**, **2c-oxide** and their Al^19^F- and Al[^18^F]F-complexes. **A**. UV chromatograms of radiolabeling precursors **2c** (black) and **2c-oxide** (blue). **B**. UV chromatograms of the Al^19^F-complex reaction mixtures at 30 min: **Al**^**19**^**F-2c** (black) and **Al**^**19**^**F-2c-oxide** (blue). **C**. UV chromatograms of reference precursor **2c** (black solid line) and precursor **2c** in radiolabeling mixture (black dotted line). **D**. Radio-HPLC chromatograms of **Al[**^**18**^**F]F-2c** (black) and **Al[**^**18**^**F]F-2c-oxide** (blue). Chromatograms were plotted from exported ASCII data files using Prism 10

A pronounced difference in retention time was observed between compound **2c** (22.53 min) and its oxidized form, **2c-oxide** (13.48 min) as shown in Fig. 5A. Al^19^F complexation for both compounds generated new species after 30 min of incubation (Fig. 5B). For **2c**, a peak at 12.96 min was isolated and confirmed as **Al**^**19**^**F-2c** by NMR and HRMS analyses (see SI), whereas **2c-oxide** yielded a new species at 11.34 min, later identified as **Al**^**19**^**F-2c-oxide** by HRMS. Under the same radiolabeling conditions, **2c** showed negligible change after 30 min (Fig. 5C). Notably, the **Al[**^**18**^**F]F**-complexes obtained from **2** and **2c-oxide** showed identical retention times (Fig. 5D). According to radio-HPLC, **Al[**^**18**^**F]F-2c** showed RCP of 90.4%, while **Al[**^**18**^**F]F-2c-oxide** exhibited higher purity (> 99%), with no detectable radioactive side products.

Examples of unprocessed radio-HPLC chromatograms of QC samples for **Al[**^**18**^**F]F-2c** and **Al[**^**18**^**F]F-2c-oxide** are provided in the SI (Fig. S8–S9).

### Cytotoxicity studies

**Fig. 6:**
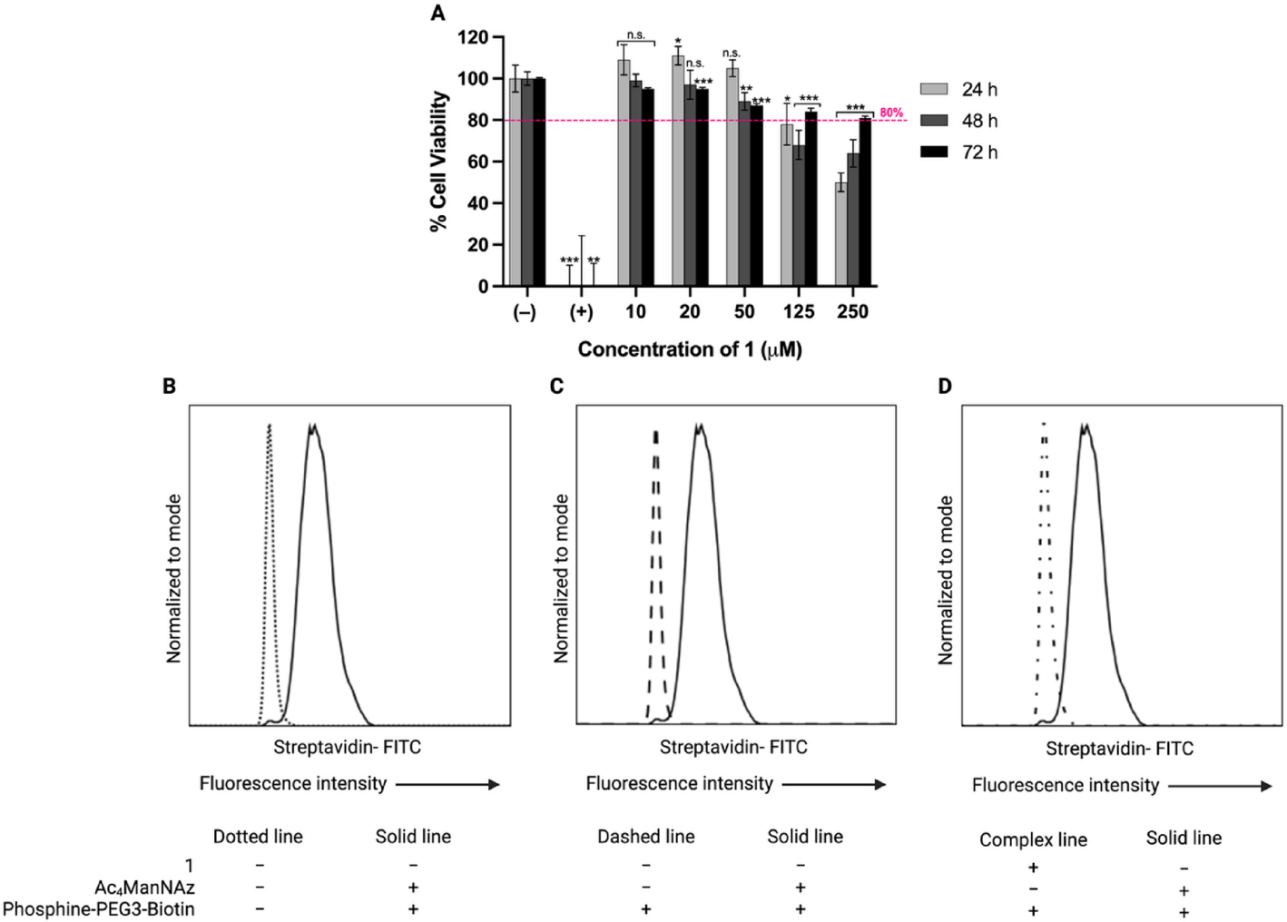
**A**. In vitro cytotoxicity of compound **1** in human Jurkat T lymphoblasts after 24, 48, and 72 h incubation at 10–250 μM. Negative (–) and positive ( +) controls were complete medium with 0.5% v/v DMSO and lysis Buffer (1:10 v/v), respectively. Columns represent mean ± s.d. (n = 4). The statistical significance was analyzed using unpaired Student’s *t*-test versus the negative control (*p < 0.05, **p < 0.01, ***p < 0.001, and n.s. = not significant). The dashed pink line indicates 80% cell viability, the threshold for acceptable cytocompatibility. **B**–**D**. Flow cytometry analysis of live Jurkat cells after a 72 h treatment with 1 × PBS (dotted line in **B** and dashed line in** C**) or MGE with Ac_4_MAnNAz (solid line in **B**–**D**) or **1** (complex line in** D**), followed by incubation with 1 × PBS (dotted line in **B**) or 1 µM phosphine-PEG3-biotin (solid line in **B**–**D** and complex line in **D**) and finally incubation with streptavidin-FITC (all lines)

The cytotoxicity of **1** toward Jurkat cells was evaluated using the LDH release assay after 24, 48, and 72 h of incubation at concentrations ranging from 10 to 250 μM. An 80% cell viability was used as a threshold for acceptable cytocompatibility. Cell viability remained above 80% for concentrations up to 50 μM at all incubation times, indicating negligible cytotoxicity under these conditions. A concentration-dependent decrease in viability was observed at ≥ 125 μM, with cell viability dropping below 80% after 24 and 72 h of incubation. At these higher concentrations, however, viability increased from 24 to 72 h. The control groups behaved as expected, with high LDH release in the positive control and minimal release in the untreated cells. Results are summarized in Fig. 6A.

### Flow cytometry

After 72 h of incubation, cell viability was 97% in controls, 96% with 50 µM of Ac₄ManNAz, and 91% with 50 µM of **1**. At the time of analysis, viability ranged from 84–87% (controls), 79–82% (Ac₄ManNAz), and 77–80% (**1**). Fluorescent labeling with streptavidin-FITC was observed only in cells treated with 50 µM Ac₄ManNAz and phosphine-PEG3-Biotin, confirming the Staudinger ligation. By contrast, control cells and those treated with 1 × PBS or **1** followed by Phosphine-PEG3-Biotin displayed minimal/no signal (Fig. 6B–D), indicating failed MGE with **1**. Additional histograms can be found in the SI (Fig. S21).

## Discussion

Three new triarylphosphines were successfully synthesized. The first derivative, (+)-RESCA-triarylphosphine **2a**, was designed to incorporate a hydrophilic moiety to minimize the inherent lipophilicity of the triarylphosphine—thereby reducing nonspecific membrane interactions—and to favor specific modification through the bioorthogonal reaction with the PFAA. A multistep synthetic strategy enabled us to combine the hydrophilic moiety, the bioorthogonal handle, and the chelator into a single molecule. Although the ester linkage in **2a** raised a concern about hydrolytic stability, we hypothesized that steric hindrance would provide partial protection against decomposition. An amide-linked analogue of **2a** was considered as a potentially more stable alternative but was not pursued because the ester variant was synthetically more accessible. We then focused on developing an Al[^1^⁸F]F radiolabeling protocol for small molecules bearing the RESCA chelator. Notably, chelators tailored for mild Al[^1^⁸F]F radiolabeling have emerged only recently (Cleeren et al. 2016, 2017), in contrast to the long-standing use of macrocyclic chelators such as NOTA (1,4,7-triazacyclononane-1,4,7-triacetic acid) and NODA (1,4,7-triazacyclononane-1,4-diacetic acid). The reported radiolabeling conditions for RESCA with Al[^1^⁸F]F radiolabeling vary across different studies (McBride et al. 2013; Archibald and Allott 2021). At the beginning of this study, only one RESCA-based small molecule had been described (Chen et al. 2022), as the chelator was originally developed for labeling sensitive biomolecules (Cleeren et al. 2018). To date, three additional radiolabeling studies employing RESCA-based small molecules have been reported (Zhang et al. 2025; Iannone et al. 2024; Krutzek et al. 2025). While RESCA was initially developed and used as a racemic mixture (±) in most reported studies, we chose the enantiomerically pure form (+)-RESCA, given its commercial availability and because no in vitro radiolabeling differences relative to the racemate have been reported. Moreover, none of the studies employing (+)-RESCA have conducted a direct comparison with the racemate (Iannone et al. 2024; Krutzek et al. 2025; Lechi et al. 2024). Moreover, our protocol did not require cartridge purification of [^18^F]F^−^ from target water. The highest RCCs were obtained using 0.17 M NaOAc (pH 4.5) as radiolabeling buffer, reaction at ambient temperature for 30 min, a 1:2 Al^3+^:precursor ratio, and a final 1:1 (v/v) 0.17 M NaOAc (pH 4.5):EtOH ratio in the reaction mixture. After optimization, we determined LogD_pH7.4_ of **Al[**^**18**^**F]F**-**2a** and evaluated its stability. The negative LogD_pH7.4_ value (− 3.95 ± 0.07) is consistent with the presence of a hydrophilic moiety and net negative charge characteristic of Al^19^F/Al[^18^F]F-RESCA complexes. (Cleeren et al. 2016, 2017) However, stability studies showed that **Al[**^**18**^**F]F-2a** undergoes rapid decomposition, precluding reliable assessment of demetallation and/or defluorination. Given its pronounced hydrophilicity, we next designed and synthesized **2b**, a simplified analogue bearing only an ethylenediamine linker between the bioorthogonal handle and the (+)-RESCA chelator. Although **2b** was successfully radiolabeled under the optimized conditions, the stability of Al[^18^F]F-complex remained unsatisfactory. We therefore decided to investigate linker effects on complex stability and developed **2c**, incorporating a more rigid cyclohexyl linker. This linker imposes a defined stereochemistry that orients the chelator and the bioorthogonal handle in different directions. The radiolabeling of **2c** achieved high RCCs, and the resulting **Al[**^**18**^**F]F**-**2c** was highly stable with respect to demetallation and/or defluorination. The effect of the precursor structure on Al[^18^F]F-(+)-RESCA stability might not be evident at ambient temperature during chelation but became apparent upon heating in stability assays. In contract, macrocyclic chelators require high temperatures to undergo conformational changes that efficiently accommodate the metal ion. However, once formed, macrocyclic complexes typically exhibit greater kinetic inertness (Price and Orvig 2014). Based on our stability results, we selected **2c** for subsequent in vitro radiolabeling studies. Prior to the radiolabeling trials, we carried out PFAA-Staudinger ligations with all three precursors (**2a**–**2c**), monitored by NMR. All three triarylphosphines **2a**–**2c** react readily with **1** but displayed different reaction kinetics: **2b** followed first-order behavior with rate-limiting phosphazide intermediate decomposition, whereas **2a** and **2c** fit second-order kinetics, indicating rate-limiting phosphazide formation (Lin et al. 2005). While structural differences among the compounds likely contribute to these variations, differing solubility in DMSO-*d₆* may also be a limiting factor and their behavior could vary in other solvent systems. These experiments, however, fell beyond the scope of this study as the end use in vitro would be in predominantly aqueous media. While the kinetic studies provided valuable insights into the reactivity of the newly developed triarylphosphines towards PFAA, the rate constants obtained under these conditions should not be considered absolute. The lack of reactivity of the corresponding **Al[**^**18**^**F]F-2c** complex with **1** prompted further investigation of the complex’s chemical identity formed during radiolabeling. Accordingly, we synthesized the oxidized derivative **2c-oxide** and both reference complexes, **Al**^**19**^**F-2c** and **Al**^**19**^**F-2c-oxide**. **2c-oxide** was also successfully radiolabeled using the optimized protocol. The radio-HPLC QC of **Al[**^**18**^**F]F-2c** showed a main species (90%) with an identical retention time (11.47 min) to **Al[**^**18**^**F]F-2c-oxide**, and only trace amount (~ 3%) of a species at 12.84 min, presumably assigned to **Al[**^**18**^**F]F-2c**. Interestingly, **2c** exhibited little to no oxidation under Al^19^F complexation or simulated labeling conditions without radioactivity, yet oxidized almost completely in the presence of ionizing radiation. Ethanol was chosen as the organic co-solvent, consistent with reports that buffers supplemented with an organic solvent improve RCC in aluminum-[^1^⁸F]fluoride radiolabeling (Archibald and Allott 2021; D’Souza et al. 2011). EtOH is an established antioxidant stabilizer for radiopharmaceuticals (Scott et al. 2009). We therefore hypothesized that at the 1:1 (v/v) level used in the reaction mixture and cartridge eluate, EtOH would partially prevent oxidation of the phosphine. Vugts and co-workers employed antioxidants such as sodium sulfite (Na_2_SO_3_) and a mixture of gentisic acid/tin(II) sulfate (SnSO_4_) during triarylphosphine radiolabeling with ^89^Zr and ^68^Ga, respectively, but their starting activity was much lower: 10–50 MBq ^89^Zr (< 10% oxidation), 10–70 MBq ^67^Ga (< 10% oxidation), 116–120 MBq ^68^Ga (≤ 13% oxidation), 10–50 MBq ^177^Lu (≤ 18% of oxidation) (Vugts et al. 2011). As is well-established, molar activity–and thus starting activity–modulates the degree of radiolysis (Scott et al. 2009). A systematic assessment of antioxidant additives for Al[^1^⁸F]F radiolabeling of these triarylphosphines was beyond the scope of this study and should be addressed in future work.

The LDH assay demonstrated that compound **1** exhibited low cytotoxicity toward Jurkat cells, maintaining viability above the 80% cytocompatibility threshold at concentrations up to 50 µM, even after prolonged exposure. At higher concentrations (125–250 µM), cell viability decreased at earlier time points but partially recovered by 72 h, suggesting transient, adaptive cellular responses. These effects likely reflect a combination of dose-dependent growth inhibition and apoptosis, as previously reported for N-acetylmannosamine (ManNAc) derivatives, with cytotoxicity influenced by factors such as *N*-acyl chain length and cell density (Kim et al. 2004; Almaraz et al. 2012). Overall, these findings justify the use of 50 µM for 72 h as an optimal condition and its selection for subsequent flow cytometry studies, balancing effective exposure with acceptable cell compatibility.

Despite good viability during cell treatments, flow cytometry revealed that Jurkat cells incorporated Ac_4_ManNAz far more efficiently than **1** via MGE. These findings are consistent with earlier studies reporting sugar- and cell line-dependent variability in MGE efficiency (Luchansky et al. 2003). Consistent with our findings, Sundhoro and co-workers reported that A549 cells incorporate tetra-acetylated PFAA-derivatized mannosamine and tetra-acetylated PFAA-derivatized galactosamine to similar extent, but show poor incorporation of the tetra-acetylated PFAA-derivatized glucosamine (Sundhoro et al. 2017). We employed Ac_4_ManNAz as a positive control given its established incorporation on Jurkat cell surface (Saxon and Bertozzi 2000; Saxon et al. 2002), while our primary goal was to leverage the kinetically superior PFAA-Staudinger ligation for cell radiolabeling. Increasing the concentration of **1** to 100 µM with 72 h of incubation reduced cell viability and yielded an inadequate number of cells after the Staudinger ligation with phosphine-PEG3-biotin and the streptavidin-FITC staining, which prevented subsequent flow cytometry analysis. Bertozzi and co-workers demonstrated MGE of Jurkat cells with Ac_4_ManNAz and its use with a modified Staudinger reaction as a bioorthogonal reaction (Saxon and Bertozzi 2000). However, our (+)-RESCA-triarylphosphines were designed for the PFAA-Staudinger ligation; employing a non-traceless Staudinger ligation with Ac_4_ManNAz would cleave the ester trap and release the Al[^18^F]F label from **Al[**^**18**^**F]F-2c**, making that route incompatible with our radiolabeling strategy.

## Conclusions

In this study, we developed and evaluated three novel triarylphosphines designed for PFAA-Staudinger-based radiolabeling. The compounds feature the bioorthogonal azide-reactive moiety, a linker, and the (+)-RESCA chelator, which enables Al[^18^F]F radiolabeling at ambient temperature and contributes to their hydrophilic character. The synthetic strategy allowed efficient access to these multifunctional precursors; however, the ester-containing triarylphosphine **2a** proved unstable under the radiolabeling conditions. To address this, we synthesized (+)-RESCA-triarylphosphines, **2b** and **2c**, with modified linkers to explore how structure influences radiochemical conversion and complex stability. All three compounds underwent the PFAA-Staudinger ligation during the kinetic studies, although reaction kinetics varied. Differences in solubility likely contributed and suggest that behavior may shift under more biologically relevant conditions. Despite successful non-radioactive ligation, radiolabeling revealed significant differences in Al[^18^F]F complex stability: **2a** and **2b** were suboptimal, whereas **2c**—featuring a rigid linker—demonstrated high radiochemical conversion and improved resistance to demetallation and/or defluorination, making it the lead candidate for further studies. Extensive HPLC comparisons of **2c**, its oxidized analogue (**2c-oxide)**, and their AI^19^FA/Al[^18^F]F-complexes revealed substantial oxidation of **2c** during radiolabeling; the main radiolabeled product matched the oxidized species, indicating triarylphosphine oxidation induced by despite ethanol scavenging. In parallel, metabolic incorporation of **1** into Jurkat cell surface was far less efficient than Ac_4_ManNAz, in line with previous reports, and high sugar concentrations reduced viability, limiting downstream applications. Together, these results highlight both the challenges and the limitations of applying Al[^18^F]F-based triarylphosphines to bioorthogonal Staudinger ligations in living cells. Although **2c** emerged as the most promising candidate for the PFAA-Staudinger ligation, issues of radiation-induced oxidation and limited MGE-mediated cell surface modification must be addressed. Nevertheless, this study highlights the potential use of (+)-RESCA for constructing small-molecule radiotracers from sensitive motifs and advance our understanding on the key constrains for application of the PFAA-Staudinger ligation to cell radiolabeling.

## Supplementary Information

Below is the link to the electronic supplementary material.

Supplementary Material 1

## Data Availability

All data analyzed during this study are included in the published article and its Supplementary Information file.

## Abbreviations

7-AAD: 7-Aminoactinomycin D
[^18^F]F⁻: No-carrier-added [^18^F]-fluoride
Ac_4_ManNAz: Tetra-acetylated *N*-azidoacetylmannosamine
ACN: Acetonitrile
Al[^18^F]F: Aluminum [^18^F]fluoride
AlCl_3_: Aluminum chloride
AlCl_3_·6H_2_O: Aluminum chloride hexahydrate
CD_3_CN: Deuterated acetonitrile
CuAAC: Copper-catalyzed azide-alkyne cycloaddition
D_2_O: Deuterium oxide
DC: Dendritic cell
DMF: Dimethylformamide
DMSO-*d*_*6*_: Deuterated dimethyl sulfoxide
DPBS: Dulbecco's phosphate-buffered saline
EtOH: Ethanol
FACS: Fluorescence-activated cell sorting
FBS: Fetal bovine serum
FITC: Fluorescein isothiocyanate
HBSS: Hanks' balanced salt solution
HCOOH: Formic acid
HRMS: High resolution mass spectrometry
HUS: Helsinki University Hospital
LC-MS: Liquid chromatography-mass spectrometry
LDH: Lactate dehydrogenase
logD: Distribution coefficient
ManNAc: *N*-Acetylmannosamine
MGE: Metabolic glycoengineering
Na_2_SO_3_: Sodium sulfite
NaOAc: Sodium acetate
NH_4_OH: Ammonium hydroxide
NMR: Nuclear magnetic resonance
NODA: 1,4,7-Triazacyclononane-1,4-diacetic acid
NOTA: 1,4,7-Triazacyclononane-1,4,7-triacetic acid
PBS: Phosphate-buffered saline
Pd: Palladium
PFAA: Perfluoroaryl azide
PET: Positron emission tomography
PFP: Pentafluorophenyl
QC: Quality control
RCC: Radiochemical conversion
RCP: Radiochemical purity
radio-HPLC: Radio-high performance liquid chromatography
radio-TLC: Radio-thin layer chromatography
RESCA: REstrained Complexing Agent
SnSO_4_: Tin(II) sulfate
SPE: Solid-phase extraction
SPAAC: Strain-promoted alkyne-azide cycloaddition
