# Supplementary material for: Aluminum-[18F]fluoride radiolabeling of triarylphosphines for cell labeling via the perfluoroaryl azide Staudinger ligation
Source: EJNMMI Radiopharm Chem. 2025 Dec 9;10:79. doi: 10.1186/s41181-025-00409-9 (PMC12705485; doi:10.1186/s41181-025-00409-9)
Supplement: Supplementary file 1 — Supplementary Material 1 [file 41181_2025_409_MOESM1_ESM.pdf]

Supporting information

Aluminum-[^{18}F]fluoride radiolabeling of triarylphosphines for cell labeling via the perfluoroaryl azide Staudinger ligation

Anisa Biti[1], Surachet Imlimthan[1], Heidi Harjunpää[2], Diana Barakhtii[1], Topias Pöllänen[1], Arina Sukhova[1], Susanne K. Wiedmer[1], Filip S. Ekholm[1], Susanna Fagerholm[2], Mirkka Sarparanta[1,*]

[1]Department of Chemistry, University of Helsinki, FI-00014 Helsinki, Finland

[2]Molecular and Integrative Biosciences Research Programme, Faculty of Biological and Environmental Sciences, University of Helsinki, FI-00014 Helsinki, Finland

*Corresponding author: Mirkka Sarparanta (mirkka.sarparanta@helsinki.fi)

Table of Contents

Chemistry 2
Kinetic studies on the PFAA-Staudinger ligations of triarylphosphines 2a-2c with 1 by NMR spectroscopy 20
Stability studies 22
LC-MS analysis of the Staudinger product 3c 23
Radiochemistry 23
Example of radio-HPLC chromatogram for Al[^{18}F]F-2c-oxide 25
RadioTLC examples for Al[^{18}F]F-2a 25
RadioTLC examples for Al[^{18}F]F-2b 27
RadioTLC examples for Al[^{18}F]F-2c 28
RadioTLC examples for Al[^{18}F]F-2c-oxide 30
Cell studies 31
NMR spectra 32
HRMS analysis 44
Supplementary references 45

Chemistry

Reagents and solvents

The reagents and solvents were purchased from commercial vendors and used as such without further purification unless otherwise noted. All reagents and solvents were purchased from Sigma-Aldrich (St. Louis, MO, USA), Doug Discovery (Hadfield, UK), TCI (Zwijndrecht, Belgium), BLDPharm (Kaiserslautern, Germany), CheMatech (Dijon, France), Thermo Fisher Scientific (Waltham, MS, USA).

Analysis and purification

Thin layer chromatography (TLC) was conducted using aluminum-backed silica plates with F254 indicator (Sigma Aldrich or Macherey-Nagel), visualized under a UV lamp and/or staining with a potassium permanganate ($KMnO_4$) or ninhydrin staining solution. Compounds were purified by normal-phase flash column chromatography using silica P60 gel with 40-63 μm particle size (230-400 mesh), reversed-phase flash column chromatography using RediSep Gold® C18 Reversed-Phase columns and Teledyne Isco Combiflash Ez Prep (Teledyne Technologies, Thousand Oaks, CA, USA) or semi-preparative high-performance liquid chromatography (see below HPLC method C). A Biotage EV10 Evaporator was used to remove high boiling solvents and an Alpha 1–4 LSC lyophilizer (Martin Christ, Osterode am Harz, Germany) was used to lyophilize samples after reversed-phase flash chromatography. HPLC analysis (method A/B) and purification (method C) was performed using a Shimadzu Prominence (Tokyo, Japan) HPLC system with a DGU-20A degasser, an LC-20AD UPLC LC unit, a SIL-20A HT autosampler, a CTO-20AC column oven, a CBM-20A communications bus module, a SPD-M20A diode array detector (DAD) or ultraviolet (UV) and a Scionix Holland scintillation detector with a 51 BP 51/2 NaI(Tl) crystal for radiodetection. Data was processed with LabSolutions from Shimadzu.

Method A (HPLC and radio-HPLC analysis)

Column: Kinetex 2.6 µm PFP 100 Å, 150 × 2.1 mm, flow rate 1 mL/min

Eluent A: 0.1% trifluoroacetic acid (TFA) in mQ; **Eluent B**: 0.1% TFA in ACN

Gradient: 0–5 min 5% B, 5–15 min 5–40% B, 15–23 min 40 % B, 23–25 min 40–5 % B, 25–30 min 5% B.

Method B (HPLC and radio-HPLC analysis)

Column: Kinetex 2.6 µm PFP 100 Å, 150 × 2.1 mm, flow rate 1 mL/min

Eluent A: 0.1% TFA in mQ; **Eluent B**: 0.1% TFA in ACN

Gradient: 0–5 min 5% B, 5–10 min 5–40% B, 10–20 min 40% B, 20–22 min 40–95 % B, 25–27 min 95–5 % B, 27–30 min 5% B.

Method C (semi-preparative HPLC)

Column HiChrom Alltima 5 µm C18, 250 ×10 mm or Grace Alltima 5µm C18, 250 × 10 mm, flow rate 3 mL/min

Eluent A: 0.1% TFA in mQ; **Eluent B**: 0.1% TFA in ACN

Gradient: 0–5 min 5% B, 5–15 min 5–40% B, 15–20 min 40% B, 20–22 min 40–50 % B, 22-32 min 50 % B, 32–36 min 50–95 % B, 36–40 min 95% B, 40–42 min 95–5% B, 42–45 min 5% B.

LC-MS analysis of reaction mixtures or intermediates was performed using Agilent Technologies 1260 Infinity HPLC-DAD system with Agilent Technologies 6120 Quadruple LC/MS detector; Ionization HESI+, scan range 100-2000 m/z, and an Agilent InfinityLab Poroshell 120EC-C18 (4 mm, 4.6×100 mm) column. Data was processed with OpenLAB CDS Workstation.

Method D (LC-MS method)

Column Poroshell 120EC-C18 (4 mm, 4.6×100 mm), flow rate 1 mL/min

Eluent A: 0.1% FA in mQ; **Eluent B**: 0.1% FA in ACN

Gradient: 0–5 min 5% B, 5–17 min 95% B, 17–20 min 95% B, 20–22 min 5 % B, 22–25 min 5 % B.

HRMS spectra of the final compounds were recorded on an Q Exactive HF Orbitrap mass spectrometer (Exactive series, Thermo Fisher Scientific, Bremen, Germany) with HESI ion source. ^{1}H NMR spectra were acquired on a 400 MHz Bruker Avance NEO (with autosampler) and a 500 MHz Bruker Avance NEO NMR spectrometer with a 5 mm probe. Two-dimensional liquid state NMR spectra were recorded using a 500 MHz Bruker Avance NEO liquid state NMR spectrometer with a 5 mm probe. Spectra were processed using TopSpin 4.0 and MestreNova 14.2.1 software using the non-deuterated residual solvent peaks as references.

Experimental procedures

Tetra-acetylated PFAA-derivatized mannosamine (**1**) was synthesized using a modified reported procedure (**Scheme S1**) [1, 2].

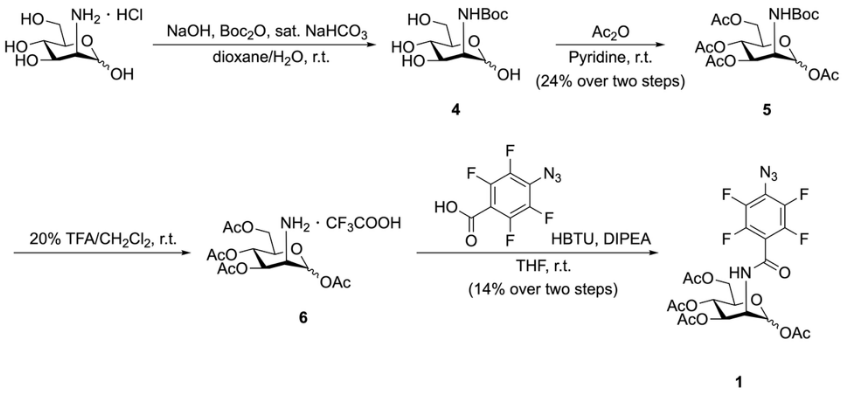

Scheme S1. Synthesis of the tetra-acetylated PFAA-derivatized mannosamine **1** using a modified procedure.

Tetra-acetylated mannosamine intermediate 5

To a solution of D-mannosamine hydrochloride (1 g, 4.64 mmol) in dioxane/H_2O (13.6 mL/3.4 mL) 958.3 µL of 1 M sodium hydroxide (NaOH) and 3.4 mL of saturated sodium bicarbonate ($NaHCO_3$) was added at room temperature. Di-*tert*-butyl dicarbonate (Boc_2O, 1.013 g, 4.64 mmol) was added to the reaction mixture and stirred overnight. The reaction mixture was concentrated (using a Biotage EV-10 Evaporator) to give intermediate **4** as a yellow foam. The residue was used without purification and was directly dissolved in 7 mL of anhydrous pyridine under argon (Ar) atmosphere. Then, 2.2 mL of acetic anhydride (Ac_2O) was added, and the reaction was stirred overnight at room temperature. The reaction mixture was concentrated (Biotage EV-10 Evaporator) and the brown residue was dissolved in dichloromethane (CH_2Cl_2), washed with 1 M hydrochloric acid (HCl), brine and then dried over anhydrous sodium sulfate (Na_2SO_4). The crude product was purified by flash silica gel column chromatography using 30-40% ethyl acetate (EtOAc)/hexane (Hex) (monitored by TLC, stained with $KMnO_4$) to afford 1.083 g of compound **4** as a white foam, in 24% yield over two steps.

^{1}H NMR (400 MHz, $CDCl_3$) δ 6.12 – 5.75 (m, 1H), 5.30 – 4.76 (m, 4H), 4.46 – 3.70 (m, 5H), 2.18 – 1.95 (m, 15H), 1.44 (d, J = 4.0 Hz, 10H);

^{13}C NMR (101 MHz, $CDCl_3$) δ 170.6, 170.6, 170.1, 169.7, 168.5, 168.2, 155.8, 55.1, 92.1, 90.9, 80.5, 80.1, 73.4, 71.5, 70.2, 69.3, 65.6, 65.5, 62.1, 62.0, 50.7, 50.5, 28.3, 28.3, 20.9, 20.9.

Tetra-acetylated PFAA-derivatized mannosamine 1

To a stirred solution of compound **5** (1.083 g, 2.42 mmol) in CH_2Cl_2 (20 mL) at room temperature was added TFA (5 mL). The reaction solution was stirred for 3 hours at room temperature and monitored by TLC using 30% EtOAc/hexane and by staining with $KMnO_4$ and ninhydrin. The solvent was evaporated and the residue was washed several times with

CH_2Cl_2 to afford crude TFA salt **6** which was directly used for the next reaction. Intermediate **6** was dissolved in anhydrous tetrahydrofuran (THF, 20 mL) under Ar atmosphere and O-(Benzotriazol-1-yl)-N,N,N',N'-tetramethyluronium hexafluorophosphate (HBTU, 1.84 g, 4.8 mmol) was added as a solid followed by diisopropylethylamine (DIPEA, 1.27 mL, 7.3 mmol). The reaction mixture was stirred under inert atmosphere for 10 min. 4-azido-2,3,5,6-tetrafluorobenzoic acid (0.683 g, 2.9 mmol) was dissolved in THF (1 mL) under Ar and added to the reaction mixture dropwise. The reaction mixture was stirred overnight. THF was evaporated under vacuum and the residue was dissolved in EtOAc followed by the addition of water. The water phase was extracted three times with EtOAc. The combined organic phases were washed once with brine (saturated aqueous solution of sodium chloride NaCl) and then dried over Na_2SO_4. The crude product was purified using previously deactivated silica (0.1% triethylamine Et_3N in 33% EtOAc/Hex). After the column was packed, the silica was rinsed with 33% EtOAc/Hex to get rid of the residual Et_3N. The crude residue was purified using 33-40% EtOAc/hexane to afford the product **1** as a white solid, 190 mg in 14 % yield over two steps.

^{1}H NMR (400 MHz, $CDCl_3$) δ 6.25 (d, *J* = 9.1 Hz, 1H), 6.15 (d, *J* = 1.9 Hz, 1H), 5.40 (dd, *J* = 10.2, 4.3 Hz, 1H), 5.26 (t, *J* = 10.1 Hz, 1H), 4.83 (ddd, *J* = 9.2, 4.3, 1.9 Hz, 1H), 4.32 – 4.23 (m, 1H), 4.12 – 4.03 (m, 2H), 2.21 (s, 3H), 2.09 (s, 3H), 2.07 (s, 3H), 2.03 (s, 3H);

^{13}C NMR (101 MHz, $CDCl_3$) 13C NMR (101 MHz, CDCl3) δ 170.6, 170.2, 169.5, 168.1, 145.5, 143.1, 141.6, 139.1, 122.5, 110.3, 91.1, 70.1, 68.8, 65.2, 62.0, 49.9, 20.4;

^{19}F NMR (376 MHz, $CDCl_3$) δ -140.25 – -140.46 (m), -150.11 (td, *J* = 13.2, 4.7 Hz);

HRMS (ESI) m/z: Calcd for $C_{21}H_{20}F_4N_4O_{10}$ $[M-H]^-$ 563.1043; Found 563.1044

(+)-RESCA-triarylphosphine 2a

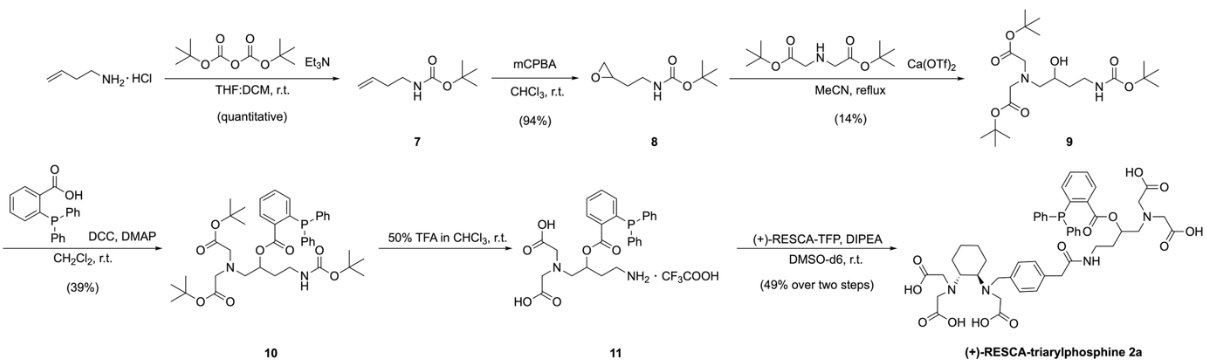

Scheme S2. Synthesis of (+)-RESCA-triarylphosphine **2a.**

Intermediate 7

Compound **7** has already been reported and was synthesized using the same procedure [3]. But-3-en-1-amine hydrochloride (3 g, 28 mmol) and Boc_2O (12.17 g, 56 mmol) were dissolved in THF/CH_2Cl_2 (90 mL/30 mL). Et_3N (15.54 mL, 111.5 mmol) was added, and the reaction mixture was stirred for 24 h at room temperature. The reaction was monitored with TLC (20% EtOAc/hexane and stained with ninhydrin). There was a lot of precipitation. The reaction was quenched with 1 M HCl, until a clear solution was obtained and extracted with EtOAc. The organic layer was washed twice with water and dried over anhydrous Na_2SO_4. The solvent was evaporated under vacuum. The residue was purified by flash silica column chromatography using 0-10% EtOAc/hexane to obtain the product as a colorless oil in quantitative yield (4.70 g).

^{1}H NMR (400 MHz, $CDCl_3$) δ 5.75 (ddt, J = 17.1, 10.2, 6.8 Hz, 1H), 5.17 – 4.99 (m, 2H), 4.55 (br s, 1H), 3.19 (q, J = 6.4 Hz, 2H), 2.23 (qt, J = 6.8, 1.3 Hz, 2H), 1.43 (s, 9H);

^{13}C NMR (101 MHz, $CDCl_3$) δ 156.0, 135.4, 117.1, 85.3, 39.7, 34.3, 28.5.

Epoxide 8

Compound **8** has already been reported and was synthesized using the same procedure [4]. Compound **7** (3 g, 17.5 mmol) was dissolved in anhydrous chloroform ($CHCl_3$, 80 mL) under Ar atmosphere. *m*-Chloroperoxybenzoic acid (*m*-CPBA, 5.89 g, 26.2 mmol, ≤77%) was added as a solid in one portion. The reaction was stirred for 20 h at room temperature and it was monitored with TLC (30 % EtOAc/Hex, followed by staining with $KMnO_4$). The reaction was quenched by adding 180 mL of a 50:50 solution of 10% sodium sulfite (Na_2SO_3) and 10% sodium carbonate (Na_2CO_3) followed by dilution with 90 mL of CH_2Cl_2. After stirring for 1 h, the layers were separated, and the aqueous layer was washed with CH_2Cl_2. The combined organic extracts were combined and dried over anhydrous Na_2SO_4. The solvent was evaporated under vacuum to afford 3.07 g of a low viscosity yellowish oil (crude yield 93.6%). The crude compound was used without further purification.

H NMR (400 MHz, $CDCl_3$) δ 4.78 (br s, 1H), 3.29 (q, *J* = 6.4 Hz, 2H), 2.97 (dtd, *J* = 6.8, 4.1, 2.6 Hz, 1H), 2.77 (t, *J* = 4.0 Hz, 1H), 2.51 (dd, *J* = 4.9, 2.7 Hz, 1H), 1.96 – 1.82 (m, 1H), 1.69 – 1.55 (m, 1H), 1.43 (s, 9H).

Alcohol 9

Compound **9** was synthesized using a reported procedure [5]. Compound **8** (0.93 g, 4.96 mmol) and Di-tert-butyl iminodiacetate (1 g, 4.07 mmol) were dissolved in 14 mL of anhydrous ACN under Ar atmosphere. Calcium trifluoromethanesulfonate ($Ca(OTf)_2$, 0.69 g, 50 mol %) was added as a solid and the reaction mixture was stirred under reflux for 8 h. The color of the reaction mixture changed continuously, from colorless at the beginning to yellow, orange and then red. The reaction was monitored using TLC with 30% EtOAc/hexane or 2% methanol (MeOH)/CH_2Cl_2 followed by staining with $KMnO_4$ and ninhydrin. The reaction mixture was concentrated under vacuum. The residue was dissolved in water and extracted three times with

CH_2Cl_2. The organic phases were combined and dried over anhydrous Na_2SO_4. The crude residue was purified by flash silica column chromatography using 10-30% EtOAc/hexane to obtain 296.4 mg of **9** a low viscosity colorless oil in 14% yield.

^{1}H NMR (400 MHz, $CDCl_3$) δ 5.19 (br s, 1H), 3.58 (ddt, *J* = 10.2, 9.0, 2.9 Hz, 1H), 3.51 – 3.27 (m, 6H), 3.18 (ddt, *J* = 13.3, 7.2, 5.5 Hz, 1H), 2.93 (dd, *J* = 13.4, 2.6 Hz, 1H), 2.35 (dd, *J* = 13.4, 10.4 Hz, 1H), 1.63 – 1.52 (m, 1H), 1.43 (d, *J* = 1.2 Hz, 18H), 1.40 (s, 9H);

^{13}C NMR (101 MHz, $CDCl_3$) δ 171.4, 156.2, 81.6, 67.4, 61.9, 57.1, 38.4, 28.5, 28.5, 28.4, 28.2.

Triarylphosphine ester 10

Compound **10** was synthesized using a reported procedure [6]. Compound **9** (0.244 g, 0.56 mmol) was dissolved in anhydrous CH_2Cl_2 (1.9 mL) under Ar atmosphere to obtain a 0.3 M solution. 2-(Diphenylphosphino)benzoic acid (0.190 g, 0.62 mmol), DCC (0.128 g, 0.62 mmol) and 4-Dimethylaminopyridine (DMAP, 0.055 g, 0.45 mmol) were added as solids and the resulting suspension was stirred at room temperature for 15 h. The reaction was monitored by TLC using 30% EtOAc/hexane and by staining with ninhydrin. Subsequently, the mixture was filtered over celite, washed with CH_2Cl_2, and concentrated under vacuum. The residue was purified by flash silica column chromatography using 10-30% EtOAc/hexane to obtain 157 mg of a white foam, in 39% yield.

^{1}H NMR (400 MHz, $CDCl_3$) δ 8.12 – 8.03 (m, 1H), 7.43 – 7.20 (m, 12H), 6.93 – 6.85 (m, 1H), 5.14 – 4.94 (m, 2H), 3.39 (d, J = 2.8 Hz, 4H), 3.22 – 3.08 (m, 1H), 2.80 (ddd, J = 49.6, 13.9, 6.0 Hz, 2H), 1.91 – 1.77 (m, 1H), 1.68 – 1.51 (m, 2H), 1.48 – 1.36 (m, 27H);

^{13}C NMR (101 MHz, $CDCl_3$) δ 170.6, 166.7, 155.9, 140.3, 140.1, 138.1, 137.9, 137.9, 137.8, 134.2, 134.2, 134.0, 133.9, 133.7, 131.9, 131.0, 128.6, 128.5, 128.4, 128.2, 81.0, 71.8, 57.9, 56.5, 36.6, 32.2, 28.4, 28.1;

^{31}P NMR (162 MHz, $CDCl_3$) δ -4.54.

(+)-RESCA-triarylphosphine 2a

34.9 mg (0.048 mmol) of compound **10** were dissolved in 1.5 mL 50 % TFA in $CHCl_3$ in 4 mL vial and the reaction mixture was stirred overnight while keeping the vial open. ^{31}P NMR and TLC (0.1 % ammonium hydroxide NH_4OH in 10 % MeOH/CH_2Cl_2) were used to monitor the reaction. The residual solvent and TFA were evaporated under vacuum, followed by multiple additions and evaporation of $CHCl_3$ until the crude compound **11** became solid. Compound **11** was used directly for the next reaction without further purification; it was dissolved in 1 mL of DMSO-d_6 followed by addition of DIPEA (59 µL, 0.34 mmol) under Ar atmosphere. The mixture was stirred at room temperature for 10 min, followed by the addition of solid (+)-RESCA-TFP (28.3 mg, 0.048 mmol) in one portion. The reaction mixture was stirred overnight and monitored by HPLC (method A) and ^{31}P NMR. The crude mixture was directly purified by semi-preparative HPLC using method C followed by lyophilization to obtain 22.2 mg of white solid, in 49% yield over two steps.

^{1}H NMR (500 MHz, DMSO) δ 9.03 (s, 1H), 8.03 – 7.99 (m, 1H), 7.66 – 7.55 (m, 2H), 7.55 – 7.46 (m, 3H), 7.37 (tdd, *J* = 5.3, 4.0, 2.7 Hz, 6H), 7.26 (d, *J* = 7.7 Hz, 2H), 7.17 (dddd, *J* = 7.7, 5.3, 3.8, 2.5 Hz, 4H), 6.87 – 6.80 (m, 1H), 4.98 (dq, *J* = 10.7, 5.7 Hz, 1H), 4.26 (s, 2H), 3.85 (d, *J* = 17.4 Hz, 2H), 3.54 – 3.30 (m, 10H), 3.03 (q, *J* = 7.3 Hz, 2H), 2.95 (dt, *J* = 13.3, 6.7 Hz, 2H), 2.75 (d, *J* = 5.9 Hz, 2H), 2.20 (d, *J* = 11.3 Hz, 1H), 2.07 (d, *J* = 5.9 Hz, 1H), 1.75 (d, *J* = 7.9 Hz, 2H), 1.69 (s, 1H), 1.56 (h, *J* = 7.5 Hz, 1H), 1.42 (s, 1H), 1.25 (d, *J* = 9.9 Hz, 4H);

^{13}C NMR (126 MHz, DMSO) δ 172.7, 170.0, 166.3, 139.4, 139.2, 138.0, 138.0, 137.9, 135.3, 135.1, 134.2, 133.9, 133.9, 133.8, 133.8, 132.5, 130.9, 129.8, 129.2, 129.1, 129.1, 72.2, 57.3, 55.3, 42.4, 35.7, 32.0, 24.3, 24.1;

^{31}P NMR (162 MHz, DMSO) δ -6.43;

HRMS (ESI) m/z: Calcd for $C_{48}H_{54}N_4O_{13}P$ $[M-H]^-$ 925.3430; Found 925.3427

Synthesis of (+)-RESCA-triarylphosphine 2b

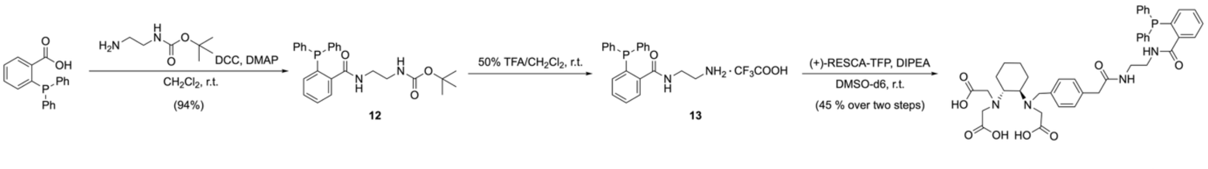

Scheme S3. Synthesis of (+)-RESCA-triarylphosphine **2b**

Triarylphosphine 12

Compound **12** was synthesized using a reported procedure. Tert-butyl (2-aminoethyl)carbamate (0.2 g, 1.25 mmol) was dissolved in anhydrous CH_2Cl_2 (4.2 mL) under Ar atmosphere. 2-(Diphenylphosphino)benzoic acid (0.420 g, 1.37 mmol), N,N′-dicyclohexylcarbodiimide (DCC, 0.283 g, 1.37 mmol) and DMAP (0.122 g, 0.1 mmol) were added as solids and the resulting suspension was stirred at room temperature for 15 h. The reaction was monitored by TLC using 70% EtOAc/hexane and by staining with ninhydrin. Subsequently, the mixture was filtered over celite, washed with CH_2Cl_2, and concentrated under vacuum. The residue was purified by flash silica column chromatography using 30-50% EtOAc/hexane to obtain 0.526 g of a white foam, in 94 % yield.

^{1}H NMR (400 MHz, $CDCl_3$) δ 7.59 (m, 1H), 7.39 – 7.24 (m, 12H), 6.95 (m, 1H), 6.48 (br s, 1H), 4.96 (br s, 1H), 3.36 (q, J = 5.6 Hz, 2H), 3.19 (q, J = 5.8 Hz, 2H), 1.43 (s, 9H);

^{13}C NMR (101 MHz, $CDCl_3$) δ 169.5, 156.6, 141.3, 141.0, 137.1, 137.0, 136.1, 135.9, 134.2, 134.0, 133.8, 130.3, 129.0, 128.9, 128.8, 128.7, 128.7, 127.8, 127.8, 79.6, 60.5, 49.1, 40.9, 40.2, 34.0, 28.5;

^{31}P NMR (162 MHz, $CDCl_3$) δ -9.93.

(+)-RESCA-triarylphosphine 2b

Compound **12** (22.4 mg, 0.05 mmol) was dissolved in 1 mL 50% TFA/CH_2Cl_2 and the reaction mixture was stirred for 1 h at room temperature. The reaction was monitored by TLC using 70% EtOAc/hexane and by staining with ninhydrin. The residual solvent and TFA were evaporated under vacuum, followed by multiple rounds of addition and evaporation of $CHCl_3$ until the crude compound **13** became solid. Then after, intermediate **12** was directly dissolved in 1 mL DMSO-d6 under Ar atmosphere followed by the addition of DIPEA (60.9 μL, 0.35 mmol). The mixture was stirred at room temperature for 10 min, followed by the addition of solid (+)-RESCA-TFP (35 mg, 0.06 mmol) in one portion. The reaction mixture was stirred for 3 h at room temperature and monitored by HPLC (method A) and ^{31}P NMR. The crude mixture was directly purified by semi-preparative HPLC (method C) followed by lyophilization to obtain 22.1 mg of white solid, in 58% yield over two steps.

^{1}H NMR (500 MHz, DMSO) δ 12.71 (s, 1H), 8.42 (t, *J* = 5.6 Hz, 1H), 8.11 (t, *J* = 5.6 Hz, 1H), 7.62 – 7.53 (m, 3H), 7.46 – 7.34 (m, 8H), 7.27 (dd, *J* = 8.0, 4.3 Hz, 2H), 7.20 – 7.14 (m, 4H), 4.43 (s, 2H), 4.26 (s, 2H), 3.86 (d, *J* = 16.3 Hz, 2H), 3.44 (s, 3H), 3.27 (s, 2H), 3.15 (dt, *J* = 23.3, 6.2 Hz, 5H), 2.21 (d, *J* = 11.6 Hz, 1H), 2.08 (s, 1H), 1.75 (d, *J* = 6.9 Hz, 1H), 1.69 (s, 1H), 1.25 (d, *J* = 10.2 Hz, 4H);

^{13}C NMR (126 MHz, DMSO) δ 170.5, 168.7, 158.6, 158.3, 142.0, 141.8, 138.6, 138.5, 136.9, 136.7, 134.2, 133.8, 133.6, 130.3, 129.9, 129.0, 128.9, 128.8, 127.8, 127.8, 79.6, 42.5, 24.3, 24.1;

^{31}P NMR (202 MHz, DMSO) δ -9.97;

HRMS (ESI) m/z: Calcd for $C_{42}H_{46}N_4O_8P$ $[M-H]^-$ 765.3059; Found 765.3055.

Synthesis of (+)-RESCA-triayphosphine 2c

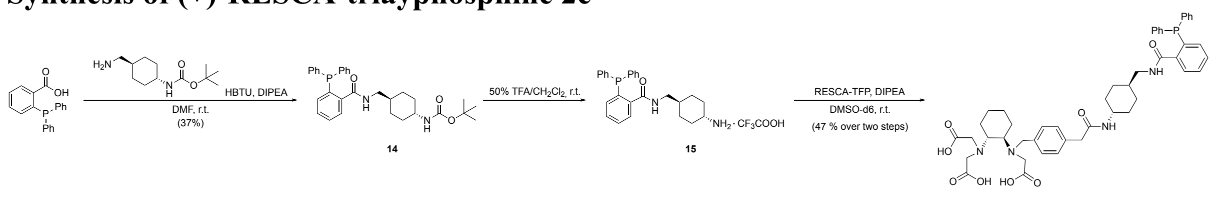

Scheme S4. Synthesis of (+)-RESCA-triarylphosphine **2c.**

Triarylphosphine 14

Compound **14** was synthesized using a reported procedure [7]. HBTU (0.0302 g, 0.0796 mmol) was dissolved in 1 mL anhydrous DMF under Ar atmosphere. DIPEA (139 µL 0.8 mmol) and 2-(Diphenylphosphino)benzoic acid (0.0244 g, 0.0796 mmol) were added. The mixture was stirred for 10 min and a solution of tert-butyl ((1*r*,4*r*)-4-(aminomethyl)cyclohexyl)carbamate (0.020 g, 0,0876 mmol) in 1 mL anhydrous DMF was added. The reaction mixture was stirred a room temperature overnight and monitored by TLC using 50% EtOAc/Hex and staining with ninhydrin. The mixture was further diluted with EtOAc and washed three times with saturated sodium bicarbonate ($NaHCO_3$) and once with 0.1 M HCl. The organic phase was dried over anhydrous Na_2SO_4, and the solvent was evaporated under vacuum. The residue was purified by flash silica column chromatography using 10-50% EtOAc/hexane to obtain 15.3 mg of a white solid in 37% yield.

^{1}H NMR (400 MHz, $CDCl_3$) δ 7.61 (ddd, *J* = 7.6, 3.8, 1.4 Hz, 1H), 7.42 – 7.21 (m, 12H), 6.93 (ddd, *J* = 7.7, 4.1, 1.3 Hz, 1H), 6.01 (br s, 1H), 4.34 (br s, 1H), 3.30 (br s, 1H), 3.11 (t, *J* = 6.4 Hz, 2H), 1.94 (dd, *J* = 9.4, 4.9 Hz, 2H), 1.63 (dd, 2H), 1.41 (s, 9H), 1.37 – 1.22 (m, 2H), 1.08 – 0.78 (m, 2H);

^{13}C NMR (101 MHz, $CDCl_3$) δ 169.1, 155.3, 142.0, 141.8, 136.9, 136.8, 135.3, 135.1, 134.2, 134.0, 133.8, 130.2, 129.0, 129.0, 128.9, 128.8, 128.7, 128.3, 128.2, 79.2, 60.4, 53.4, 45.8, 36.9, 33.0, 29.5, 28.5;

^{31}P NMR (162 MHz, $CDCl_3$) δ -10.48.

(+)-RESCA-triarylphosphine 2c

Compound **14** (26.5 mg, 0.05 mmol) was dissolved in 0.8 mL 50% TFA in CH_2Cl_2 and the reaction mixture was stirred for 1 h at room temperature. The reaction was monitored by TLC using 50% EtOAc/hexane and by staining with ninhydrin. The residual solvent and TFA were evaporated under vacuum, followed by multiple additions and evaporation of $CHCl_3$ until the crude compound **15** became solid. Then after, intermediate **15** was directly dissolved in 1 mL deuterated dimethyl sulfoxide (DMSO-*d6*) under Ar atmosphere followed by the addition of DIPEA (62.6 µL, 0.36 mmol). The mixture was stirred at room temperature for 10 min, followed by the addition of solid (+)-RESCA-TFP (30 mg, 0.05 mmol) in one portion. The reaction mixture was stirred for 3 h at room temperature and monitored by HPLC (method A) and ^{31}P NMR. The crude mixture was directly purified by semi-preparative HPLC (method C) followed by lyophilization to obtain 17.2 mg of white solid, in 42% yield over two steps.

^{1}H NMR (500 MHz, DMSO-d_6) δ 12.64 (s, 3H), 8.36 (t, *J* = 5.9 Hz, 1H), 7.95 (d, *J* = 7.9 Hz, 1H), 7.56 (ddd, *J* = 7.6, 3.9, 1.4 Hz, 1H), 7.54 – 7.38 (m, 4H), 7.36 (dh, *J* = 5.2, 1.9 Hz, 6H), 7.24 (d, *J* = 7.8 Hz, 2H), 7.21 – 7.12 (m, 4H), 6.89 (ddd, *J* = 7.7, 3.9, 1.3 Hz, 1H), 4.33 (d, *J* = 12.6 Hz, 1H), 4.04 (s, 1H), 3.63 (s, 1H), 3.45 (dp, *J* = 11.3, 3.6 Hz, 1H), 3.38 (s, 2H), 3.17 (s, 1H), 3.06 (s, 1H), 2.96 (t, *J* = 6.3 Hz, 2H), 2.16 (d, *J* = 11.6 Hz, 1H), 2.05 (s, 2H), 1.77 (d, *J* = 12.8 Hz, 2H), 1.71 (d, *J* = 15.8 Hz, 4H), 1.37 (s, 2H), 1.29 – 1.19 (m, 4H), 1.11 (q, *J* = 12.5 Hz, 2H), 0.97 – 0.87 (m, 2H);

^{13}C NMR (126 MHz, DMSO) δ 173.1, 169.2, 168.6, 142.6, 142.4, 138.6, 138.5, 136.5, 136.4, 134.2, 133.8, 133.6, 131.6, 130.1, 129.7, 129.0, 128.8, 127.8, 65.1, 60.2, 57.8, 55.2, 49.3, 48.4, 45.4, 42.5, 37.1, 32.4, 29.6, 25.9, 24.4, 24.1;

^{31}P NMR (162 MHz, DMSO) δ -10.33;

HRMS (ESI) m/z: Calcd for $C_{47}H_{55}N_4O_8P$ $[M-H]^-$ 833.3685; Found 833.3696.

Synthesis of (+)-RESCA-triarylphosphine oxide 2c-oxide

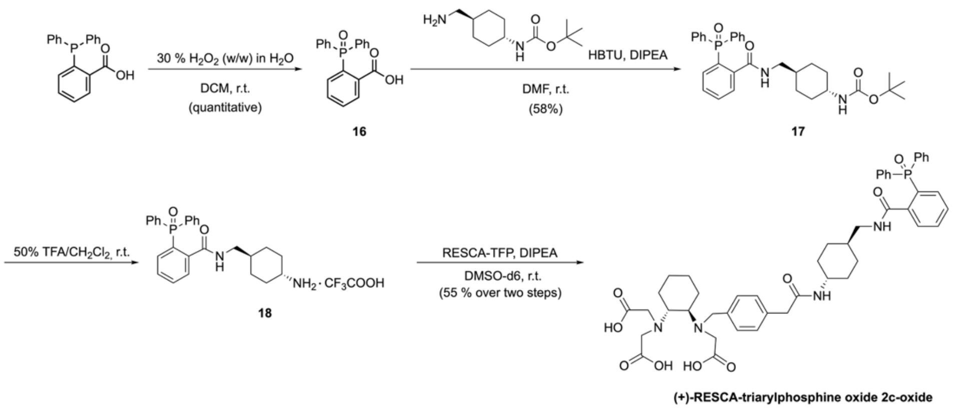

Scheme S5. Synthesis of (+)-RESCA-triarylphosphine oxide **2c-oxide**.

Triarylphosphine oxide 16

Compound **16** has already been reported and was synthesized using the same procedure [8]. To the suspension of 2-(diphenylphosphino)benzoic acid (0.1 g, 0.33 mmol) in 1 mL anhydrous CH_2Cl_2 was added hydrogen peroxide (H_2O_2) 30% (w/w) in H_2O (66 µL, 0.65 mmol) and the suspension turned into a clear transparent solution. After 5 min of stirring at room temperature, the reaction mixture became a white milky suspension. The reaction was monitored by ^{31}P NMR and TLC using 50% EtOAc/hexane. The reaction mixture was diluted with water and CH_2Cl_2 and the compound was extracted with CH_2Cl_2 three times. The combined organic phases were washed twice with water, dried over anhydrous Na_2SO_4, and concentrated under vacuum. The white solid was obtained in quantitative yield (105 mg) and was used without further purification.

^1H NMR (400 MHz, $CDCl_3$) δ 8.35 (ddd, *J* = 7.9, 4.2, 1.3 Hz, 1H), 7.68 (tt, *J* = 7.7, 1.5 Hz, 1H), 7.65 – 7.56 (m, 6H), 7.55 – 7.43 (m, 5H), 7.08 (ddd, *J* = 15.0, 7.7, 1.3 Hz, 1H);

^{13}C NMR (101 MHz, $CDCl_3$) δ 167.5, 137.9, 137.9, 134.3, 134.2, 133.8, 133.7, 132.9, 132.8, 132.8, 132.7, 132.3, 132.2, 130.9, 130.8, 130.8, 130.5, 129.7, 129.5, 129.0, 128.9;

^{31}P NMR (162 MHz, $CDCl_3$) δ 40.60.

Triarylphosphine oxide 17

Compound **17** was synthesized using a reported procedure [7]. HBTU (83 mg, 0.22 mmol) was dissolved in 1 mL anhydrous DMF under Ar atmosphere. DIPEA (60 µL 0.4 mmol) and compound **16** (54.5 mg, 0.17 mmol) were added. The mixture was stirred for 10 min and a solution of tert-butyl ((1r,4r)-4-(aminomethyl)cyclohexyl)carbamate (50 mg, 0.22 mmol) in 0.5 mL anhydrous DMF was added. The reaction mixture was stirred at room temperature overnight and monitored by TLC using 50% EtOAc/Hex and staining with ninhydrin. The crude compound was purified using flash reverse phase column chromatography (5.5 g Redisep Gold® C18 Reversed Phase Columns) to obtain 61.5 mg of white solid, in 58% yield.

^{1}H NMR (400 MHz, $CDCl_3$) δ 8.73 (br s, 1H), 8.00 (ddd, *J* = 7.8, 4.1, 1.3 Hz, 1H), 7.72 – 7.43 (m, 11H), 7.36 (tt, *J* = 7.6, 1.7 Hz, 1H), 7.04 (ddd, *J* = 14.5, 7.8, 1.3 Hz, 1H), 4.33 (br s, 1H), 3.30 (br s, 1H), 2.74 (t, *J* = 6.2 Hz, 2H), 1.93 (dd, *J* = 8.4, 4.4 Hz, 2H), 1.81 – 1.62 (m, 2H), 1.42 (s, 9H), 1.38 – 1.28 (m, 1H), 1.08 – 0.86 (m, 4H);

^{13}C NMR (101 MHz, $CDCl_3$) δ 167.6, 167.5, 155.3, 141.4, 141.4, 133.5, 133.3, 132.8, 132.8, 132.5, 132.5, 132.0, 131.9, 131.8, 131.7, 131.7, 130.6, 129.9, 129.8, 129.5, 128.9, 128.8, 128.5, 49.7, 45.9, 36.7, 33.1, 29.7, 28.6;

^{31}P NMR (162 MHz, $CDCl_3$) δ 35.54.

(+)-RESCA-triarylphosphine oxide 2c-oxide

Compound **16** (20 mg, 0.04 mmol) was dissolved in 1 mL 50:50 TFA/CH_2Cl_2 and the reaction mixture was stirred for 1 h at room temperature. The reaction was monitored by TLC using 70 % EtOAc/hexane and by staining with ninhydrin. The residual solvent and TFA were evaporated under vacuum, followed by multiple rounds of addition and evaporation of $CHCl_3$ until the crude compound **17** started to solidify. Then after, intermediate **17** was directly

dissolved 1 in 1 mL DMSO-d6 under Ar atmosphere followed by the addition of DIPEA (45.7 μL, 0.26 mmol). The mixture was stirred at room temperature for 10 min, followed by the addition of solid (+)-RESCA-TFP (24 mg, 0.04 mmol) in one portion. The reaction mixture was stirred for 3 h at room temperature and monitored by HPLC (method A) and ^{31}P NMR. The crude mixture was directly purified by reversed-phase flash column chromatography using RediSep Gold® C18 Reversed-Phase columns with Teledyne Isco Combiflash Ez Prep followed by lyophilization to obtain 17.4 mg of white solid, in 55% yield over two steps.

^{1}H NMR (400 MHz, DMSO) δ 8.44 (t, *J* = 5.6 Hz, 1H), 7.92 (d, *J* = 7.8 Hz, 1H), 7.70 (tt, *J* = 7.3, 1.5 Hz, 1H), 7.66 – 7.47 (m, 13H), 7.39 (d, *J* = 7.9 Hz, 2H), 7.18 (d, *J* = 7.9 Hz, 2H), 4.08 (d, *J* = 12.7 Hz, 1H), 3.73 (d, *J* = 12.8 Hz, 1H), 3.42 (t, *J* = 3.8 Hz, 1H), 3.38 – 3.31 (m, 6H), 3.13 (d, *J* = 16.6 Hz, 1H), 2.58 (t, *J* = 6.1 Hz, 2H), 2.00 (t, *J* = 12.3 Hz, 1H), 1.80 – 1.56 (m, 6H), 1.33 – 1.01 (m, 8H), 0.92 – 0.78 (m, 2H);

^{13}C NMR (101 MHz, DMSO) δ 172.1, 170.7, 169.4, 167.6, 167.6, 141.3, 141.2, 136.6, 134.0, 133.7, 132.6, 132.5, 132.1, 132.1, 132.0, 131.1, 130.2, 130.0, 129.9, 129.7, 129.7, 129.2, 128.8, 128.7, 61.9, 61.5, 52.6, 48.3, 45.4, 42.6, 36.7, 32.3, 29.6, 26.0, 24.9, 24.7;

^{31}P NMR (162 MHz, DMSO) δ 30.02;

HRMS (ESI) m/z: Calcd for $C_{47}H_{55}N_4O_9P$ $[M-H]^-$ 849.3634; Found 849.3640.

Synthesis of Al^{19}F-2c

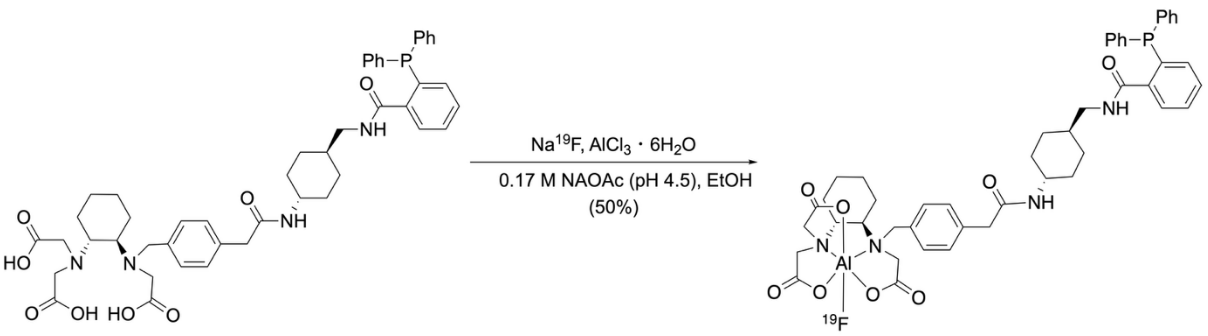

2c **Al^{19}F-2c**

Scheme S6. Synthesis of **Al^{19}F-2c**.

A solution of 5 equivalents of $AlCl_3$ (61 µmol, 610 µL of a 100 mM solution in 0.1 M NaOAc pH 4.5) and 5 equivalents of NaF (61 µmol, 610 µL of a 100 mM solution in 0.1 M NaOAc pH 4.5) was stirred at room temperature for 10 min. 10.2 mg of (+)-RESCA-triarylphosphine **2c** (1 eq., 12.2 µmol) dissolved in 1220 µL absolute EtOH was added to the mixture to achieve a 50:50 to achieve a final 1:1 (v/v) aqueous to organic solvent ratio. The reaction mixture was stirred at room temperature for 2 h and monitored by HPLC (method B). The compound was purified using semi-preparative HPLC (method C) to obtain 5.4 mg, 50% yield.

^{1}H NMR (500 MHz, DMSO) δ 8.44 (t, J = 5.8 Hz, 1H), 7.93 (d, J = 7.8 Hz, 1H), 7.69 (t, J = 7.5 Hz, 1H), 7.66 – 7.47 (m, 13H), 7.39 (d, J = 7.7 Hz, 2H), 7.18 (d, J = 7.8 Hz, 2H), 4.09 (d, J = 12.7 Hz, 1H), 3.73 (d, J = 12.7 Hz, 1H), 3.42 – 3.40 (m, 1H), 3.36 – 3.32 (m, 6H), 3.13 (d, J = 16.5 Hz, 1H), 2.58 (t, J = 6.2 Hz, 2H), 2.01 (d, J = 21.7 Hz, 1H), 1.74 (d, J = 12.1 Hz, 2H), 1.67 (s, 1H), 1.61 (d, J = 13.4 Hz, 3H), 1.26 (d, J = 17.3 Hz, 2H), 1.15 (s, 3H), 1.07 (t, J = 12.4 Hz, 3H);

^{13}C NMR (126 MHz, DMSO) δ 172.1, 170.7, 169.4, 167.6, 141.3, 141.3, 136.6, 133.9, 133.6, 132.7, 132.5, 132.1, 132.0, 131.0, 130.2, 130.0, 129.9, 129.7, 129.7, 129.2, 128.8, 128.7, 61.9, 61.5, 54.5, 52.6, 51.5, 48.2, 45.4, 42.6, 36.7, 32.3, 29.6, 26.0, 24.9, 24.6;

^{31}P NMR (162 MHz, DMSO) δ -10.34;

^{19}F NMR (376 MHz, DMSO) δ -173.66;

HRMS (ESI) m/z: Calcd for $C_{47}H_{52}AlFN_4O_8P$ $[M]^-$ 877.3328; Found 877.3345.

Synthesis of Al^{19}F-2c-oxide

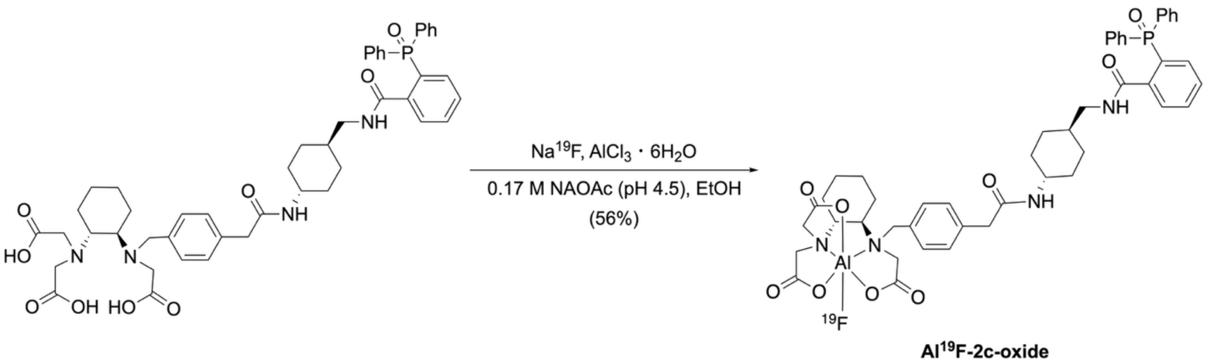

Scheme S7. Synthesis of **Al^{19}F-2c-oxide**

A solution of 5 equivalents of $AlCl_3$ (7 µmol, 70 µL of a 100 mM solution in 0.1 M NaOAc pH 4.5) and 5 equivalents of NaF (7 µmol, 70 µL of a 100 mM solution in 0.1 M NaOAc pH 4.5) and 60 µL of 0.1 M NaOAc pH 4.5 was stirred at room temperature for 10 min. 1.2 mg of **2c-oxide** (1 eq., 1.4 µmol) dissolved in 200 µL absolute EtOH was added to the mixture to achieve a 50:50 to achieve a final 1:1 (v/v) aqueous to organic solvent ratio. The reaction mixture was stirred at room temperature for 30 min and monitored by HPLC using method B. The crude mixture was purified using Sep-Pak Alumina N Plus Light Cartridge and eluting with 1:1 (v:v) mQ:EtOH to obtain 0.7 mg of white solid, in 56% yield.

HRMS (ESI) m/z: Calcd. for $C_{47}H_{52}AlFN_4O_9P$ $[M]^-$ 893.3277; Found 893.3276.

Kinetic studies on the PFAA-Staudinger ligations of triarylphosphines 2a-2c with 1 by NMR spectroscopy

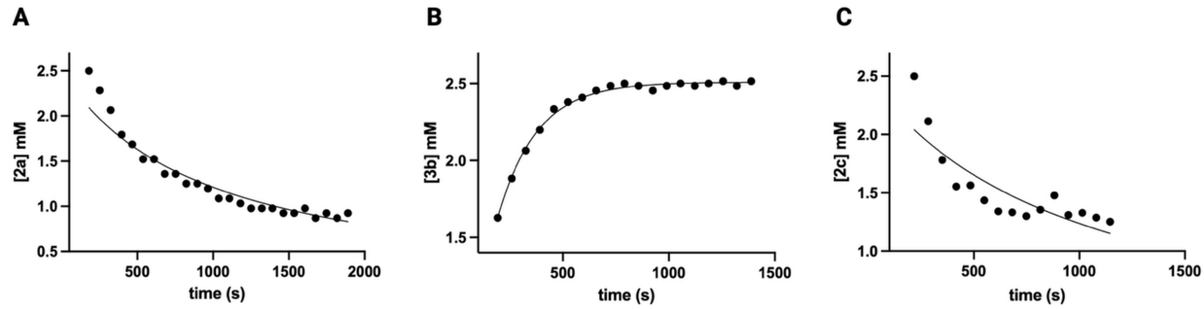

Fig. S1. Kinetic studies of the Staudinger reactions between **1** and **2a** (**A**), **2b** (**B**), and **2c** (**C**), respectively. Reactions of **2a** and **2c** proceeded with second-order kinetics (**A** & **C**) while the reaction of **2b** proceeded with a first-order kinetics (**B**). Non-linear fitting was done using Prism 10 and the equations for first- and second- order kinetics.

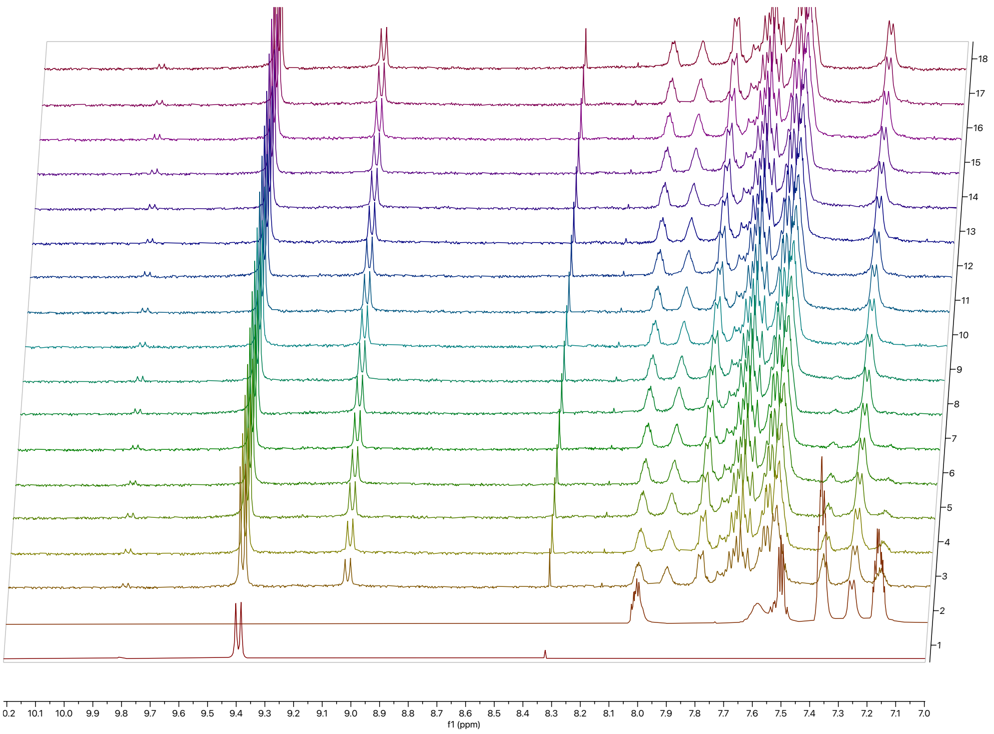

Fig. S2. Stacked ^{1}H NMR spectra showing the progression of the Staudinger reaction between **1** and **2a** in DMSO-d6. Spectrum no. **1** (y-axis) corresponds to the ^{1}H NMR spectrum of compound **1** and spectrum no. **2** corresponds to the ^{1}H NMR spectrum of compound **2a**.

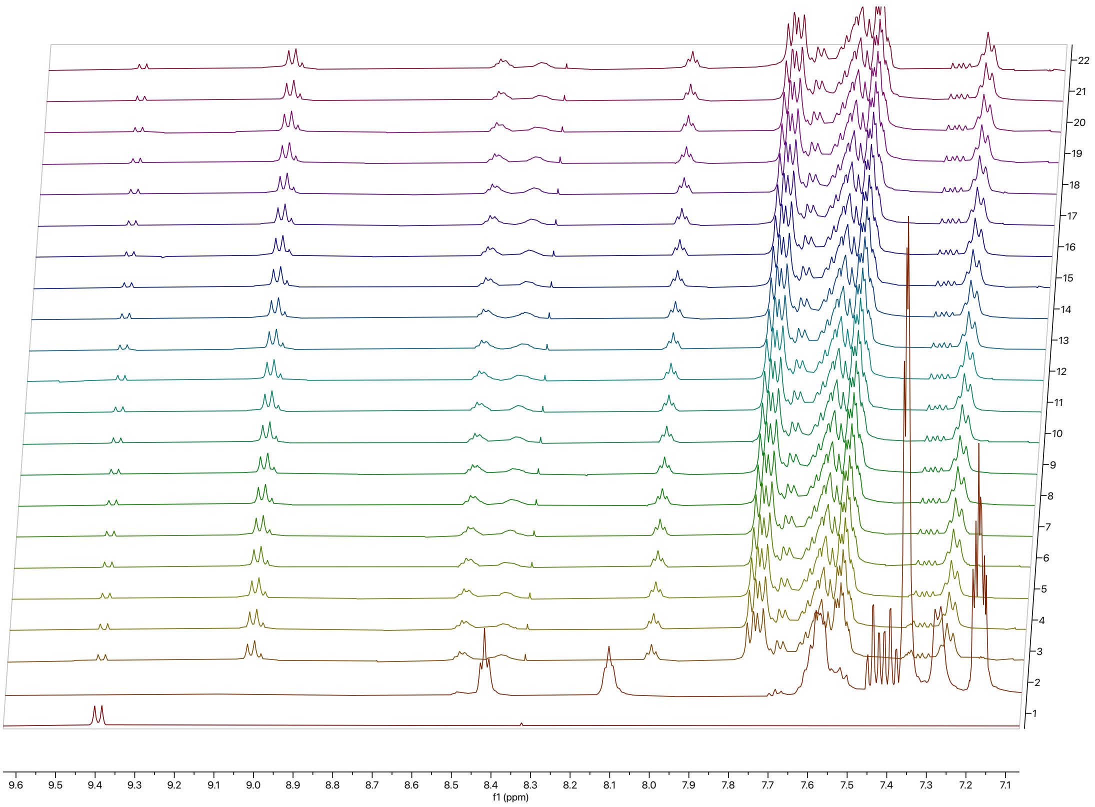

Fig. S3. Stacked ^{1}H NMR spectra showing the progression of the Staudinger reaction between **1** and **2b** in DMSO-d6. Spectrum no. **1** (y-axis) corresponds to the ^{1}H NMR spectrum of compound **1** and spectrum no. **2** corresponds to the ^{1}H NMR spectrum of compound **2b**.

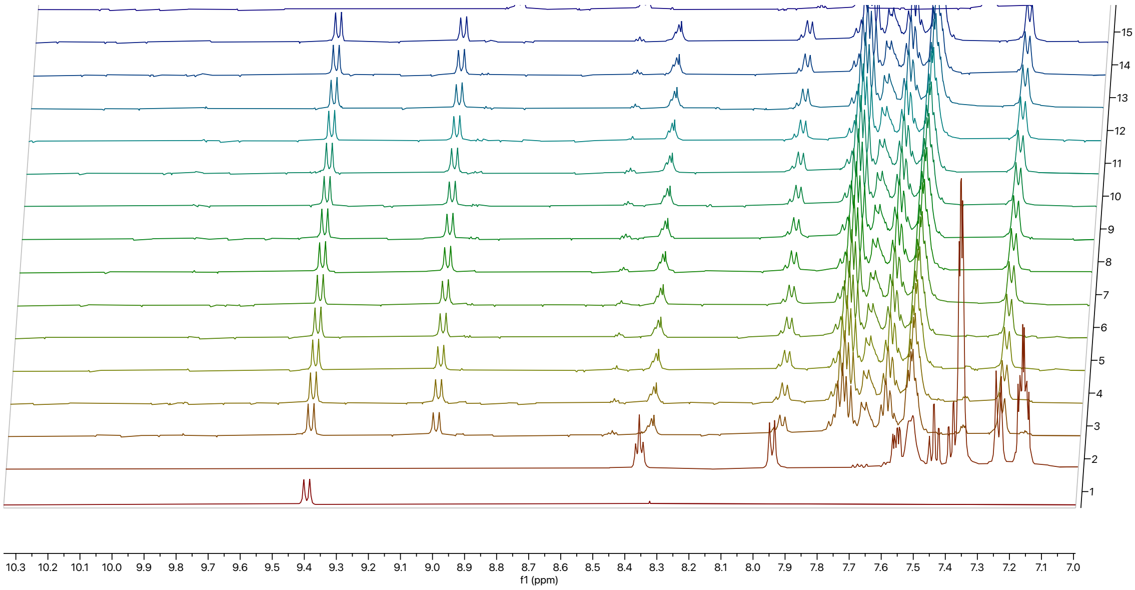

Fig. S4. Stacked ^{1}H NMR spectra showing the progression of the Staudinger reaction between **1** and **2c** in DMSO-d6. Spectrum no. **1** (y-axis) corresponds to the ^{1}H NMR spectrum of compound **1** and spectrum no. **2** corresponds to the ^{1}H NMR spectrum of compound **2c**.

Stability studies

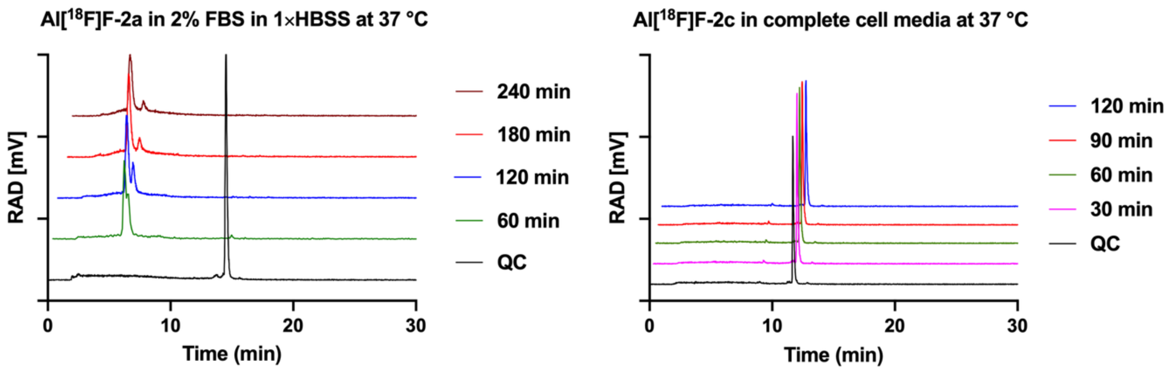

Fig. S5. Radio-HPLC radiolabel stability assays, the chromatogram in black shows the purified product in the quality control (QC) sample and color chromatograms represent the radio-HPLC analysis at predetermined time points. **A**. **Al[^{18}F]F-2a** in 2% FBS in 1×HBSS at 37 °C: intact product retention time 14.58 min and decomposition products 5–7 min. **B**. **Al[^{18}F]F-2c** in complete cell media at 37 °C: HPLC retention time 11.47 min. Chromatograms were plotted from exported ASCII data files using Prism 10.

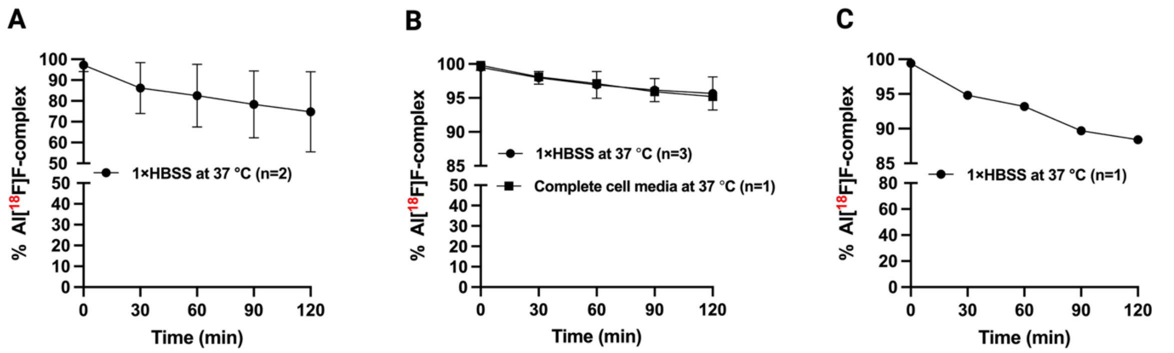

Fig. S6. Radio-iTLC analyses of radiolabel stability assays, the percentage of intact **Al[^{18}F]F-complex** is quantified at 0 min (QC sample), followed by predetermined time points. **A**. **Al[^{18}F]F-2b** in 1×HBSS (n=2) at 37 °C. **B**. **Al[^{18}F]F-2c** in 1×HBSS (n=2) and complete cell media (n=1) at 37 °C. **C**. **Al[^{18}F]F-2c-oxide** in 1×HBSS (n=1) at 37 °C. The values represent mean ± s.d when n>1 for experiments performed in replicate (n>1). Data was plotted using Prism 10.

LC-MS analysis of the Staudinger product 3c

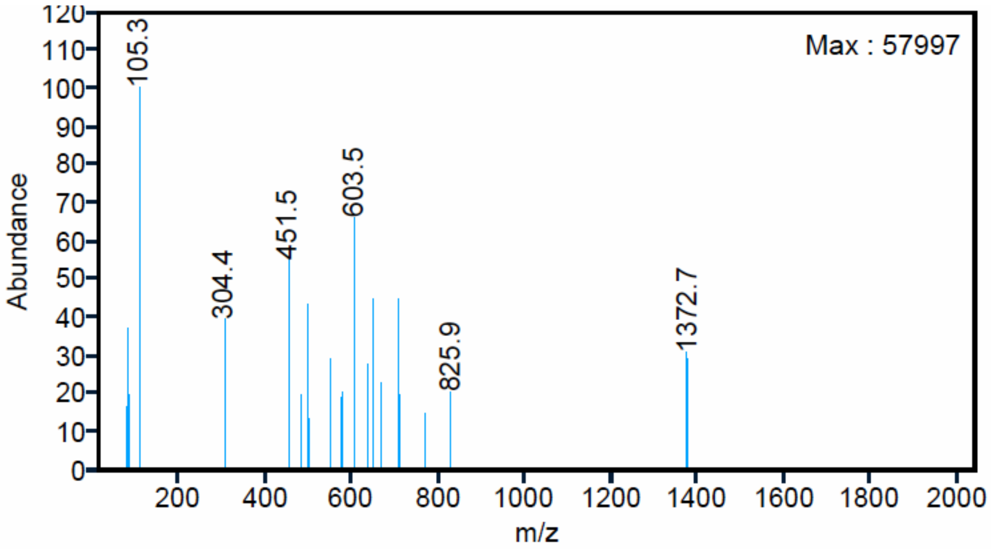

Fig. S7. LC-MS spectrum of Staudinger product **3c** between **1** and **2c**.

LC-MS (ESI) m/z: $[M+H]^+$ Calcd for $C_{68}H_{75}F_4N_6O_{18}P$ 1372.5; Found 1372.7.

The product was also purified with semiprep-HPLC (**method C**) and analyzed with HRMS but no molecular ions were visible.

Radiochemistry

General

Radioactivity was measured using an ionization chamber-based activity meter (Capintec Radioisotope Calibrator CRC-721, Ramsey, NJ, USA). The cartridges used for the purification of radiolabeled compounds are from Waters (Milford, MA, USA): Oasis WAX Plus Short Cartridge (product no. 186003519), Sep-Pak Alumina N Plus Light Cartridge (product no.WAT023561) and Macherey-Nagel (Düren, Germany): CHROMAFIX Alox N Large (product no. 731845) and CHROMAFIX Alox N Medium (product no. 731844). The radiolabeled compounds were analyzed using iTLC-SG from Agilent (Santa Clara, CA, USA,

product no. SGI0001) with 0.5% AcOH/MeOH as an eluent, and radio-HPLC (method A) using a UV or a DAD detector and a Scionix Holland scintillation detector with a 51 BP 51/2 NaI(Tl) crystal for radiodetection. After air-drying, the iTLC-SG sheet was exposed to a digital imaging plate for photostimulated luminescence (PSL) detection (Fuji BAS-TR2025, 20 × 25 cm) for an appropriate period of time. The imaging plate was scanned on a Fujifilm Fluorescent Image Analyzer (FLA-5100 V.1, Fuji Film Photo, Tokyo, Japan). The radio-TLCs were analyzed using AIDA image analysis software version 5.0 SP 3 (Elysia-Raytest GmbH, Straubenhardt, Germany). All buffers used for radiolabeling were treated with Chelex (100 sodium form (Sigma-Aldrich, St. Louis, MO, USA) and filtered with 0.22 µm filters.

Example of radio-HPLC chromatogram for Al[^{18}F]F-2c

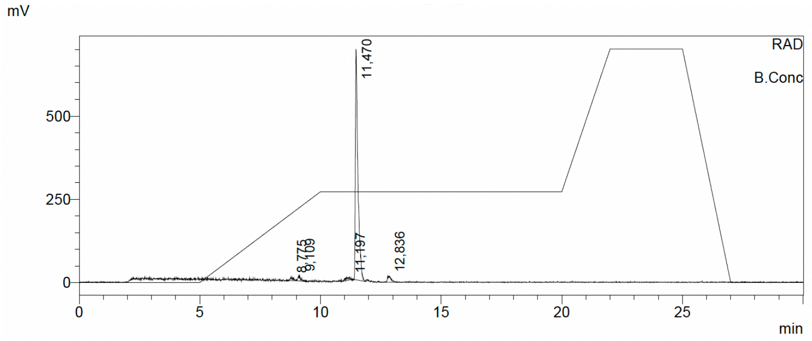

Fig. S8. Example of a radio-HPLC chromatogram of a quality control sample for **Al[^{18}F]F-2c** (entry **e**, Table **2**).

Example of radio-HPLC chromatogram for Al[^{18}F]F-2c-oxide

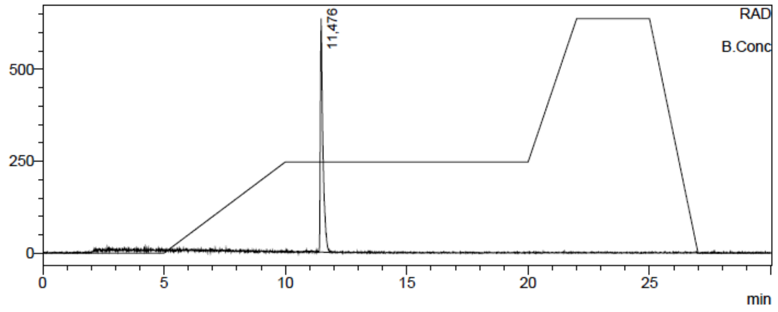

Fig. S9. Example of a radio-HPLC chromatogram of a quality control sample for **Al[^{18}F]F-2c-oxide** (entry **f**, Table **2**).

RadioTLC examples for Al[^{18}F]F-2a

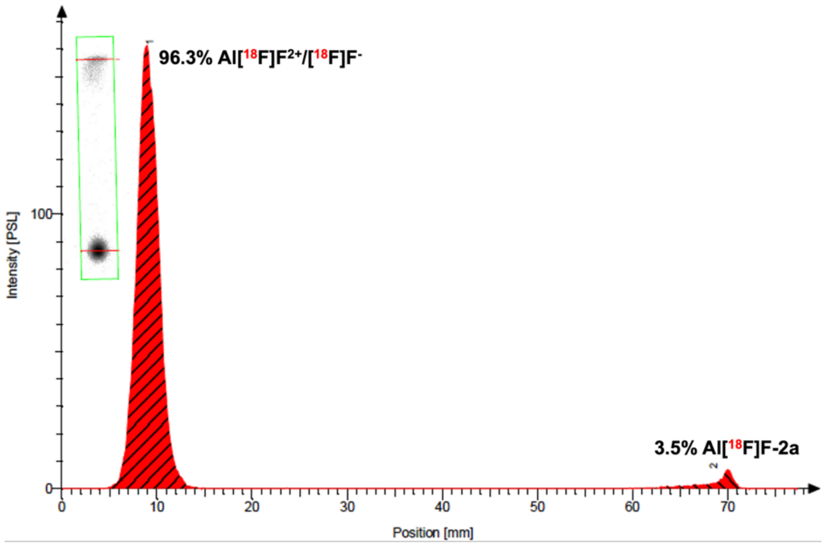

Fig. S10. Radio-TLC example for **Al[^{18}F]F-2a**: entry **i** (25 min), **Table 1**.

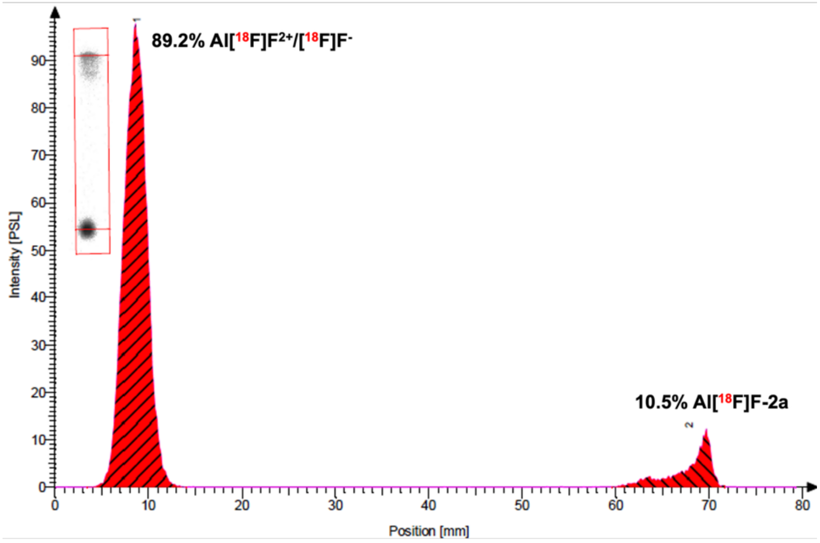

Fig. S11. Radio-TLC example for **Al[^{18}F]F-2a**: entry **i** (30 min), **Table 1**.

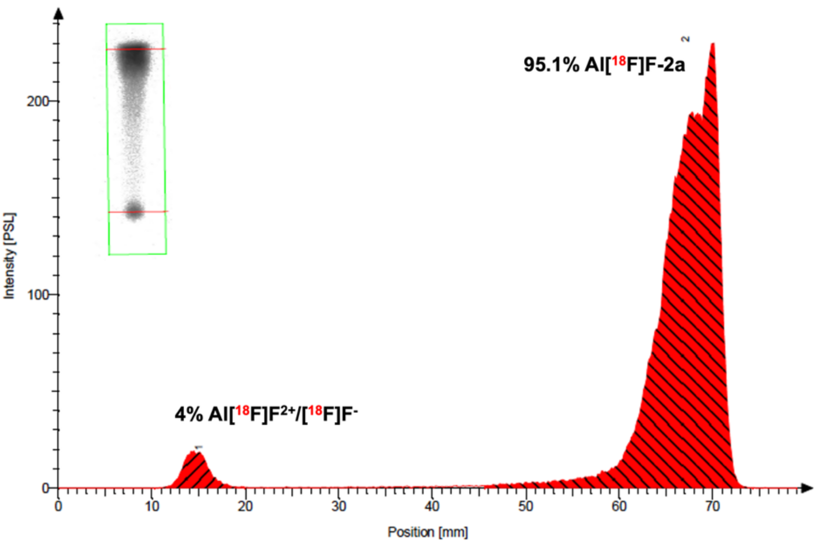

Fig. S12. Radio-TLC example for **Al[^{18}F]F-2a**: entry **d**, Table **1.**

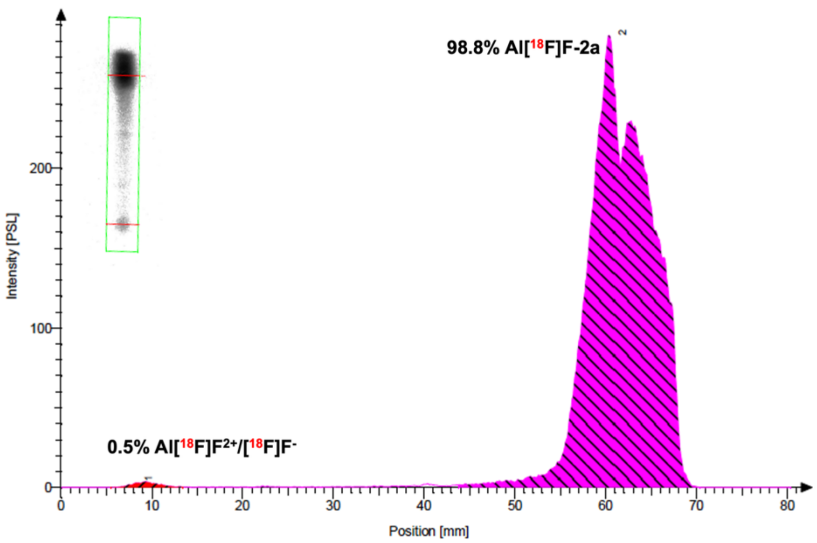

Fig. S13. Radio-TLC example for **Al[^{18}F]F-2a**: purified product after WAX cartridge purification.

RadioTLC examples for Al[^{18}F]F-2b

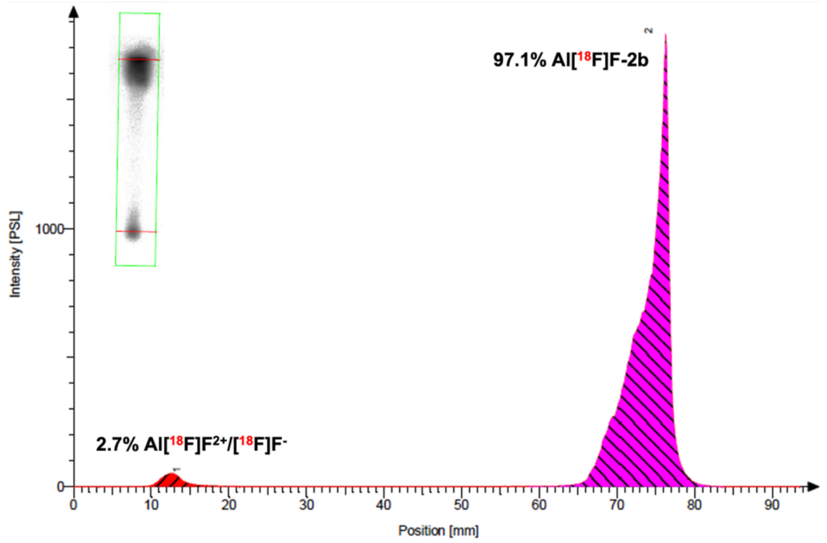

Fig. S14. Radio-TLC example for **Al[^{18}F]F-2b:** entry **a**, Table **2**.

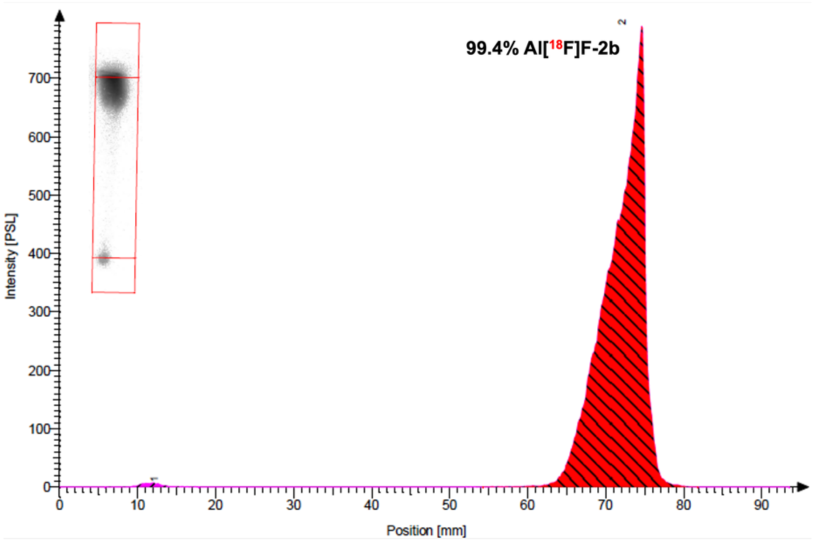

Fig. S15. Radio-TLC example for **Al[^{18}F]F-2b**: purified product after Alox N cartridge purification.

RadioTLC examples for Al[^{18}F]F-2c

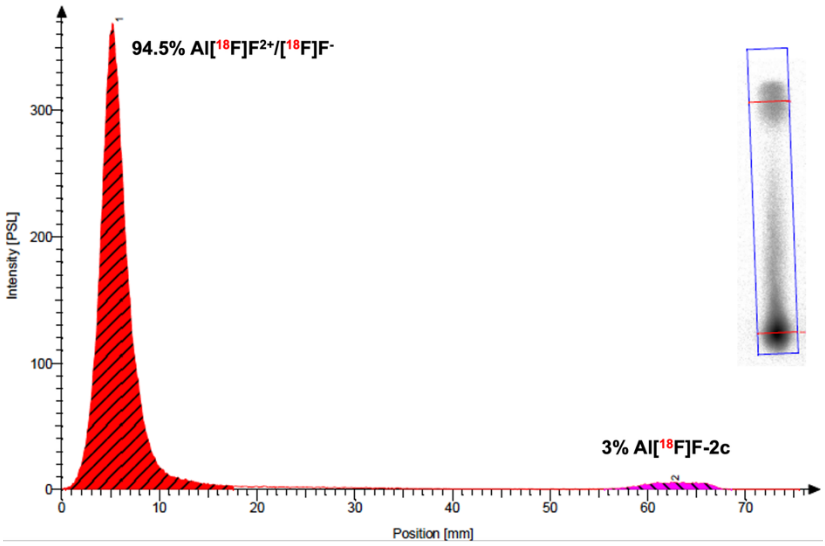

Fig. S16. Radio-TLC example for **Al[^{18}F]F-2c**: entry **c**, Table **2**.

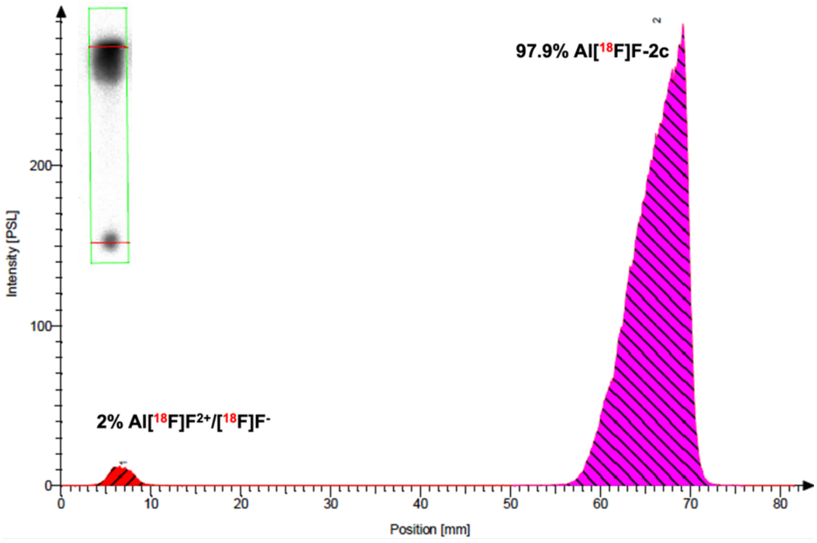

Fig. S17. Radio-TLC example for **Al[^{18}F]F-2c**: entry **e**, Table **2**.

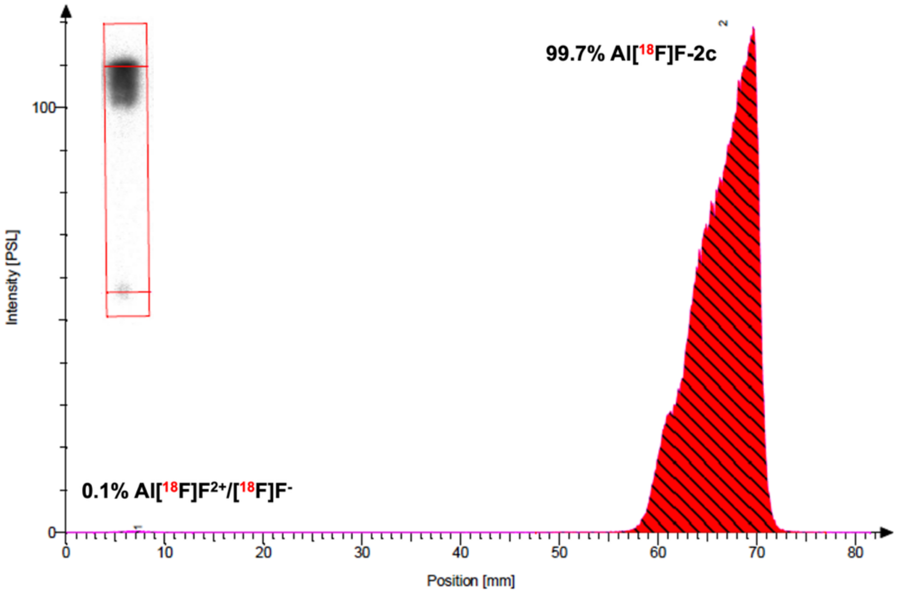

Fig. S18. Radio-TLC example for **Al[^{18}F]F-2c**: purified product after Alox N cartridge purification

RadioTLC examples for Al[^{18}F]F-2c-oxide

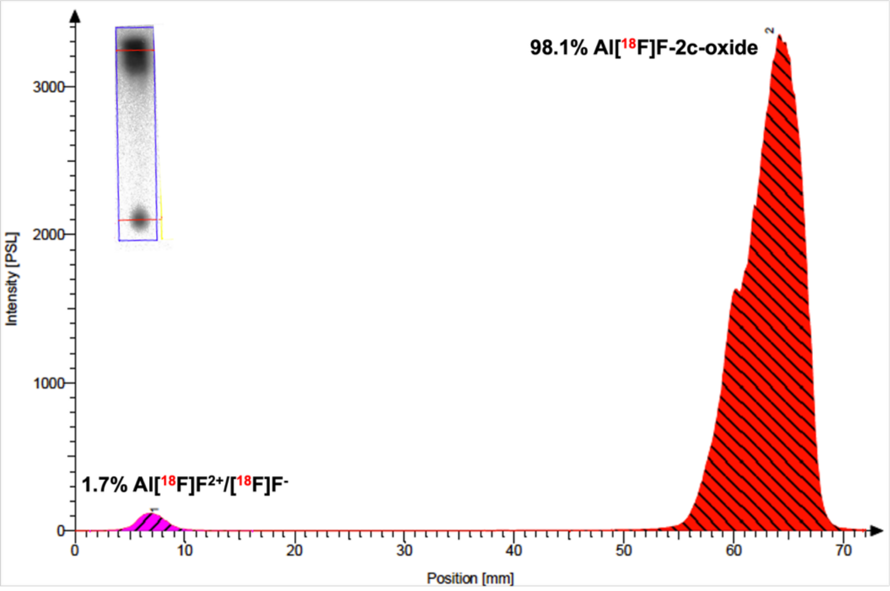

Fig. S19. Radio-TLC example for **Al[^{18}F]F-2c-oxide:** entry **f**, Table **2**.

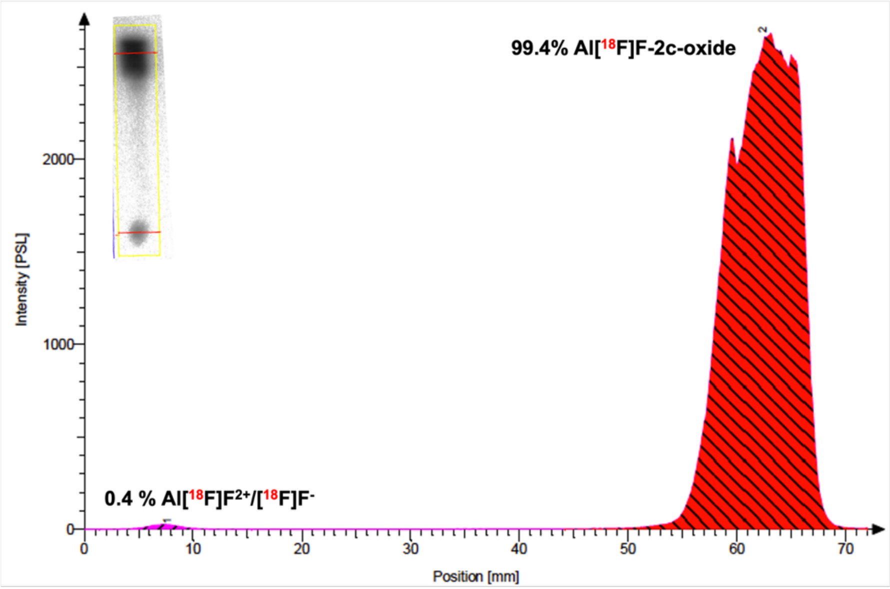

Fig. S20. Radio-TLC example for **Al[^{18}F]F-2c-oxide:** purified product after Alox N cartridge purification.

Cell studies

Cell culturing materials and methods

T25 and T75 cell culturing flasks and multiwell-plates were purchased either from Thermo Fisher Scientific (Waltham, MA, USA), Avantor (Radnor, PA, USA) or Corning Inc. (Corning, NY, USA). Dulbecco's phosphate buffer saline (10×DPBS), Roswell Park Memorial Institute 1640 medium (RPMI-1640), GlutaMAX (100×), fetal bovine serum (FBS), Penicillin-Streptomycin (10,000 U/ml), and TrypLETM (1×) were purchased from Gibco (Life Technologies Gibco, Carlsbad, CA, USA). All culturing media were supplemented with 10% FBS, 1×GlutaMAX and 1% PS and aseptically filtered through sterilized 0.22 μm filter unit before use. The cell incubator was set at 37 ºC with 5% CO_2 and 95% relative humidity. The cell cytotoxicity data was plotted using Prism 10 software (GraphPad Software, LLC, Boston, MA, USA).

Flow cytometry

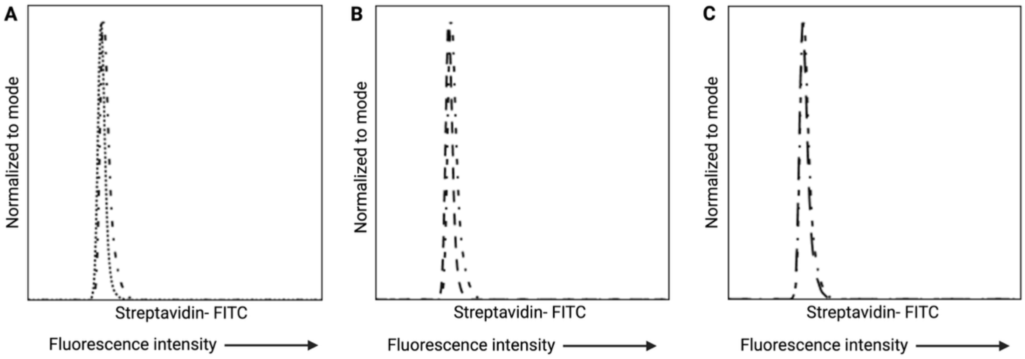

	Dotted line	Complex line	Dashed line	Complex line	Long dashed line	Complex line
1	–	+	–	+	–	+
$Ac_4ManNAz$	–	–	–	–	–	–
Phosphine-PEG3-Biotin	–	+	+	+	+	+

Fig. S21. Flow cytometry analysis of live Jurkat cells after a 72 h treatment with 1×PBS (dotted line in **A** and long dashed line in **C**) or MGE with **1** (complex line in **A**–**C**), followed by incubation with 1×PBS (dotted line in **A**) or 1 μM Phosphine-PEG3-Biotin (in all the other conditions) and finally incubation with Streptavidin-FITC (**A**–**C**).

NMR spectra

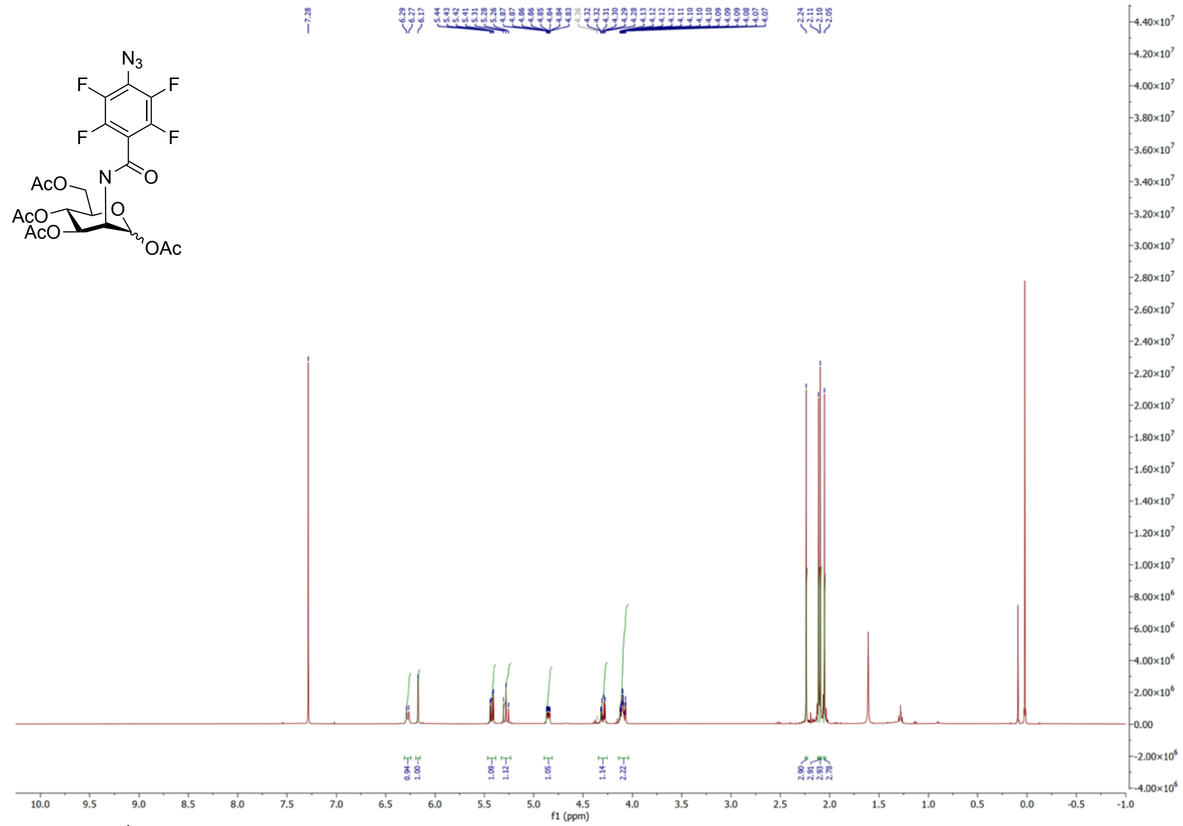

Fig. S22. ^{1}H NMR spectrum of compound **1** in $CDCl_3$.

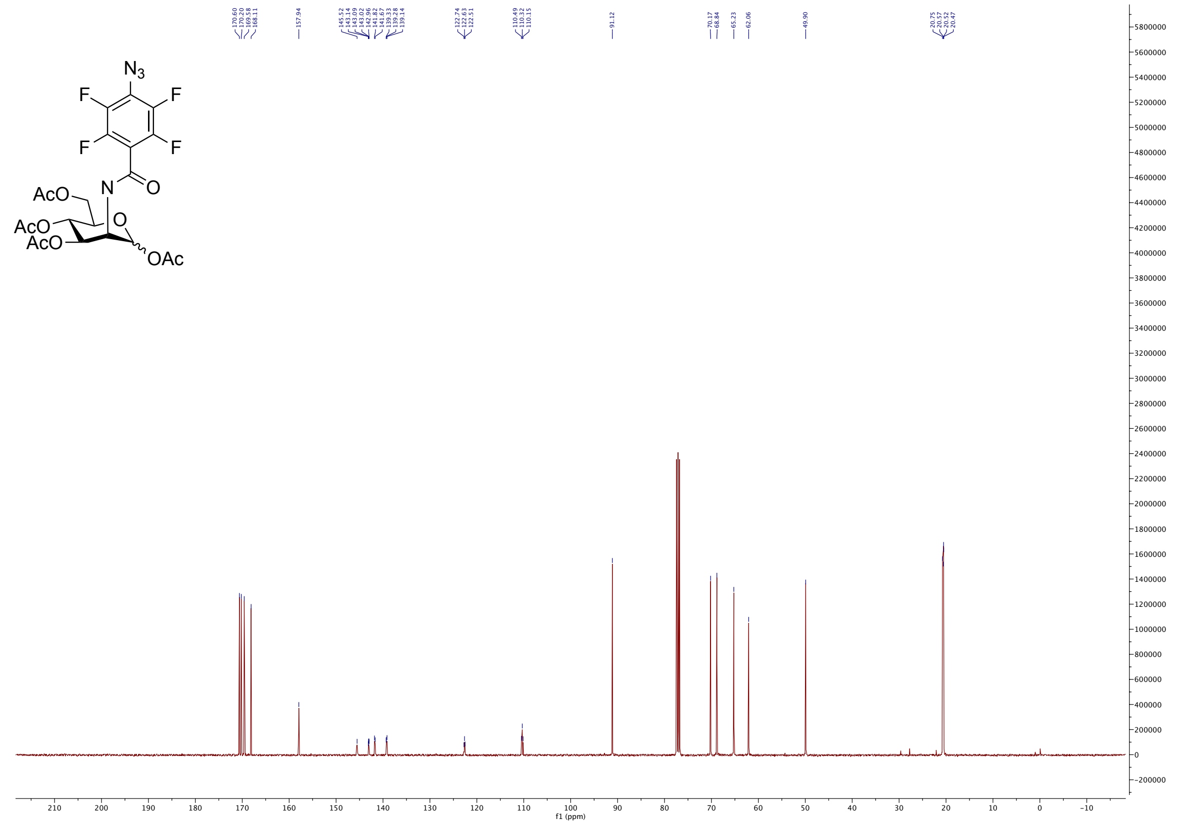

Fig. S23. ^{13}C NMR spectrum of compound **1** in $CDCl_3$.

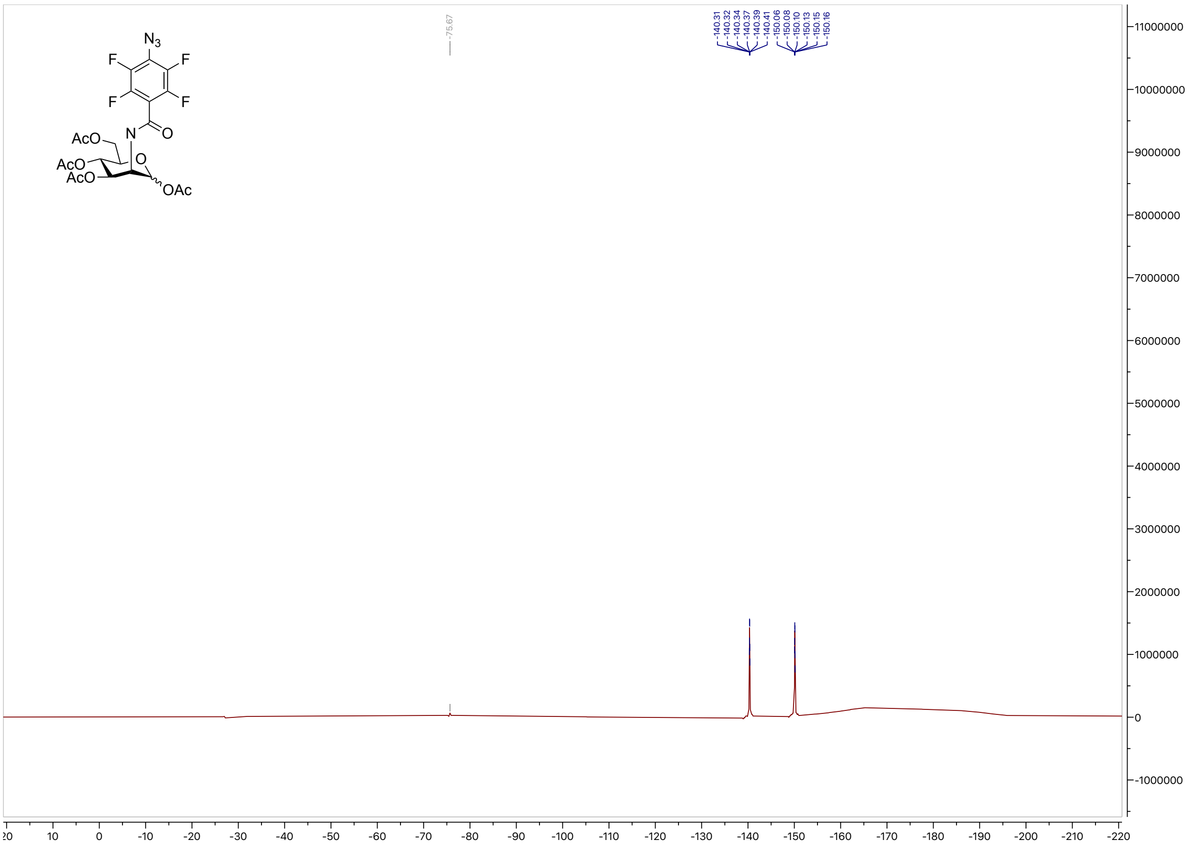

Fig. S24. ^{19}F NMR spectrum of compound **1** in $CDCl_3$.

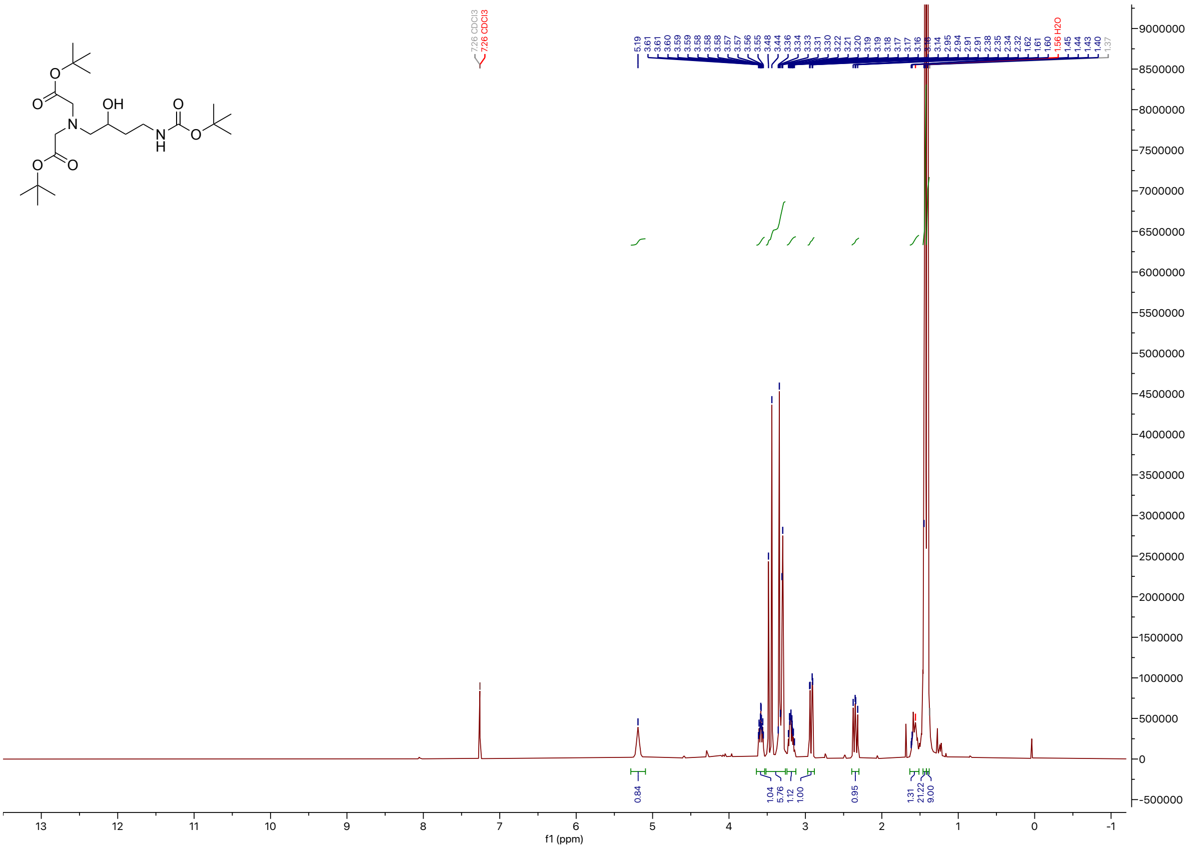

Fig. S25. ^{1}H NMR of compound **9** in $CDCl_3$.

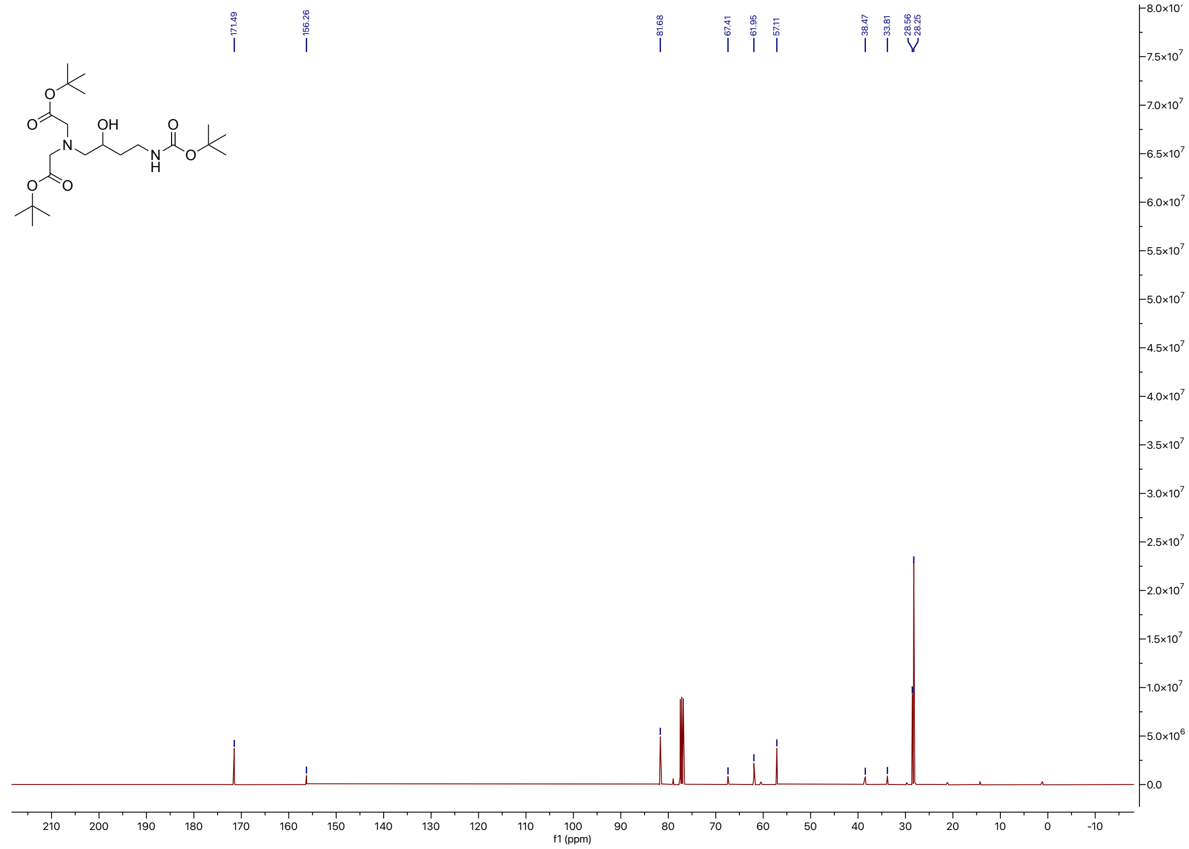

Fig. S26. ^{13}C NMR of compound **9** in $CDCl_3$.

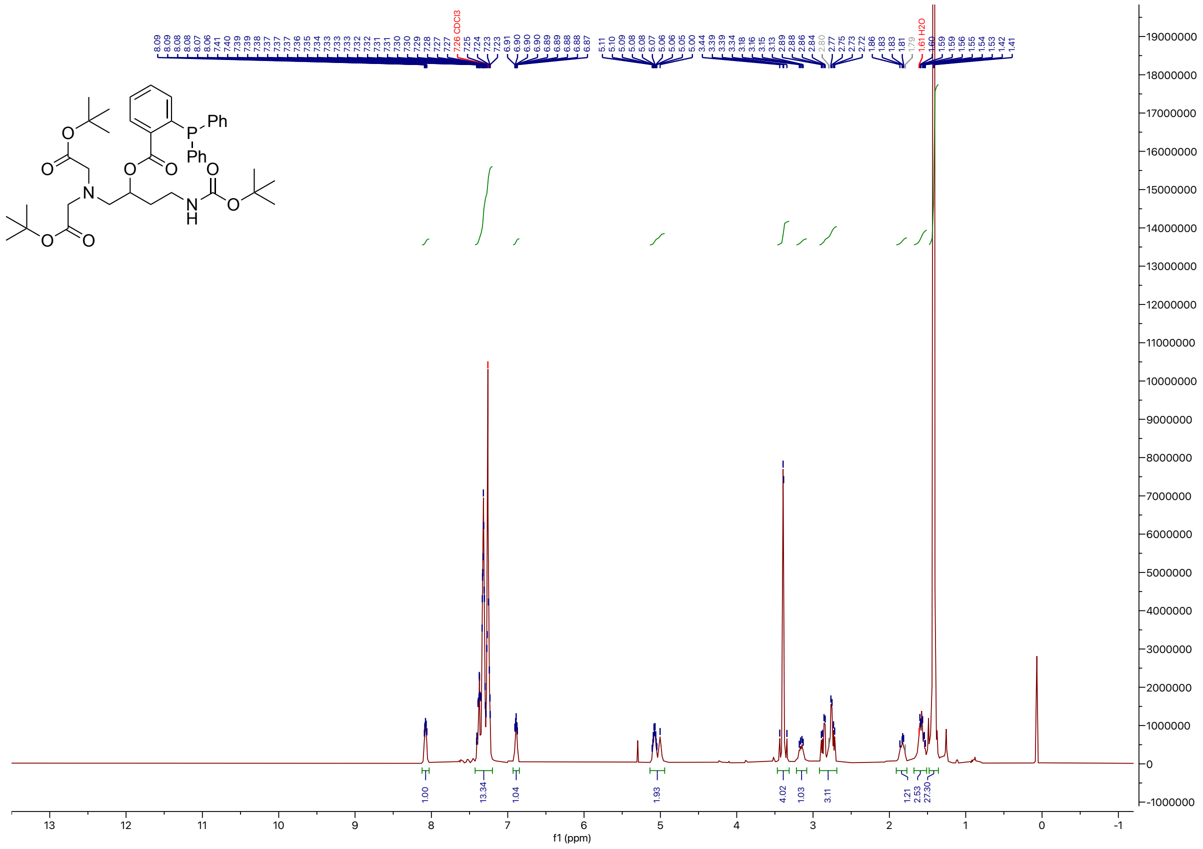

Fig. S27. ^{1}H NMR of compound **10** in $CDCl_3$.

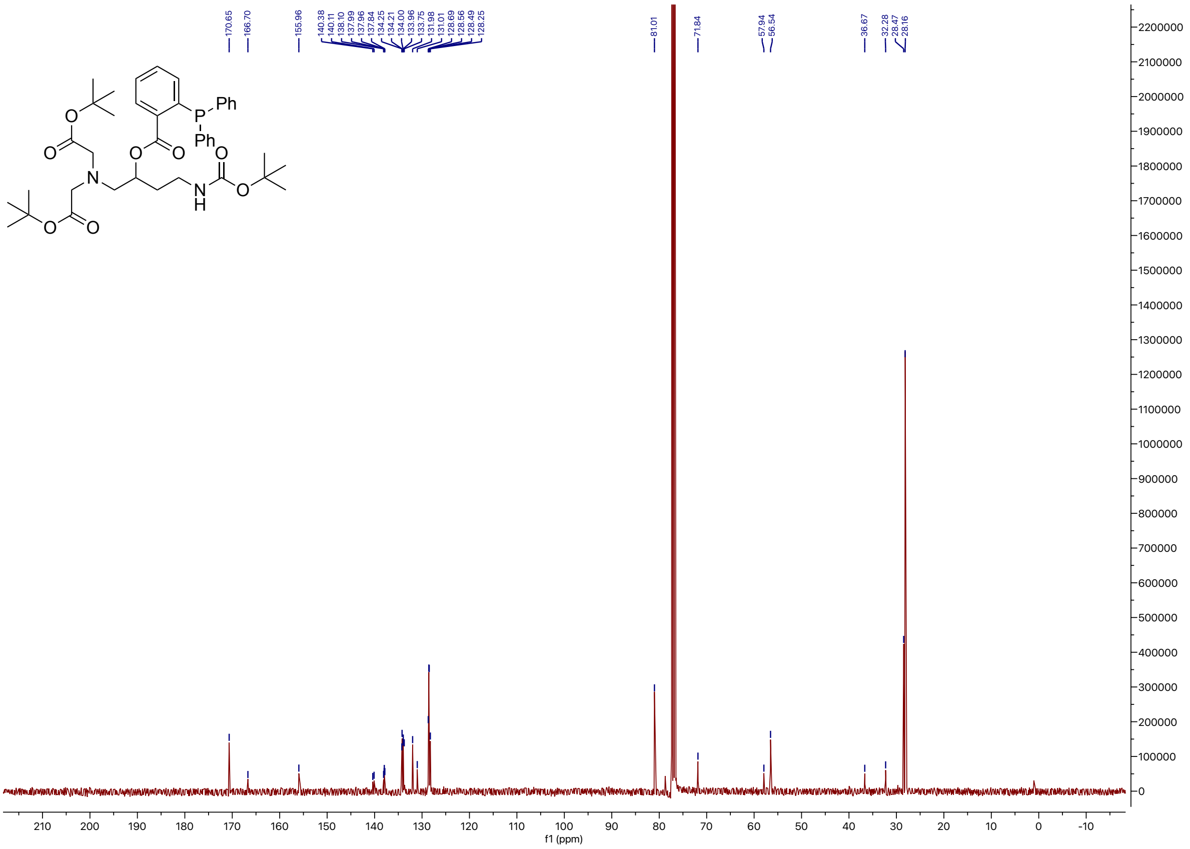

Fig. S28. ^{13}C NMR of compound **10** in $CDCl_3$.

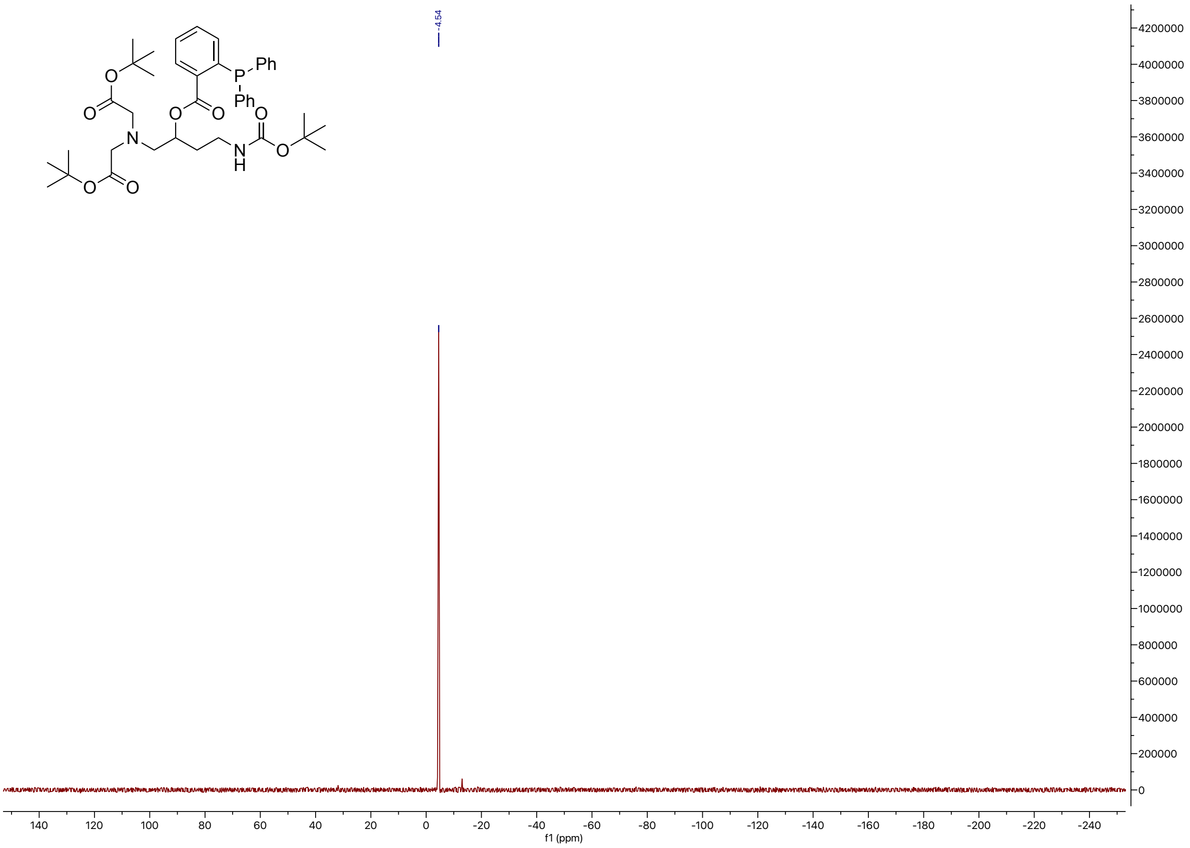

Fig. S29. ^{31}P NMR of compound **10** in $CDCl_3$.

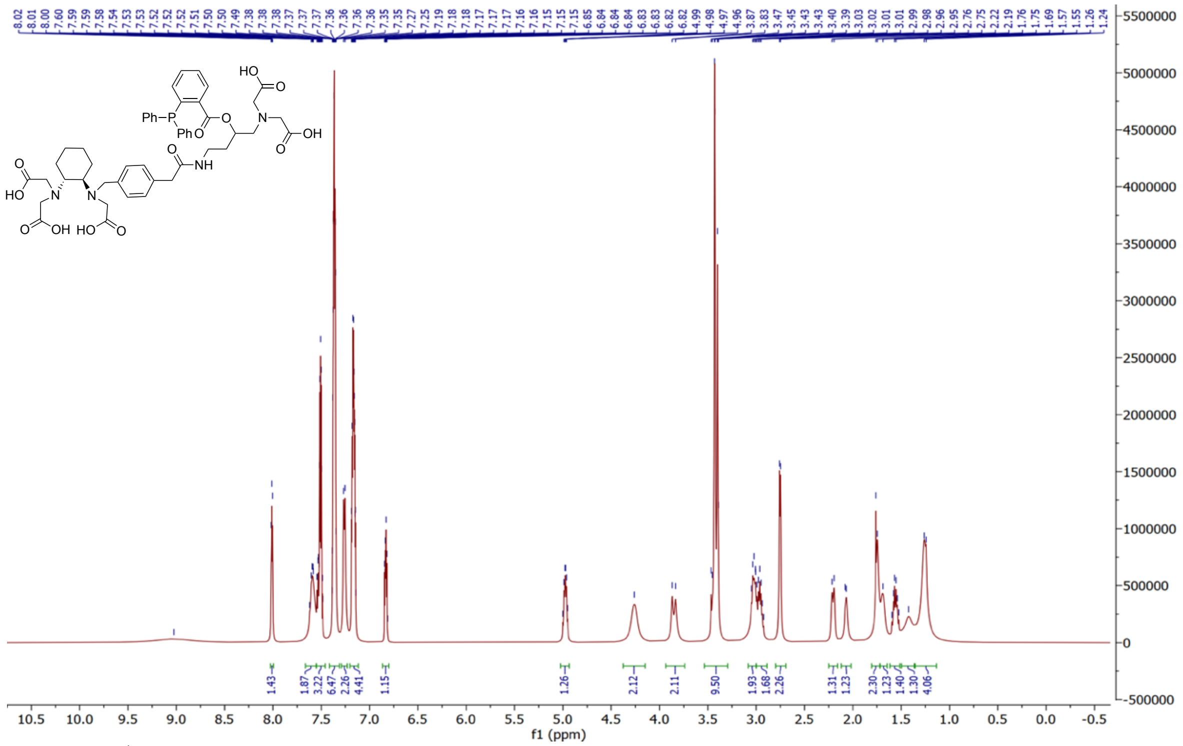

Fig. S30. ^{1}H NMR spectrum of compound **2a** in DMSO-d_6.

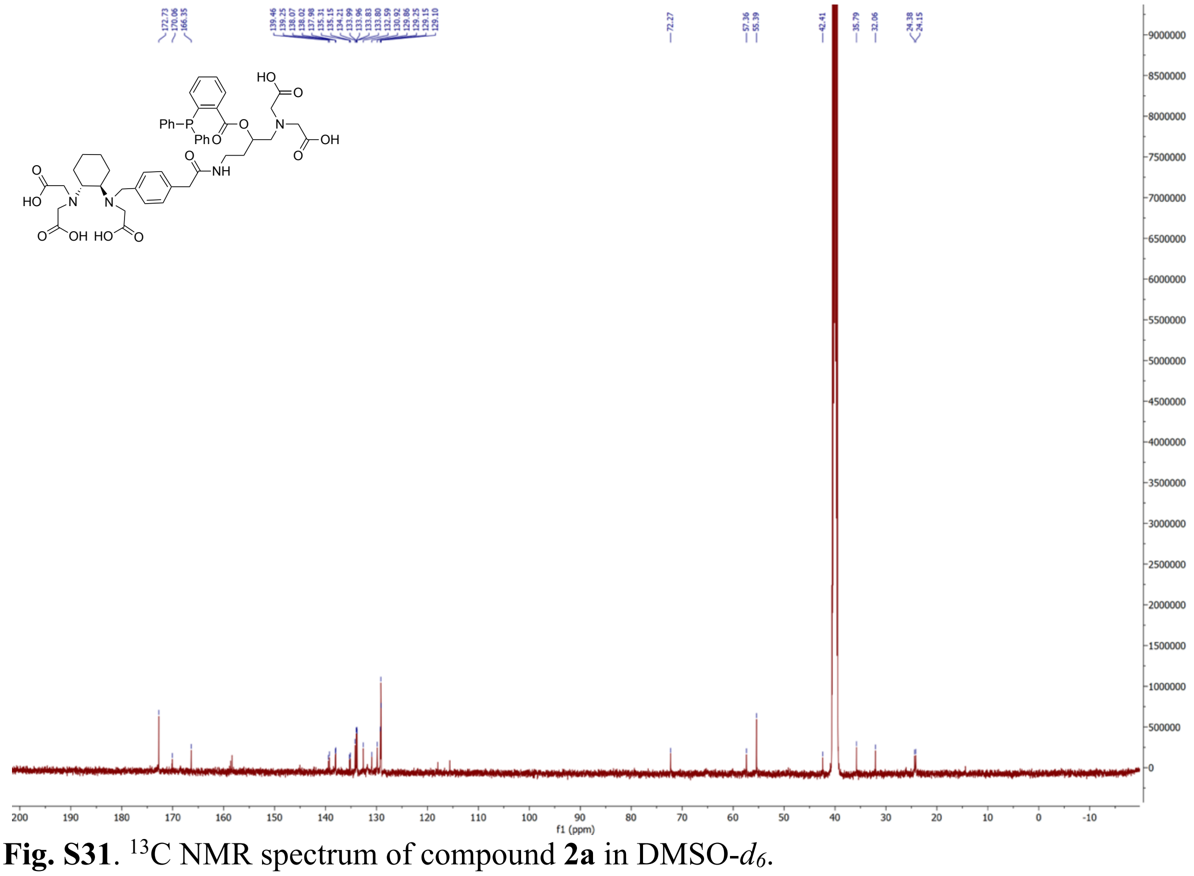

Fig. S31. ^{13}C NMR spectrum of compound **2a** in DMSO-d_6.

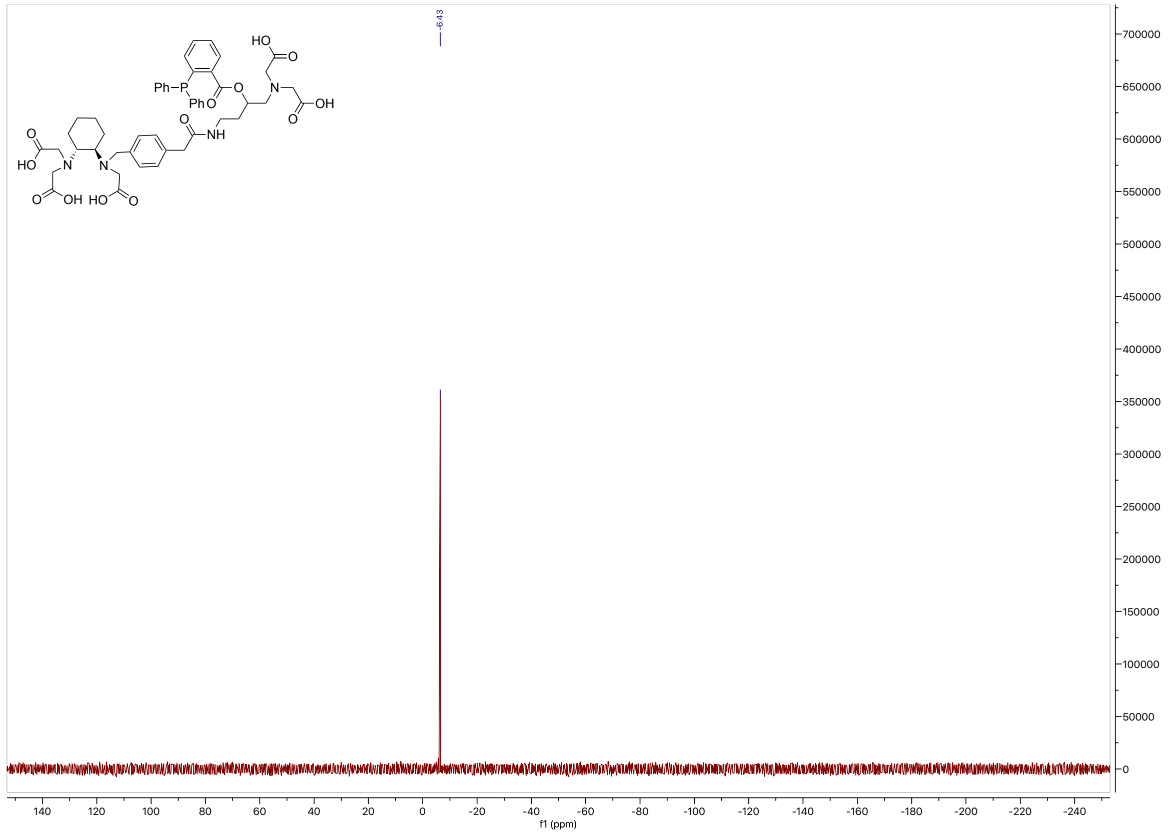

Fig. S32. ^{31}P NMR spectrum of compound **2a** in DMSO-d_6.

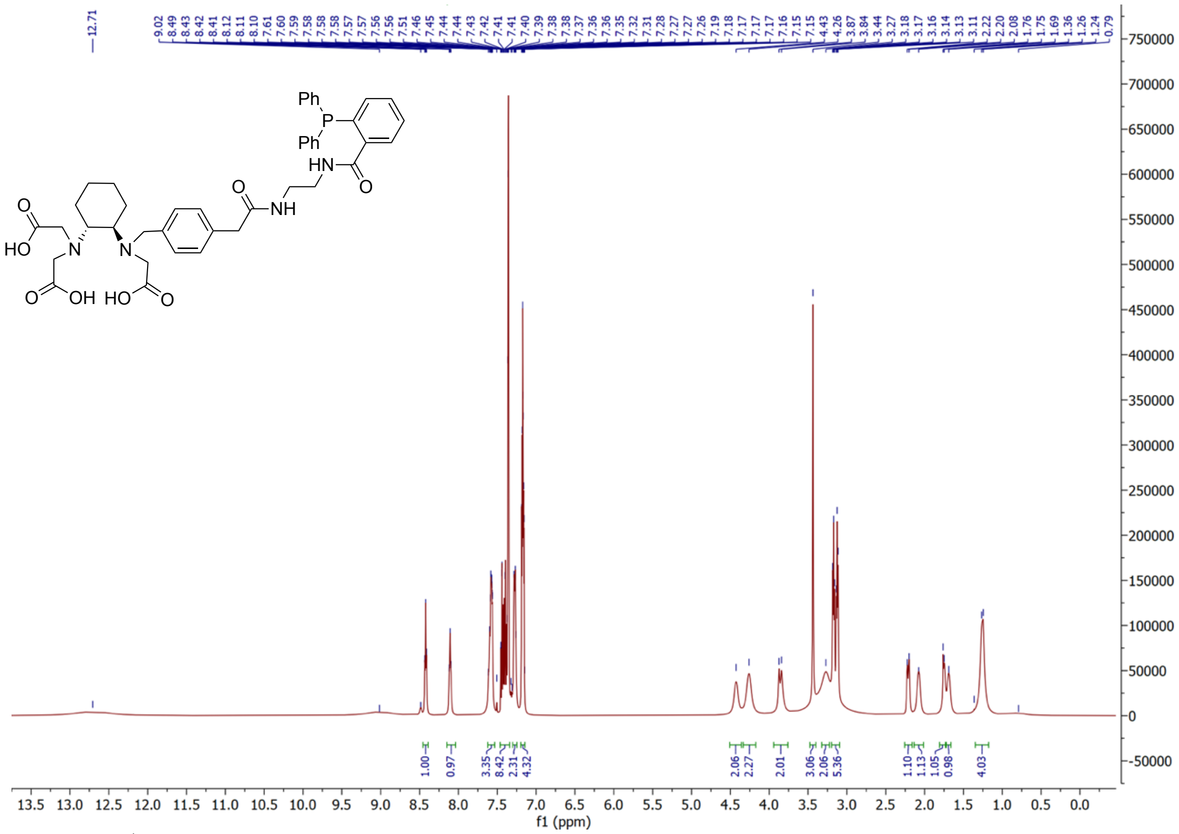

Fig. S33. ^{1}H NMR spectrum of compound **2b** in DMSO-d_6.

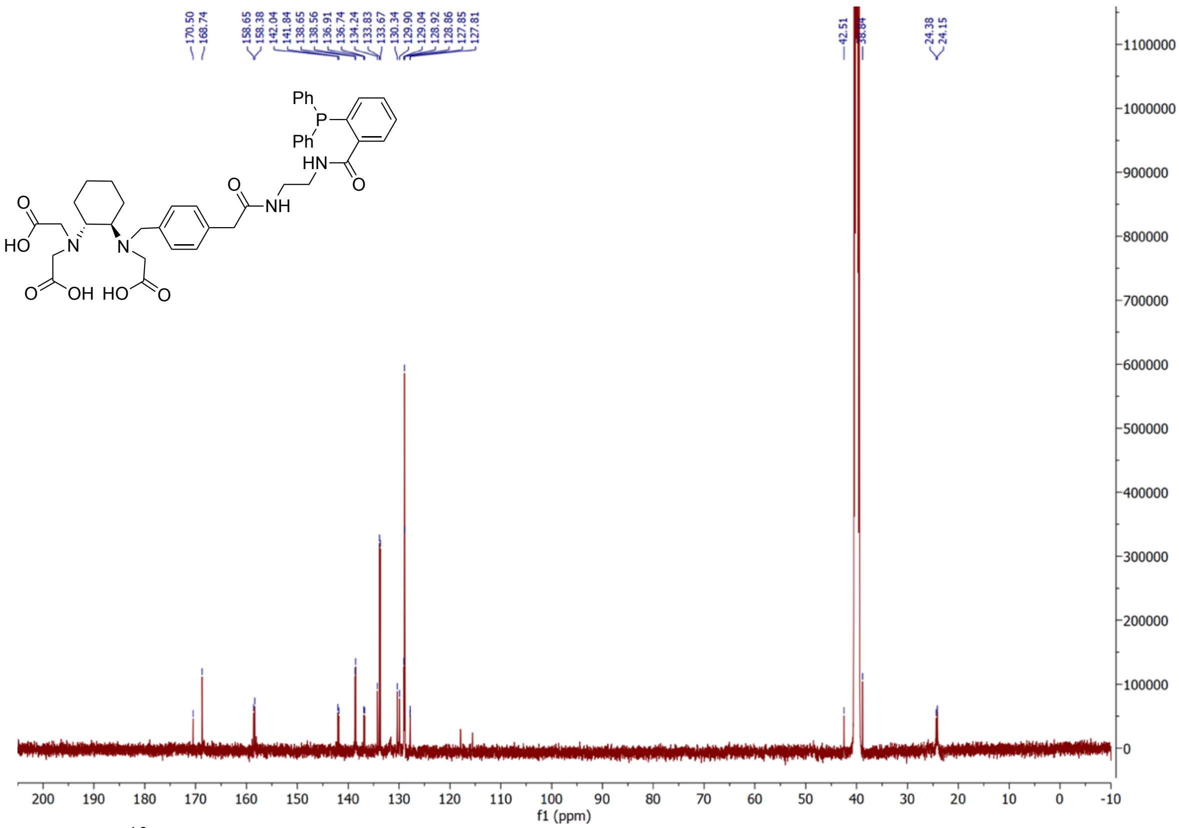

Fig. S34. ^{13}C NMR spectrum of compound **2b** in DMSO-*d*$_6$.

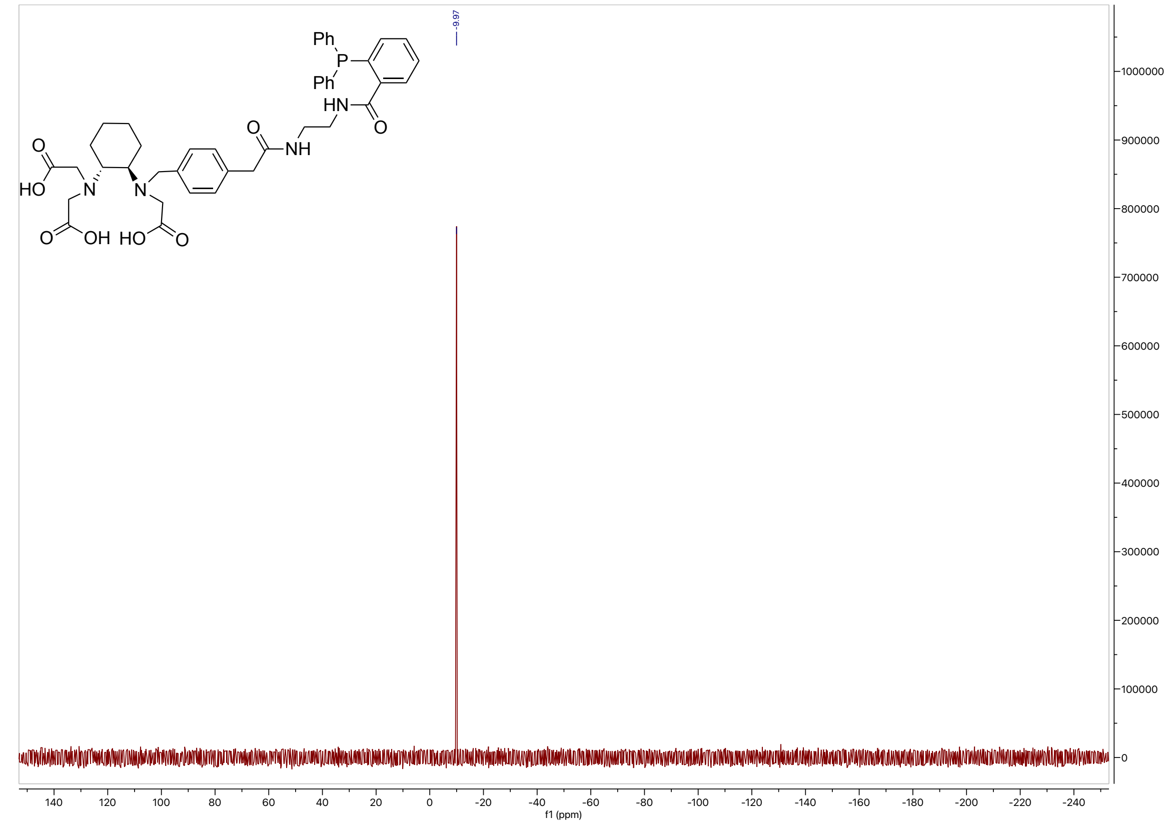

Fig. S35. ^{31}P NMR spectrum of compound **2b** in DMSO-*d*$_6$.

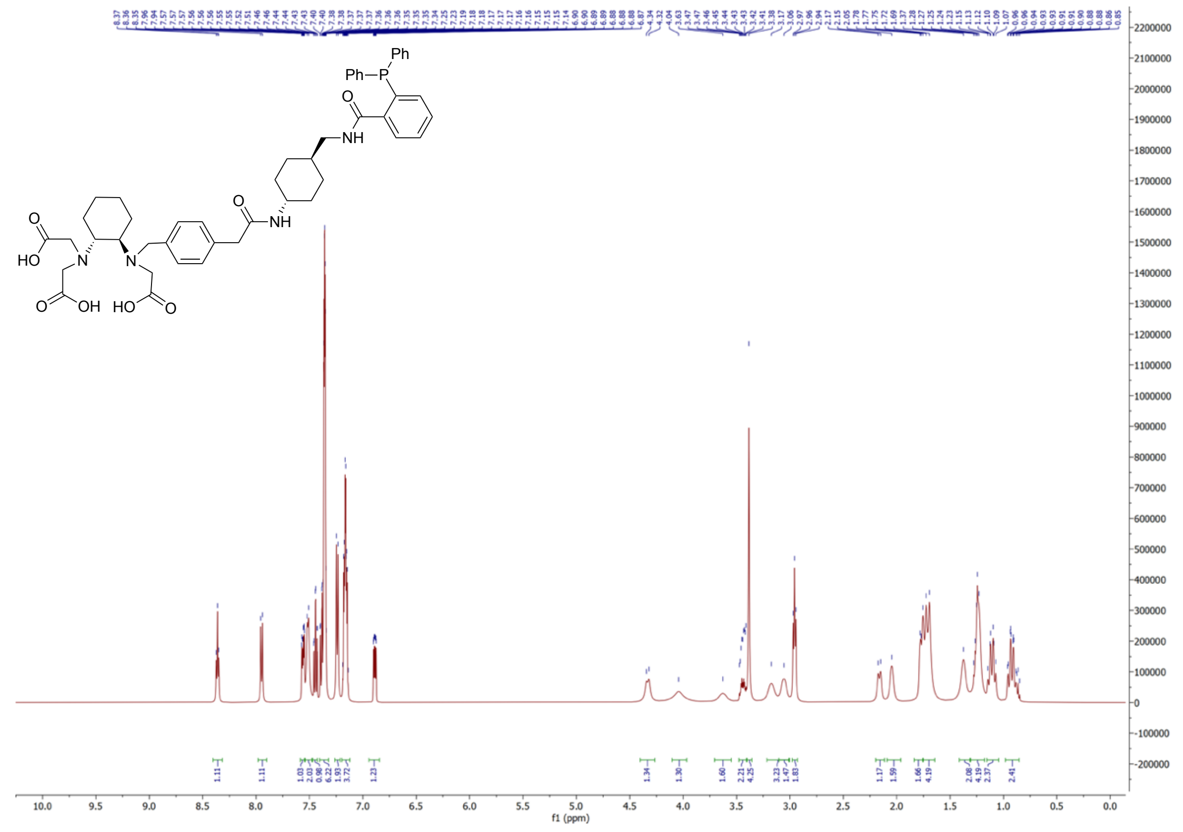

Fig. S36. ^{1}H NMR spectrum of compound **2c** in DMSO-d_6.

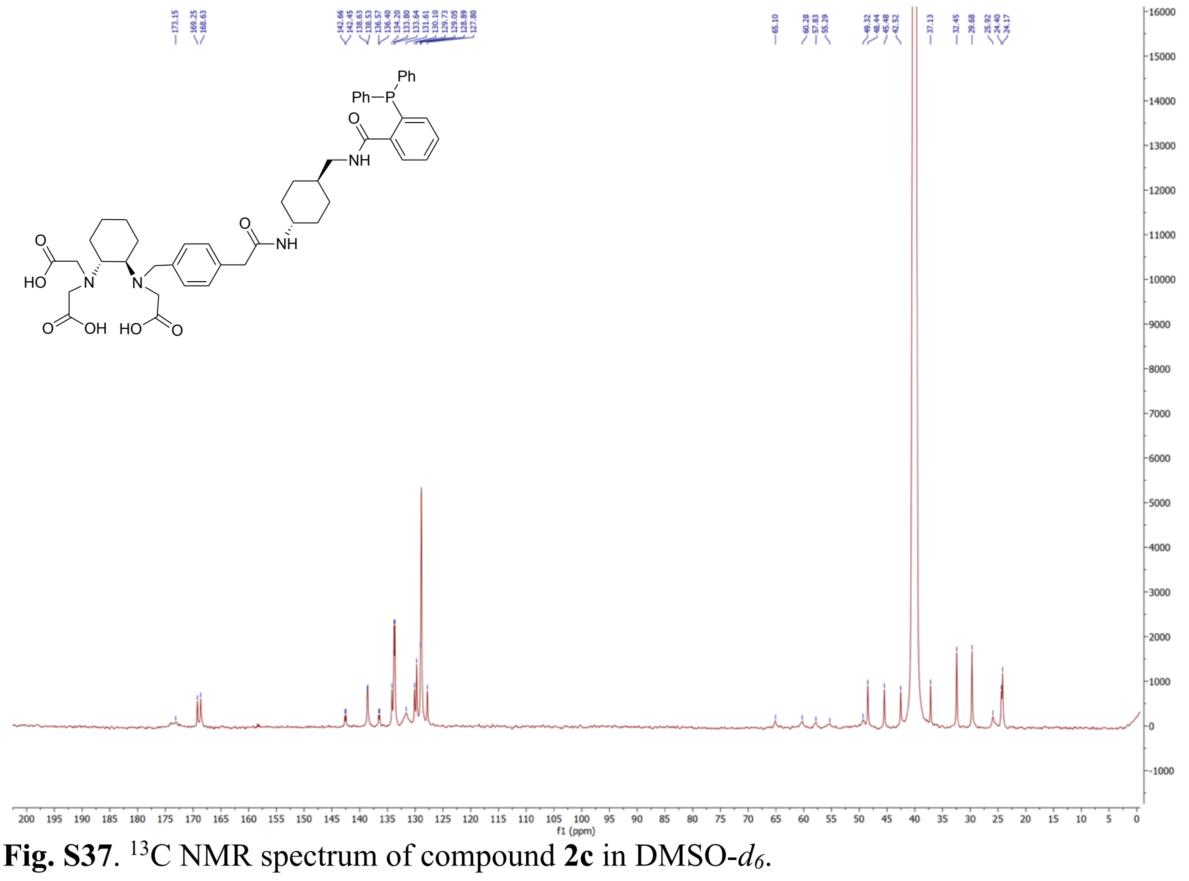

Fig. S37. ^{13}C NMR spectrum of compound **2c** in DMSO-d_6.

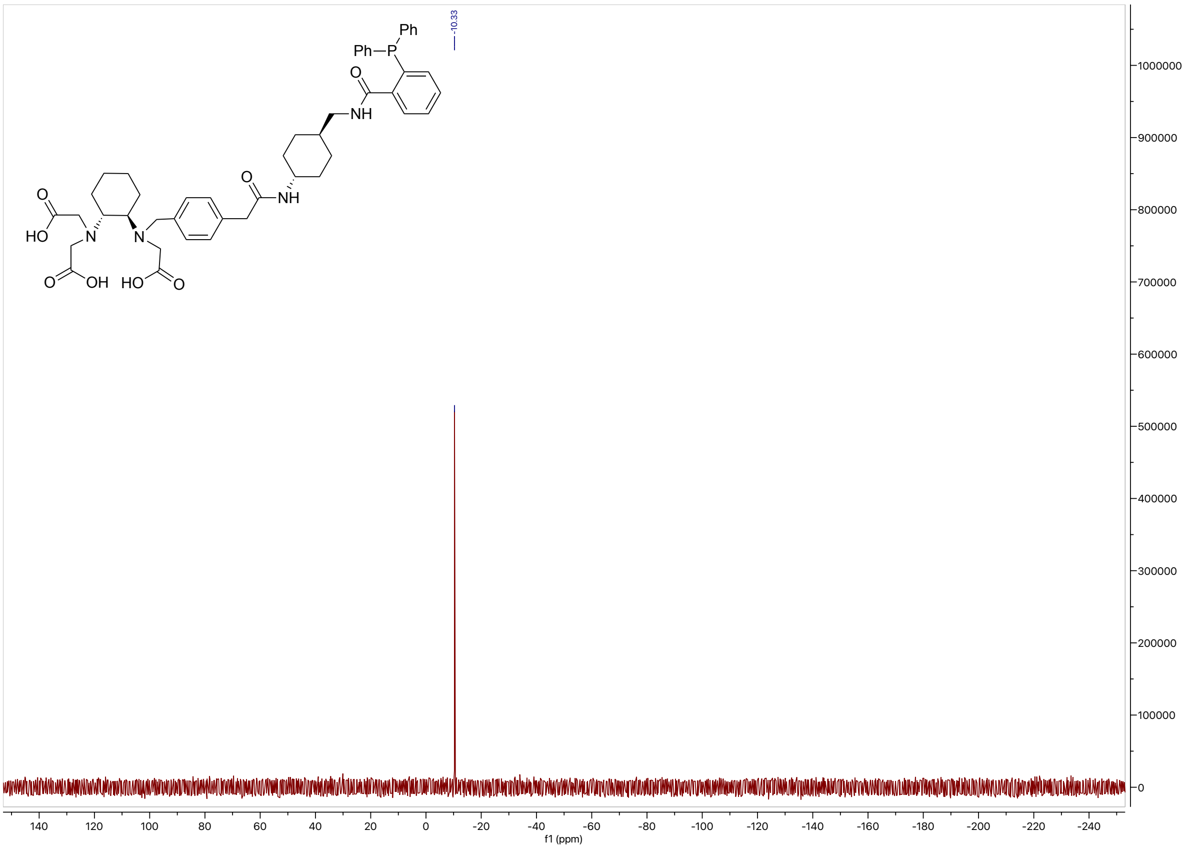

Fig. S38. ^{31}P NMR spectrum of compound **2c** in DMSO-d_6.

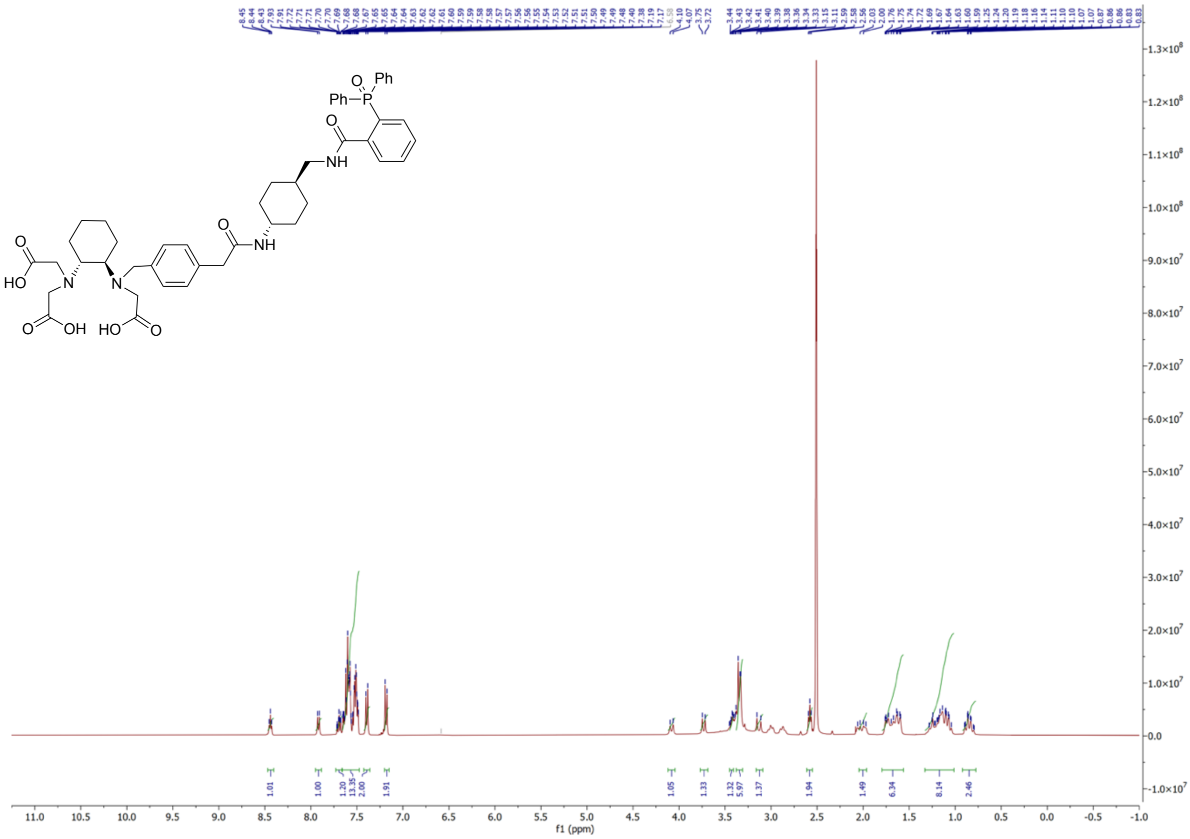

Fig. S39. ^{1}H NMR spectrum of compound **2c-oxide** in DMSO-d_6.

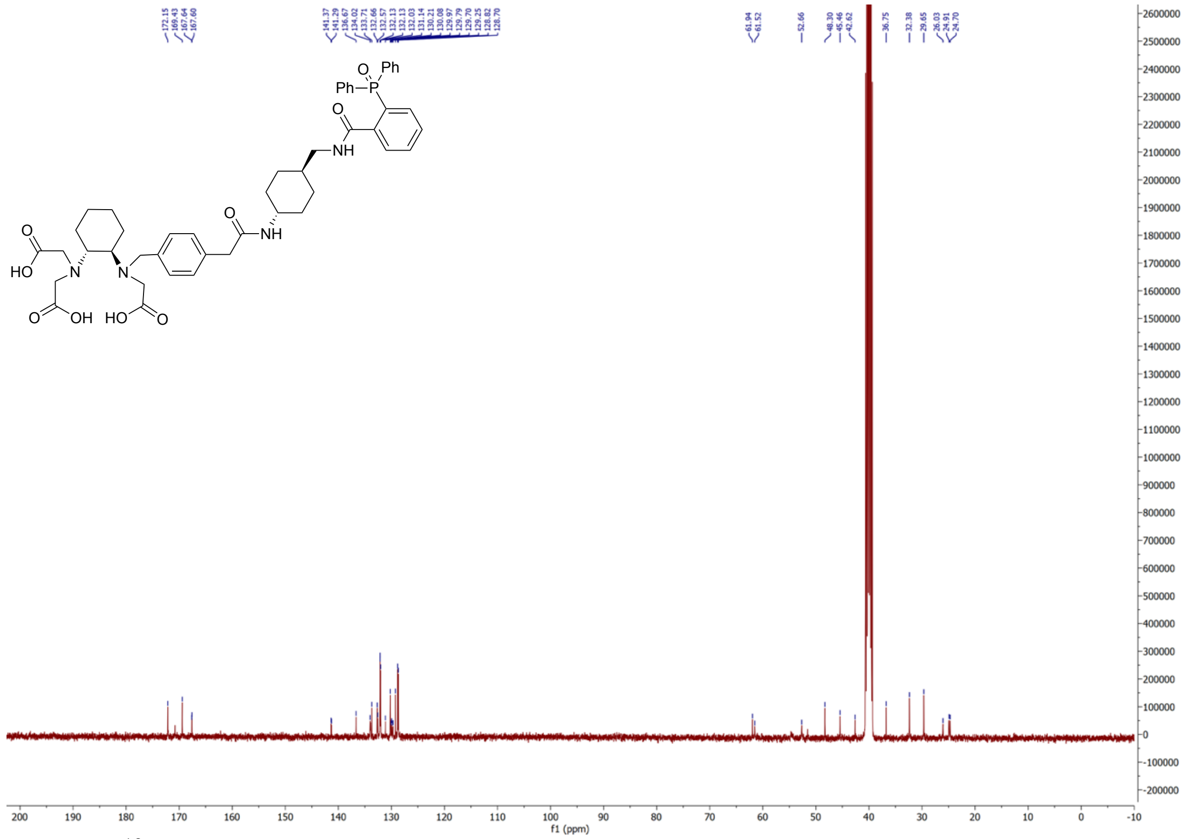

Fig. S40. ^{13}C NMR spectrum of compound **2c-oxide** in DMSO-d_6.

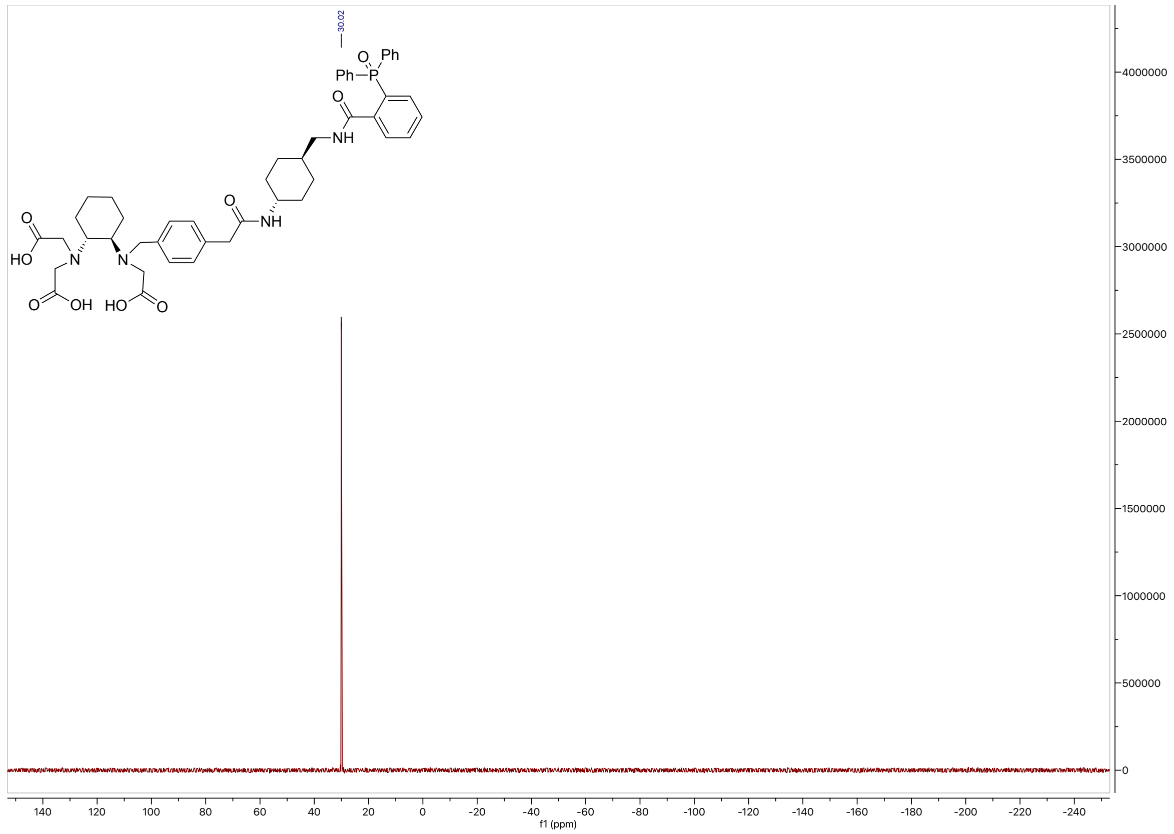

Fig. S41. ^{31}P NMR spectrum of compound **2c-oxide** in DMSO-d_6.

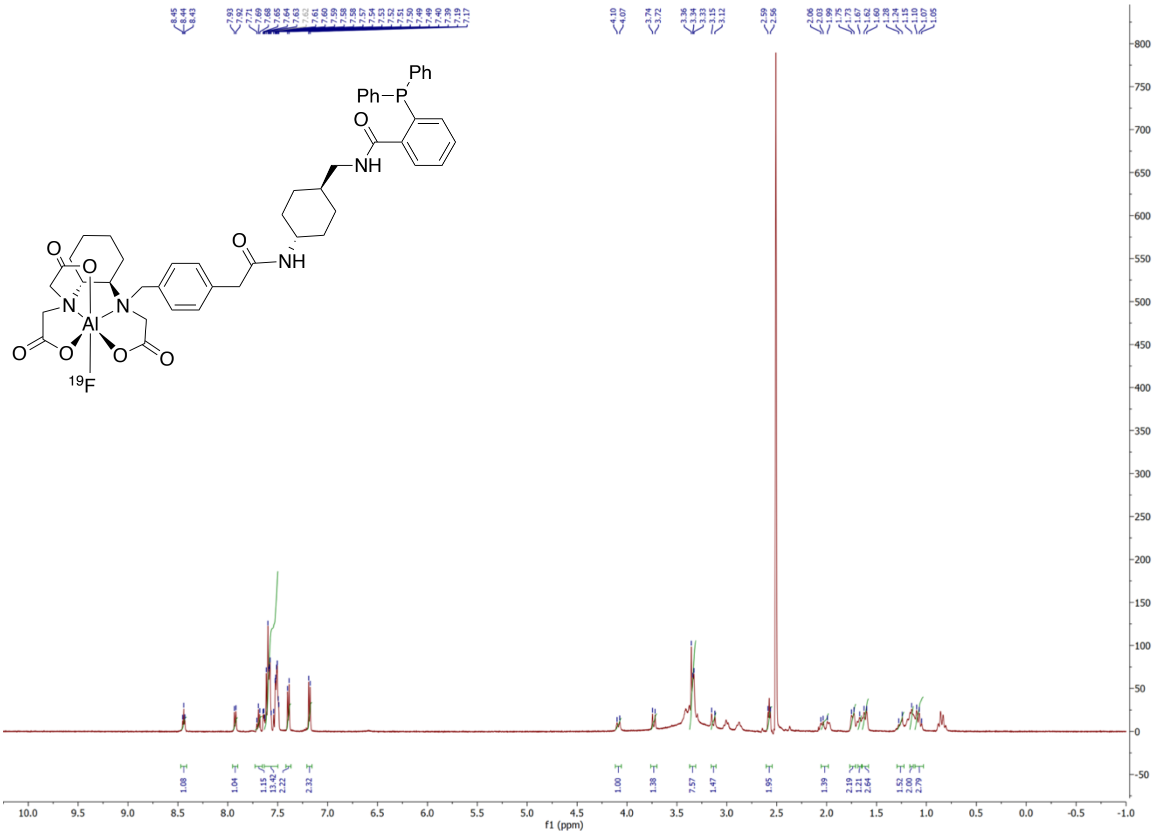

Fig. S42. ^{1}H NMR spectrum of compound **Al^{19}F-2c** in DMSO-d_6.

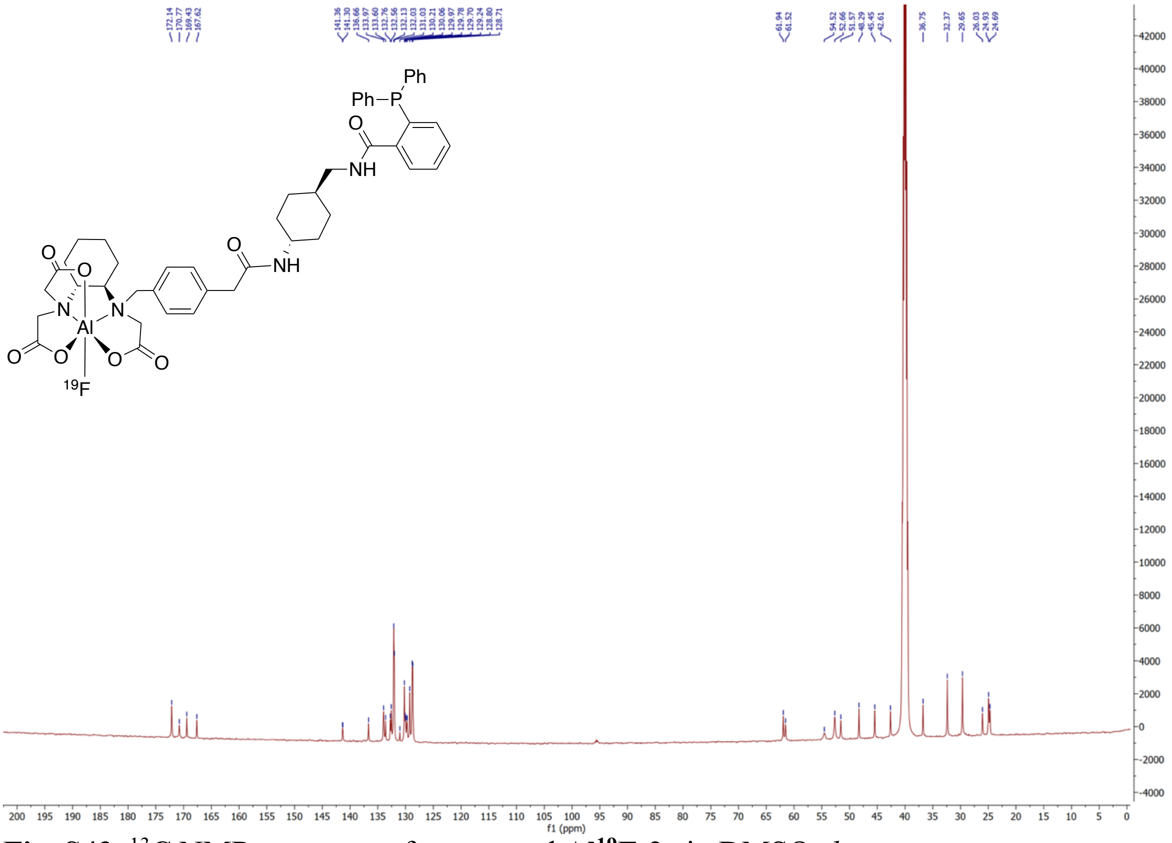

Fig. S43. ^{13}C NMR spectrum of compound **Al^{19}F-2c** in DMSO-d_6.

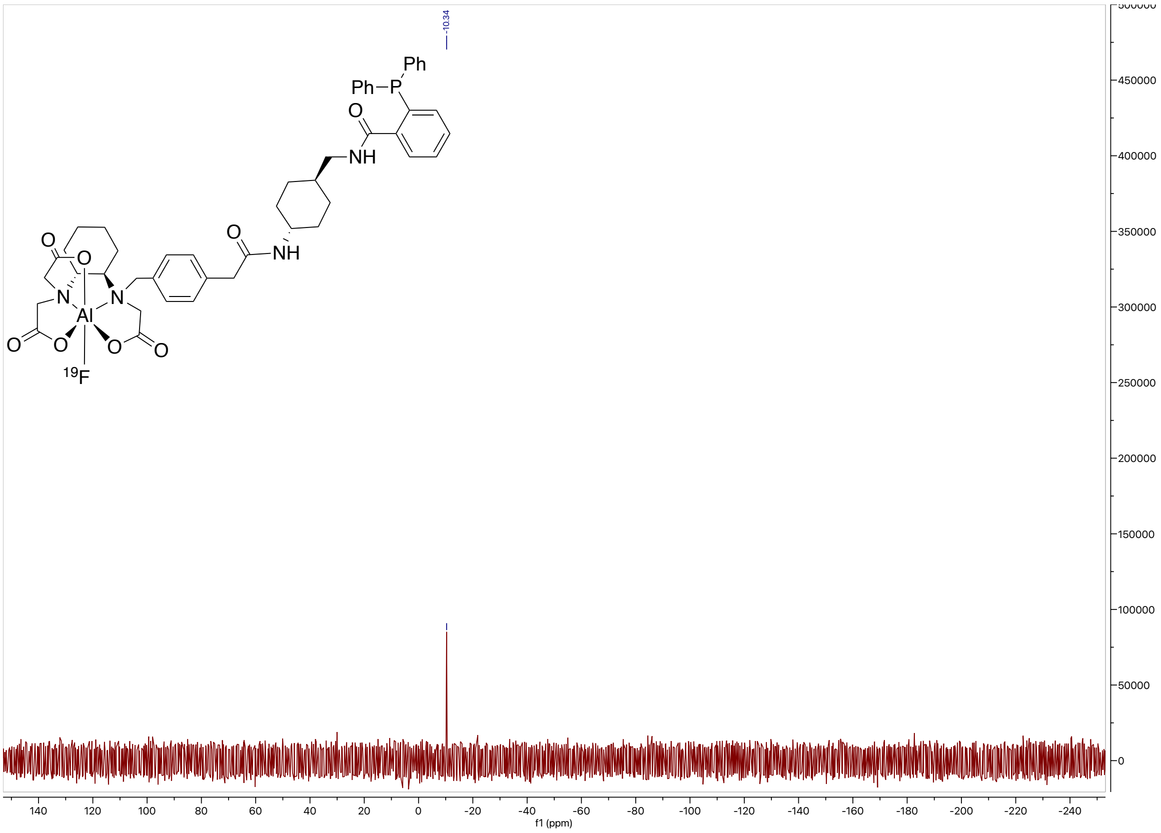

Fig. S44. ^{31}P NMR spectrum of compound **Al^{19}F-2c** in DMSO-d_6.

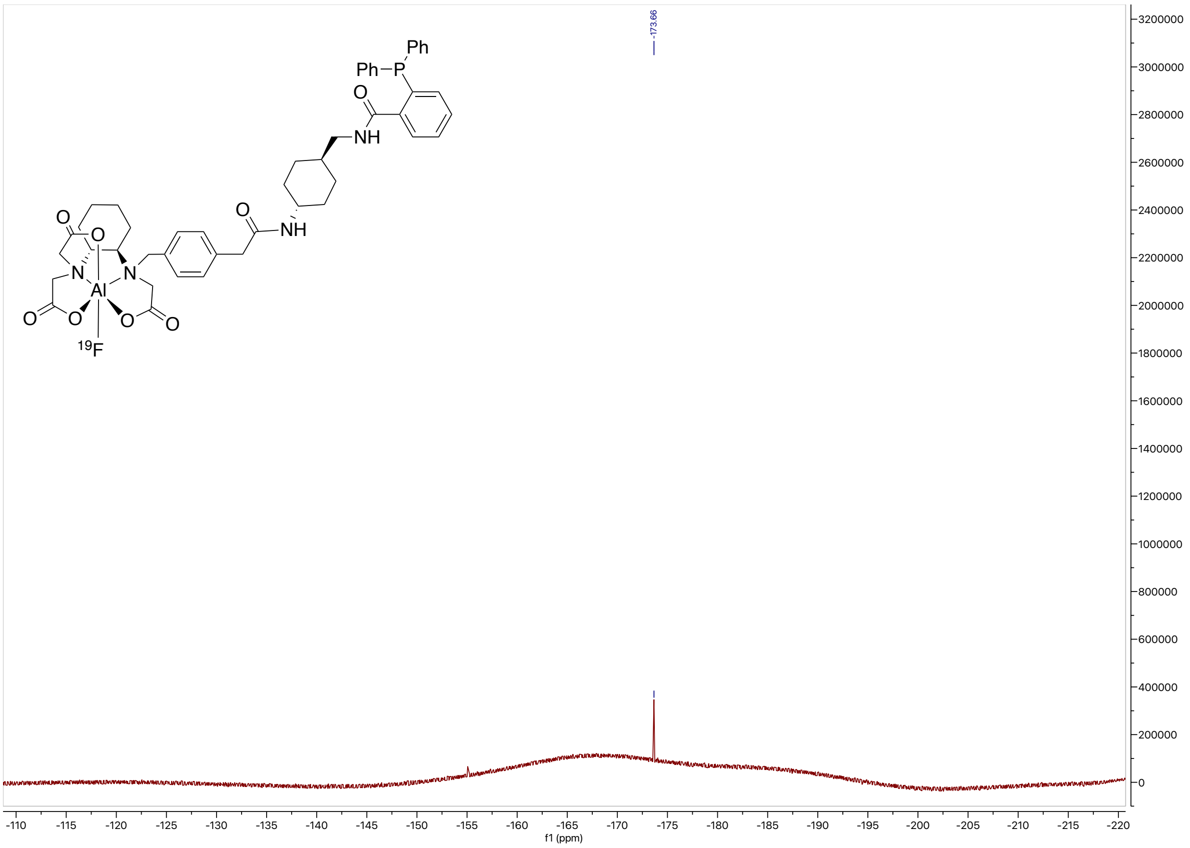

Fig. S45. ^{19}F NMR spectrum of compound **Al^{19}F-2c** in DMSO-d_6.

HRMS analysis

Samples were prepared by dilution methanol or THF stock solutions in 20:80 ultra-pure water/methanol to obtain concentrations 1 µg/mL for futher infusion with a syringe pump (CHEMYX Fusion 100T) and 500 µL Hamilton syringe at flow of 5 µL/min.

Orbitrap MS with the heated electrospray ion source was operated in negative polarity ion mode. Spray voltage was set to -3 or -3.5 kV and capillary temperature to 320 °C. Nitrogen gas was used as a sheath gas with a flow rate 10 arbitrary units. S-lens radio frequency level was set to 100. Resolution was set to 240 000, automatic gain control to 1 x 10^6, maximum injection time to 100 or 200 ms. External calibration was done using Pierce ESI Negative Ion Calibration Solution (Thermo Fisher Scientific Inc., Rockford, USA). Data was processed with Thermo Xcalibur Qual Browser (version 4.5.474.0). The error between the measured and theoretical exact mass was calculated and reported in parts per million (ppm) (**Table S1**).

Table S1. HRMS characterization data for final compounds **1**, **2a–2c**, **2c-oxide** and complexes **Al^{19}F-2c** and **Al^{19}F-2c-oxide**.

Compound	Formula	Observed ions	Calculated m/z	Measured m/z	mass error (ppm)
1	$C_{21}H_{20}F_4N_4O_{10}$	$[M-H]^-$	563.1043	563.1044	-0.18
2a	$C_{48}H_{55}N_4O_{13}P$	$[M-H]^-$	925.3430	925.3427	0.35
2b	$C_{42}H_{47}N_4O_8P$	$[M-H]^-$	765.3059	765.3055	0.52
2c	$C_{47}H_{55}N_4O_8P$	$[M-H]^-$	833.3685	833.3696	-1.32
2c-oxide	$C_{47}H_{55}N_4O_9P$	$[M-H]^-$	849.3634	849.3643	-1.06
Al^{19}F-2c	$C_{47}H_{52}AlFN_4O_8$	$[M]^-$	877.3328	877.3345	-1.94
Al^{19}F-2c-oxide	$C_{47}H_{52}AlFN_4O_9P$	$[M]^-$	893.3277	893.3276	-0.11

Supplementary references

1. Sundhoro M, Jeon S, Park J, Ramström O, Yan M. Perfluoroaryl Azide Staudinger Reaction: A Fast and Bioorthogonal Reaction. Angew Chem Int Ed Engl. 2017;56:12117-21. doi:https://doi.org/10.1002/anie.201705346.
2. Cole CM, Yang J, Šečkutė J, Devaraj NK. Fluorescent live-cell imaging of metabolically incorporated unnatural cyclopropene-mannosamine derivatives. Chembiochem. 2013;14:205-8. doi:https://doi.org/10.1002/cbic.201200719.
3. Kennedy AJ, Bruce AM, Gineste C, Ballard TE, Olekhnovich IN, Macdonald TL, et al. Synthesis and Antimicrobial Evaluation of Amixicile-Based Inhibitors of the Pyruvate-Ferredoxin Oxidoreductases of Anaerobic Bacteria and Epsilonproteobacteria. Antimicrob Agents Chemother. 2016;60:3980-7. doi:https://doi.org/doi:10.1128/AAC.00670-16.
4. Patterson JR, Terrell LR, Donatelli CA, Holt DA, Jolivette LJ, Rivero RA, et al. Design and Optimization of an Acyclic Amine Series of TRPV4 Antagonists by Electronic Modulation of Hydrogen Bond Interactions. J Med Chem. 2020;63:14867-84. doi:https://doi.org/10.1021/acs.jmedchem.0c01303.
5. Cepanec I, Litvić M, Mikuldaš H, Bartolinčić A, Vinković V. Calcium trifluoromethanesulfonate-catalysed aminolysis of epoxides. Tetrahedron. 2003;59:2435-9. doi:https://doi.org/10.1016/S0040-4020(03)00292-8.
6. Bruch A, Gebert A, Breit B. Tandem-Directed Regioselective Hydroformylation/β-Elimination: A Practical Method for the Synthesis of Enals. Synthesis. 2008;2008:2169-76. doi:https://doi.org/10.1055/s-2008-1067140.
7. Vugts DJ, Vervoort A, Stigter-van Walsum M, Visser GWM, Robillard MS, Versteegen RM, et al. Synthesis of Phosphine and Antibody–Azide Probes for in Vivo Staudinger Ligation in a Pretargeted Imaging and Therapy Approach. Bioconjug Chem. 2011;22:2072-81. doi:https://doi.org/10.1021/bc200298v.
8. Laye C, Lusseau J, Robert F, Landais Y. The Trityl-Cation Mediated Phosphine Oxides Reduction. Adv Synth Catal. 2021;363:3035-43. doi:https://doi.org/10.1002/adsc.202100189.
